# Survival After Hyperthermic Intraperitoneal Chemotherapy and Primary or Interval Cytoreductive Surgery in Ovarian Cancer: A Randomized Clinical Trial

**DOI:** 10.1001/jamasurg.2022.0143

**Published:** 2022-03-09

**Authors:** Myong Cheol Lim, Suk-Joon Chang, Boram Park, Heon Jong Yoo, Chong Woo Yoo, Byung Ho Nam, Sang-Yoon Park

**Affiliations:** 1 Center for Gynecologic Cancer, National Cancer Center, Goyang, South Korea; 2 Center for Clinical Trial, National Cancer Center, Goyang, South Korea; 3 Division of Rare and Refractory Cancer, Research Institute, National Cancer Center, Goyang, South Korea; 4 Department of Cancer Control and Policy, National Cancer Center, Goyang, South Korea; 5 Department of Obstetrics and Gynecology, Ajou University School of Medicine, Suwon, South Korea; 6 Biostatistics Collaboration Team, National Cancer Center, Goyang, South Korea; 7 Biomedical Statistics Center, Research Institute for Future Medicine, Samsung Medical Center, Seoul, South Korea; 8 Chungnam National University School of Medicine, Dajeon, South Korea; 9 HERINGS, Seoul, South Korea

## Abstract

**Importance:**

Ovarian cancer has the highest mortality rate among gynecologic malignant tumors. Data are lacking on the survival benefit of hyperthermic intraperitoneal chemotherapy (HIPEC) in women with ovarian cancer who underwent primary or interval cytoreductive surgery.

**Objective:**

To assess the clinical benefit of HIPEC after primary or interval maximal cytoreductive surgery in women with stage III or IV primary advanced ovarian cancer.

**Design, Setting, and Participants:**

In this single-blind randomized clinical trial performed at 2 institutions in South Korea from March 2, 2010, to January 22, 2016, a total of 184 patients with stage III or IV ovarian cancer with residual tumor size less than 1 cm were randomized (1:1) to a HIPEC (41.5 °C, 75 mg/m^2^ of cisplatin, 90 minutes) or control group. The primary end point was progression-free survival. Overall survival and adverse events were key secondary end points. The date of the last follow-up was January 10, 2020, and the data were locked on February 17, 2020.

**Exposures:**

Hyperthermic intraperitoneal chemotherapy after cytoreductive surgery.

**Main Outcomes and Measures:**

Progression-free and overall survival.

**Results:**

Of the 184 Korean women who underwent randomization, 92 were randomized to the HIPEC group (median age, 52.0 years; IQR, 46.0-59.5 years) and 92 to the control group (median age, 53.5 years; IQR, 47.5-61.0 years). After a median follow-up of 69.4 months (IQR, 54.4-86.3 months), median progression-free survival was 18.8 months (IQR, 13.0-43.2 months) in the control group and 19.8 months (IQR, 13.7-55.4 months) in the HIPEC group (*P* = .43), and median overall survival was 61.3 months (IQR, 34.3 months to not reported) in the control group and 69.5 months (IQR, 45.6 months to not reported) in the HIPEC group (*P* = .52). In the subgroup of interval cytoreductive surgery after neoadjuvant chemotherapy, the median progression-free survival was 15.4 months (IQR, 10.6-21.1 months) in the control group and 17.4 months (IQR, 13.8-31.5 months) in the HIPEC group (hazard ratio for disease progression or death, 0.60; 95% CI, 0.37-0.99; *P* = .04), and the median overall survival was 48.2 months (IQR, 33.8-61.3 months) in the control group and 61.8 months (IQR, 46.7 months to not reported) in the HIPEC group (hazard ratio, 0.53; 95% CI, 0.29-0.96; *P* = .04). In the subgroup of primary cytoreductive surgery, median progression-free survival was 29.7 (IQR, 17.2-90.1 months) in the control group and 23.9 months (IQR, 12.3-71.5 months) in the HIPEC group, and the median overall survival was not reached in the control group and 71.3 months (IQR, 45.6 months to not reported) in the HIPEC group.

**Conclusions and Relevance:**

The addition of HIPEC to cytoreductive surgery did not improve progression-free and overall survival in patients with advanced epithelial ovarian cancer. Although the results are from a subgroup analysis, the addition of HIPEC to interval cytoreductive surgery provided an improvement of progression-free and overall survival.

**Trial Registration:**

ClinicalTrials.gov Identifier: NCT01091636

## Key Points

**Question:**

Does hyperthermic intraperitoneal chemotherapy (HIPEC) after primary or interval cytoreductive surgery increase survival in patients with ovarian cancer?

**Findings:**

In this randomized clinical trial of 184 women with ovarian cancer, among those who underwent interval cytoreductive surgery after neoadjuvant chemotherapy, the addition of HIPEC decreased recurrence and increased overall survival; however, in patients undergoing primary cytoreductive surgery, progression-free survival and overall survival were not improved. No unresolved serious HIPEC-related adverse events were found in either group.

**Meaning:**

These results suggest that HIPEC after interval cytoreductive surgery may increase progression-free and overall survival in patients with ovarian cancer who receive neoadjuvant chemotherapy.

## Teaser

This randomized clinical trial of women with ovarian cancer compares the outcomes of hyperthermic intraperitoneal chemotherapy after primary vs interval cytoreductive surgery.

## Introduction

Women with ovarian, fallopian, and primary peritoneal cancer are frequently diagnosed with peritoneal carcinomatosis, which has the highest mortality rate among gynecologic malignant tumors with an age-standardized, 5-year net survival rate of 37% to 43%.^1,2^ The standard treatment involves maximal cytoreductive surgery followed by adjuvant chemotherapy to minimize the residual tumor size.^3^ Moreover, neoadjuvant chemotherapy can also be used in selected patient populations with severe medical illness or unresectable tumor burden.^3^ Intraperitoneal chemotherapy increases progression-free and overall survival among patients with ovarian cancer who undergo cytoreductive surgery for a residual tumor size of less than 1 cm.^3,4^ The survival benefits of intraperitoneal chemotherapy extend for more than 10 years.^5^ However, despite the long-term survival benefits, port-related adverse effects, abdominal pain, and additional effort required for the management of an intraperitoneal catheter limit the use of intraperitoneal chemotherapy.^6^

Hyperthermic intraperitoneal chemotherapy (HIPEC) involves intraperitoneal chemotherapy, which does not require a postoperative intraperitoneal port and is accompanied with heated chemotherapeutic agents that are administered immediately after cytoreductive surgery. The intraoperative administration of HIPEC before any surgical adhesions can confer several potential treatment benefits. Several retrospective studies^7,8^ have suggested the survival benefits of HIPEC in primary and recurrent ovarian cancer. The results from a randomized trial^9^ (Interval Debulking Surgery +/− Hyperthermic Intraperitoneal Chemotherapy in Stage III Ovarian Cancer [OVHIPEC-01]) indicated the clinical benefit of HIPEC after interval cytoreductive surgery subsequent to neoadjuvant chemotherapy for stage III primary ovarian cancer with regard to decreased recurrence and mortality rates. However, further evidence from a clinical trial is needed to resolve several issues, including the lack of a selection flowchart after neoadjuvant chemotherapy, disproportionate histologic types, a bias in surgical radicality, or underreported adverse events, found in a previous study.^10^ Furthermore, the clinical benefit of HIPEC in an extended study population, including patients with stage IV primary ovarian cancer and those who have undergone primary cytoreductive surgery, needs to be investigated.

Following the confirmation of the feasibility and safety of HIPEC after primary or interval maximal cytoreductive surgery from a phase 2 study^11^ in 2009, the current randomized clinical trial was initiated in 2010. This trial was conducted to assess the clinical benefit of HIPEC after primary or interval maximal cytoreductive surgery in women with stage III or IV primary advanced ovarian cancer.

## Methods

### Study Design

This single-blind randomized clinical trial was conducted at 2 institutions in South Korea (National Cancer Center and Ajou University Hospital) from March 2, 2010, to January 22, 2016. The date of the last follow-up was January 10, 2020, and the data were locked on February 17, 2020. This trial was approved by the institutional review boards of both institutions. The study was conducted in accordance with the Declaration of Helsinki.^12^ Written informed consent for this trial was preoperatively obtained from women who were eligible for primary or interval cytoreductive surgery. The trial protocol can be found in Supplement 1. This study followed the Consolidated Standards of Reporting Trials (CONSORT) reporting guideline.

### Study Participants

**Figure 1.**
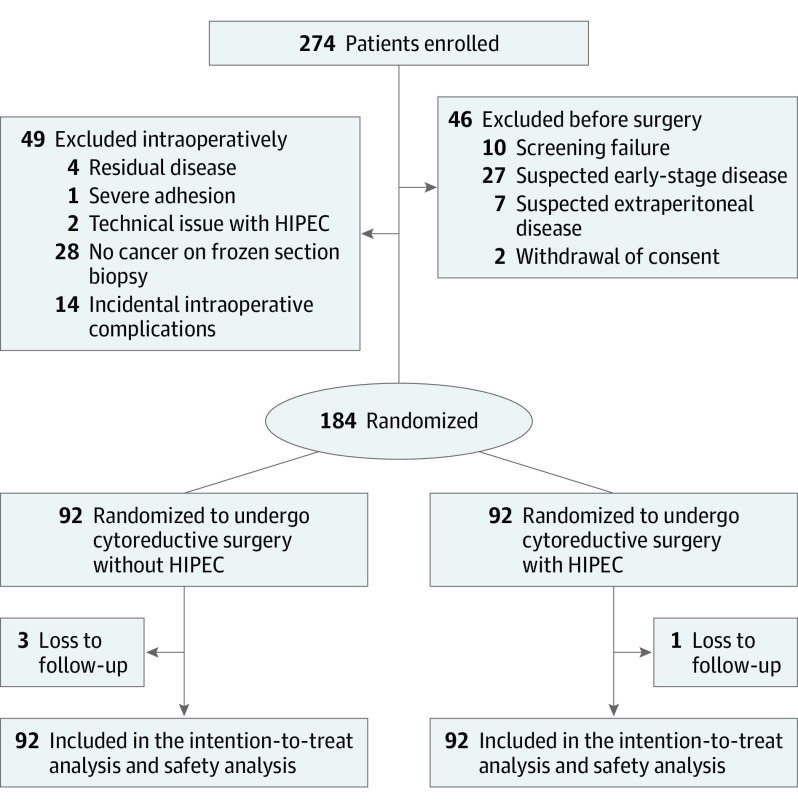
Flowchart of Enrollment and Randomization HIPEC indicates hyperthermic intraperitoneal chemotherapy.

Eligible patients were younger than 75 years with newly diagnosed advanced (Internal Federation of Gynecology and Obstetrics stage III or IV) epithelial ovarian, primary peritoneal, or fallopian tube cancer. Neoadjuvant chemotherapy was offered based on the judgment of the surgeon in conformance with the institutional criteria using computed tomographic findings and performance.^13^ Randomization of the study participants is shown in Figure 1. After 3 cycles of neoadjuvant chemotherapy with carboplatin (area under the curve of 5 mg/mL per minute) and paclitaxel (175 mg/m^2^ of the body surface area), patients were assessed for a partial response and stable disease. All patients who had an Eastern Cooperative Oncology Group performance status of 0 or 1, residual tumor smaller than 1 cm, age older than 75 years, and adequate hematologic function (white blood cell count of ≥3000/μL [to convert to ×10^9^/L, multiply by 0.001] and platelet count of ≥100.0 × 10^3^/μL [to convert to ×10^9^/L, multiply by 1]), liver function (serum bilirubin level ≤1.5 mg/dL [to convert to micromoles per liter, multiply by 17.104] and alanine aminotransferase, aspartate aminotransferase, and alkaline phosphatase levels ≤80 IU/L [to convert to microkatals per liter, multiply by 0.0167]), and renal function (creatinine level ≤1.5 mg/dL [to convert to micromoles per liter, multiply by 88.4]) were included in this study. The main exclusion criteria were unresectable extraperitoneal metastasis (brain, bone, lung parenchyma, or lymph node); residual tumor 1 cm or larger; previous other malignant tumors; serious heart, kidney, or pulmonary insufficiency; pregnant or breastfeeding; considered unsuitable by a physician; or no pathological diagnosis of cancer during cytoreductive surgery after neoadjuvant chemotherapy.

Both primary and interval cytoreductive surgical procedures were offered as treatment options in these patients.^11,13^ The vaginal stump and resected diaphragm were closed to ensure a water-tight fit, and bowel anastomosis was completed before randomization.

### Setting

An independent statistical center randomly assigned the participants to the HIPEC group or the control group. Participants were blinded to the group allocation. Patients were intraoperatively randomized into the HIPEC and control groups in a 1:1 manner at the completion of cytoreductive surgery if the estimated residual tumor size was less than 1 cm. Intraoperative HIPEC (75 mg/m^2^ of cisplatin) was perfused through a closed technique with a target temperature of 41.5 °C for 90 minutes using the Belmont Hyperthermia Pump system (Belmont Instrument Corporation), as previously reported.^11^ Women randomized to the HIPEC group received blanket cooling, intravenous cold fluid hydration, and ice pack application over the head before and during HIPEC procedures. After the cytoreductive and reconstructive surgical procedures, 2 inflow and 2 outflow tubes were placed in the pelvic cavity and in the subdiaphragmatic space, respectively. The abdominal wall was closed in layers with a water-tight fit, and 0.9% normal saline was injected into the closed abdominal cavity. After smooth circulation to and from the HIPEC pump was confirmed, the chemotherapeutic agent was mixed with the circulating fluid. During the 90-minute HIPEC perfusion procedure, the patients were gently shaken from side to side to ensure even distribution of the chemotherapeutic agent within the peritoneal cavity. Sodium thiosulfate was not used in the initial 71 cases, given the low incidence of serum creatinine elevation in the phase 2 study.^11^ However, in the remaining 21 patients, 4 g/m^2^ of sodium thiosulfate was administered as a bolus infusion immediately before HIPEC, and 12 g/m^2^ was administered over 6 hours during and after the HIPEC procedures. Four intraperitoneal thermometer readings for each abdominal quadrant, a nasopharyngeal temperature probe, and manual tube checks were undertaken at 5-minute intervals to monitor the peritoneal and core body temperatures. During postoperative recovery, if the patients could tolerate a general diet without evidence of active infection and with an acceptable clinical condition to sustain chemotherapy, we administered 6 cycles of intravenous paclitaxel and carboplatin in both groups. After completion of adjuvant chemotherapy, follow-up with the CA125 test and computed tomography was conducted every 3 months for 2 years, every 6 months for up to 5 years, and yearly thereafter.

### Outcomes

Progression was defined by a clinical decision that was based on radiologic findings of tumor growth (Response Evaluation Criteria in Solid Tumors criteria 1.1) or biochemical assessment of the Gynecologic Cancer InterGroup CA125 criteria, whichever occurred first.^14,15^ The primary end point was progression-free survival, which was defined as the time from the date of randomization to the date of disease progression or death from any cause, whichever occurred first. Secondary end points included overall survival and adverse events. Overall survival was defined as the time from randomization to the date of death from any cause. Survival data were censored at the date of the last follow-up. An adverse event was evaluated using the Common Terminology Criteria for Adverse Events from randomization to the initiation of the first adjuvant chemotherapy and thereafter to 6 weeks after the last adjuvant chemotherapy.

### Sample Size

A log-rank test with an overall sample size of 184 individuals (92 in the control group and 92 in the treatment group) achieves 55.1% and 81.0% power at a *P* < .05 significance level to detect hazard ratios (HRs) of 0.75 and 0.66 when median survival time of the control group is 18.0 months.^16^ The number of events required is 150 patients in the total set (80 in the control group and 70 in the treatment group). The total study period was 8 years, allowing 6 years as an accrual period and 2 years as a follow-up period after the last patients were accrued.

### Statistical Analysis

All analyses were performed on the intention-to-treat population. The Kaplan-Meier method and log-rank test were used to estimate and compare survivals between the 2 groups. The Cox proportional hazards regression model was used to estimate HRs and 95% CIs in univariable and multivariable models. Subgroup analysis was performed according to the use of neoadjuvant chemotherapy. The additional subgroup analysis results were reported with HRs (95% CIs) using a forest plot. Differences for adverse events were calculated using the proportion of the HIPEC group minus the proportion of the control group. Differences and 95% CIs were presented as percentiles. A 2-sided *P* < .05 was considered statistically significant, and all statistical analyses were conducted using SAS software, version 9.4 (SAS Institute Inc) and R software, version 3.6.2 (R Foundation for Statistical Computing).

## Results

### Patients and Cytoreductive Surgery With or Without HIPEC

Of the 184 Korean women who underwent randomization, 92 were randomized to the HIPEC group (median age, 52.0 years; IQR, 46.0-59.5 years) and 92 to the control group (median age, 53.5 years; IQR, 47.5-61.0 years). The baseline demographic and clinical characteristics were similar in the 2 groups (Table 1), with the exception of an additional 2 hours of operative time for the HIPEC procedures (405 vs 525 minutes). eTable 3 in Supplement 2 lists the operative procedures undertaken for ovarian cancer. No intergroup differences in operative procedures and surgical outcomes were found. Ileostomy was performed in 7 patients (7.6%) in the HIPEC group and 6 patients (6.5%) in the control group.

**Table 1.** Baseline Patient Characteristics and Treatment-Related Variables^a^

Variable	Total		Cytoreductive surgery			
	Control (n = 92)	HIPEC (n = 92)	Primary		Interval	
			Control (n = 49)	HIPEC (n = 58)	Control (n = 43)	HIPEC (n = 34)
Age, median (IQR), y	53.5 (47.5-61.0)	52.0 (46-59.5)	53.0 (47.0-61.0)	51.0 (45.0-58.0)	54.0 (48.0-61.0)	55.0 (47.0-64.0)
Serum albumin, median (IQR), g/dL	4.3 (4-4.5)	4.3 (4-4.6)	4.1 (3.9-4.4)	4.1 (3.8-4.6)	4.4 (4.1-4.6)	4.4 (4.1-4.7)
FIGO stage^b^						
III	51 (55.4)	60 (65.2)	34 (69.4)	45 (77.6)	17 (39.5)	15 (44.1)
IV	41 (44.6)	32 (34.8)	15 (30.6)	13 (22.4)	26 (60.5)	19 (55.9)
Histologic type						
Serous	79 (85.9)	85 (92.4)	41 (83.7)	53 (91.4)	38 (88.4)	32 (94.1)
Endometrioid	5 (5.4)	3 (3.3)	3 (6.1)	3 (5.2)	2 (4.7)	0
Clear cell	4 (4.4)	0	3 (6.1)	0	1 (2.3)	0
Others	4 (4.4)	4 (4.4)	2 (4.1)	2 (3.5)	2 (4.7)	2 (5.9)
Neoadjuvant chemotherapy						
No	49 (53.3)	58 (63.0)	49 (100)	58 (100)	0	0
Yes	43 (46.7)	34 (37.0)	0	0	43 (100)	34 (100)
Peritoneal carcinomatosis index score^c^						
0-5	29 (31.5)	22 (23.9)	16 (32.7)	14 (24.1)	13 (30.2)	8 (23.5)
6-10	63 (68.5)	70 (76.1)	33 (67.4)	44 (75.9)	30 (69.8)	26 (76.5)
Residual disease after cytoreductive surgery						
Microscopic	80 (87.0)	75 (81.5)	43 (87.8)	48 (82.8)	37 (86.0)	27 (79.4)
Macroscopic	12 (13.0)	17 (18.5)	6 (12.2)	10 (17.2)	6 (14.0)	7 (20.6)
Bowel surgery						
No	24 (26.1)	19 (20.7)	11 (22.5)	7 (12.1)	13 (30.2)	12 (35.3)
Yes	68 (73.9)	73 (79.4)	38 (77.6)	51 (87.9)	30 (69.8)	22 (64.7)
Rectosigmoid resection						
No	30 (32.6)	28 (30.4)	15 (30.6)	15 (25.9)	15 (34.9)	13 (38.2)
Yes	62 (67.4)	64 (69.6)	34 (69.4)	43 (74.1)	28 (65.1)	21 (61.8)
Time of operation, median (IQR), min	405.0 (330.5-476.5)	525.0 (463.5-575.0)	426.0 (340.0-500.0)	529.5 (486.0-577.0)	384.0 (328.0-437.0)	506.5 (449.0-570.0)
Duration of hospital stay, median (IQR), d	14 (12-23.5)	17 (13-23)	15 (13-29)	16 (14-22)	14 (12-20)	17 (13-27)
Time between surgery and initiation of the adjuvant chemotherapy, median (IQR), d	20 (18-27)	22 (19-25)	23 (19-27)	21 (19-26)	20 (16-24)	22 (19-24)
Time between end of first chemotherapy session and initiation of the adjuvant chemotherapy, median (IQR), d	111 (105-123)	112 (106-122)	113 (105-123)	113 (107-122)	108 (105-120)	111 (105-119)

Abbreviations: FIGO, Federation of Gynecology and Obstetrics; HIPEC, hyperthermic intraperitoneal chemotherapy.

SI conversion factor: To convert albumin to grams per liter, multiply by 10.

^a^ Data are presented as number (percentage) of patients unless otherwise indicated.

^b^ Details on the International FIGO staging system are provided in eTable 1 in the Supplement.

^c^ Details on the peritoneal carcinomatosis index are provided in eTable 2 in the Supplement.

### Outcomes

**Figure 2.**
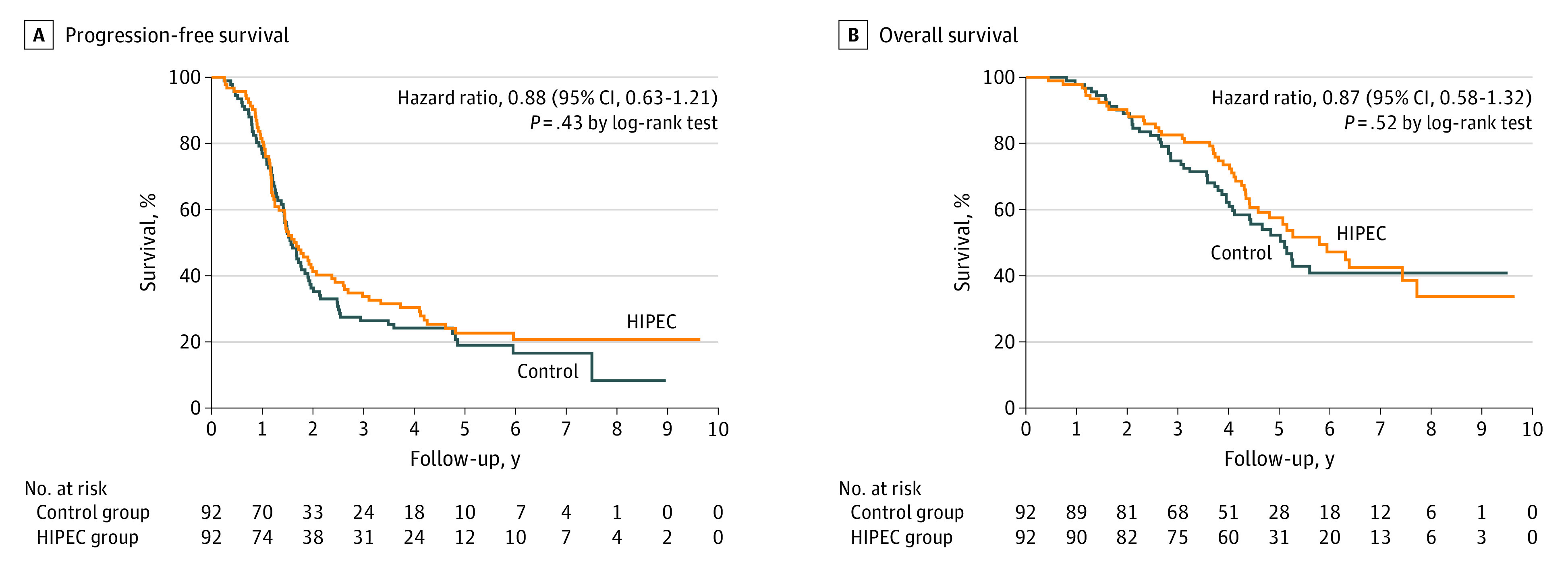
Kaplan-Meier Estimates of Progression-Free Survival and Overall Survival as Preplanned Intention to Treat A, Events of progression or death were observed in 74 patients (80.4%) in the control group and in 71 patients (77.2%) in the hyperthermic intraperitoneal chemotherapy (HIPEC) group. The Kaplan-Meier estimate of patients who were without progression and alive at 24 months was 36.3% in the control group and 41.3% in the HIPEC group. B, A total of 47 patients (51.1%) in the surgery group and 45 (48.9%) patients in the HIPEC group died. The Kaplan-Meier estimate of patients who were alive at 60 months was 52.3% in the control group and 57.5% in the HIPEC group.

In the current analysis, 145 progressions occurred in the overall cohort, in 71 of 92 patients (77.2%) in the HIPEC group and 74 of 92 patients (80.4%) in the control group. The median follow-up duration at the time of data cutoff was 69.4 months (IQR, 54.4-86.3 months) for all patients in the intention-to-treat population and was similar for the HIPEC (69.4 months; IQR, 55.6-92.1 months) and control (70.8 months; IQR, 53.6-85.8 months) groups. The longest follow-up at the time of the database locking was 115.7 months. Moreover, among the randomized patients, 87 (94.6%) underwent HIPEC. The median progression-free survival was 19.8 months (IQR, 13.7-55.4 months) in the HIPEC group and 18.8 months (IQR, 13.0-43.2 months) in the control group, and the median overall survival was 69.5 months (IQR, 45.6 months to not reported) in the HIPEC group and 61.3 months (IQR, 34.3 months to not reported) in the control group (Figure 2).

**Figure 3.**
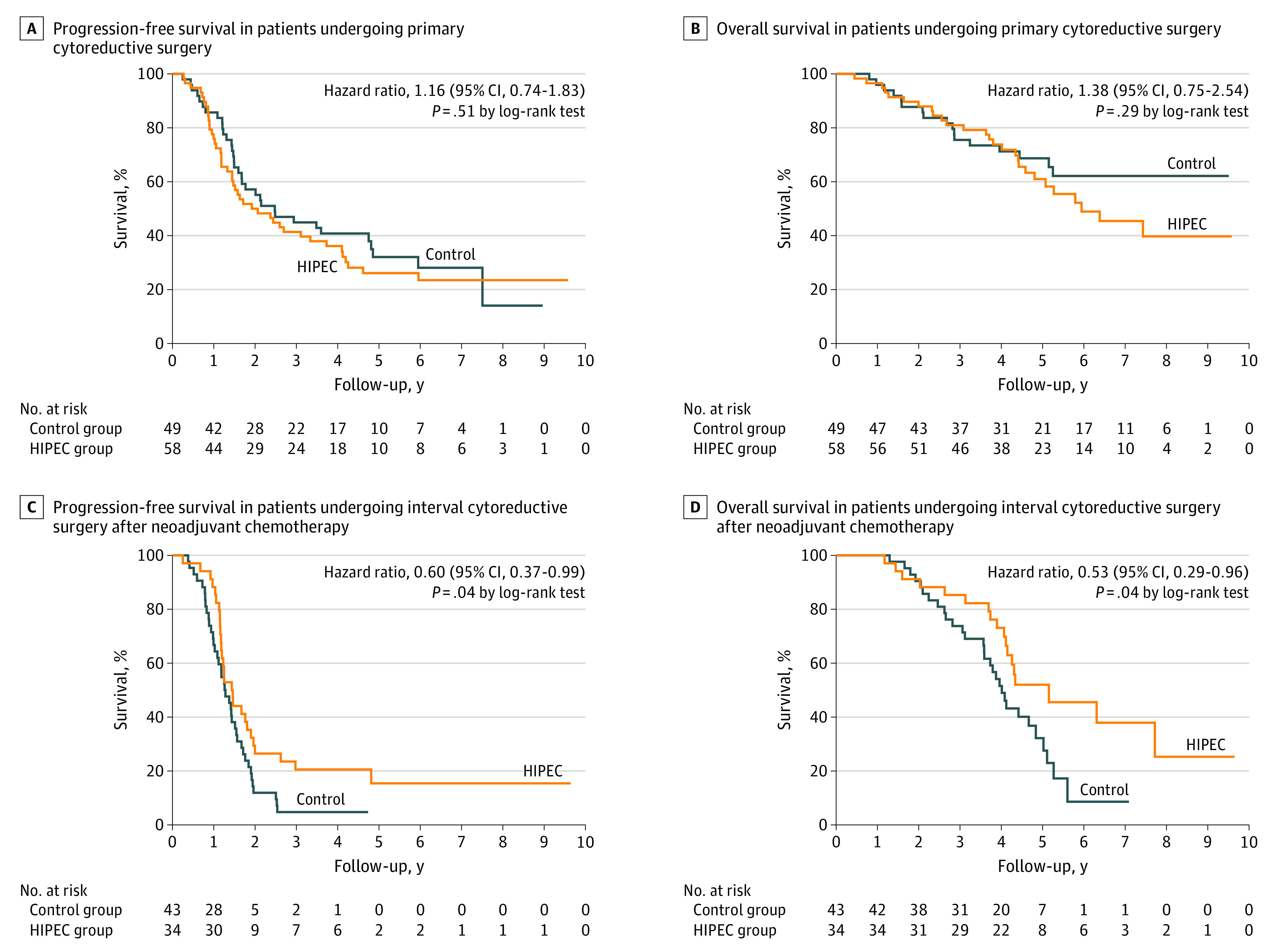
Kaplan-Meier Estimates of Progression-Free Survival and Overall Survival According to the Primary Treatment as Preplanned Intention to Treat Among the patients undergoing primary cytoreductive surgery prespecified subgroup analysis, the Kaplan-Meier estimate of patients who were free of progression and death at 24 months was 57.1% in the control group and 50% in the hyperthermic intraperitoneal chemotherapy (HIPEC) group (A), and the Kaplan-Meier estimate of patients who were alive at 60 months was 68.7% in the control group and 61% in the HIPEC group (B). Among the patients undergoing interval cytoreductive surgery after neoadjuvant chemotherapy prespecified subgroup analysis, the Kaplan-Meier estimate of patients who were free of progression and death at 24 months was 11.9% in the control group and 26.5% in the HIPEC group (C), and the Kaplan-Meier estimate of patients who were alive at 60 months was 32.2% in the control group and 52% in the HIPEC group (D).

In patients who underwent interval cytoreductive surgery after neoadjuvant chemotherapy, the median progression-free survival was 17.4 months (IQR, 13.8-31.5 months) in the HIPEC group and 15.4 months (IQR, 10.6-21.1 months) in the control groups (HR for disease progression or death, 0.60; 95% CI, 0.37-0.99; *P* = .04). The median overall survival was 61.8 months (IQR, 46.7 months to not reported) in the HIPEC group and 48.2 months (IQR, 33.8-61.3 months) in the control group (HR, 0.53; 95% CI, 0.29-0.96; *P* = .04). In patients who underwent primary cytoreductive surgery (Figure 3), the median progression-free survival was 23.9 months (IQR, 12.3-71.5 months) in the HIPEC group and 29.7 months (IQR, 17.2-90.1 months) in the control groups. The median overall survival was 71.3 months (IQR, 45.6 months to not reported) in the HIPEC group and was not reached in the control group.

Assessment of the predefined subgroup of the total patients with postneoadjuvant chemotherapy HIPEC and an interval of 3 weeks or less between surgery and initiation of the adjuvant chemotherapy yielded HRs of 0.60 (95% CI, 0.37-0.99) and 0.59 (95% CI, 0.37-0.94) in the HIPEC group compared with the control group (eFigure 2 in Supplement 2). In patients with primary cytoreductive surgery, no subgroup differences were meaningful for progression-free and overall survival (eFigure 3 in Supplement 2). In the assessment of patients with postneoadjuvant chemotherapy HIPEC (eFigure 4 in Supplement 2), the results of the subgroup analyses for progression-free survival showed a benefit for younger age (HR, 0.39; 95% CI, 0.19-0.83; *P* = .01), high-grade serous histologic type (HR, 0.49; 95% CI, 0.28-0.88; *P* = .02), lower peritoneal carcinomatosis index (HR, 0.22; 95% CI, 0.06-0.79; *P* = .02), lower residual tumor (HR, 0.52; 95% CI, 0.30-0.91; *P* = .02), no bowel surgery (HR, 0.35; 95% CI, 0.14-0.88; *P* = .03), and no rectosigmoid resection (HR, 0.38; 95% CI, 0.16-0.91; *P* = .03). Baseline characteristics, including age, serum albumin, stage, histologic type, bowel surgery, or rectosigmoid resection, were not significant for survival outcomes in the multivariable Cox proportional hazards regression model (eTable 5 in Supplement 2).

### Safety

At least 1 adverse event of any grade occurred in all study participants after randomization until 6 weeks after the last chemotherapy (Table 2). During HIPEC, no intraprocedural adverse events occurred that necessitated the discontinuation of the procedure. In addition, no HIPEC-related deaths occurred. Grade 3 or 4 adverse events were reported in 86 patients (93.5%) in the HIPEC group and 80 patients (87.0%) in the control group.

**Table 2.** Adverse Events From Randomization to the 6 Weeks After Postoperative Adjuvant Chemotherapy^a^

Adverse event	Any grade			Grade 3 or 4		
	No. (%)		Difference, % (95% CI)^b^	No. (%)		Difference, % (95% CI)^b^
	Control (n = 92)	HIPEC (n = 92)		Control (n = 92)	HIPEC (n = 92)	
Anemia	92 (100)	92 (100)	0	44 (47.8)	52 (56.5)	8.7 (−5.7 to 23.1)
Electrolyte disturbance	92 (100)	92 (100)	0	41 (44.6)	74 (80.4)	35.9 (22.9 to 48.9)
Abdominal pain	92 (100)	92 (100)	0	0	0	0
Hyperaminotransferase	77 (83.7)	78 (84.8)	1.1 (−9.4 to 11.6)	18 (19.6)	8 (8.7)	−10.9 (−20.8 to −0.9)
Peripheral sensory neuropathy	79 (85.9)	78 (84.8)	−1.1 (−11.3 to 9.1)	0	0	0
Lymphocele	84 (91.3)	76 (82.6)	−8.7 (−18.3 to 1)	12 (13)	16 (17.4)	4.3 (−6 to 14.7)
INR increased	60 (65.2)	75 (81.5)	16.3 (3.7 to 28.9)	2 (2.2)	1 (1.1)	−1.1 (−4.7 to 2.6)
Pulmonary	63 (68.5)	69 (75)	6.5 (−6.5 to 19.5)	5 (5.4)	8 (8.7)	3.3 (−4.1 to 10.7)
Nausea	63 (68.5)	68 (73.9)	5.4 (−7.6 to 18.5)	0	0	0
WBC count decreased	62 (67.4)	67 (72.8)	5.4 (−7.8 to 18.6)	22 (23.9)	24 (26.1)	2.2 (−10.3 to 14.7)
Neutrophil count decreased	53 (57.6)	63 (68.5)	10.9 (−3 to 24.7)	26 (28.3)	31 (33.7)	5.4 (−7.9 to 18.8)
Creatinine increased	44 (47.8)	63 (68.5)	20.7 (6.7 to 34.6)	2 (2.2)	2 (2.2)	0 (−4.2 to 4.2)
Anorexia	58 (63)	54 (58.7)	−4.3 (−18.4 to 9.7)	2 (2.2)	5 (5.4)	3.3 (−2.2 to 8.8)
Cardiac event	46 (50)	48 (52.2)	2.2 (−12.3 to 16.6)	0	2 (2.2)	2.2 (−0.8 to 5.2)
Infection	43 (46.7)	47 (51.1)	4.3 (−10.1 to 18.8)	25 (27.2)	27 (29.3)	2.2 (−10.8 to 15.2)
Insomnia	36 (39.1)	47 (51.1)	12 (−2.3 to 26.2)	1 (1.1)	2 (2.2)	1.1 (−2.6 to 4.7)
Diarrhea	38 (41.3)	43 (46.7)	5.4 (−8.9 to 19.8)	17 (18.5)	20 (21.7)	3.3 (−8.3 to 14.8)
Vomiting	35 (38)	38 (41.3)	3.3 (−10.9 to 17.4)	3 (3.3)	1 (1.1)	−2.2 (−6.4 to 2)
Constipation	40 (43.5)	35 (38)	−5.4 (−19.6 to 8.7)	1 (1.1)	0	−1.1 (−3.2 to 1)
Platelet count decreased	26 (28.3)	33 (35.9)	7.6 (−5.8 to 21.1)	8 (8.7)	8 (8.7)	0 (−8.1 to 8.1)
Hypertension	36 (39.1)	29 (31.5)	−7.6 (−21.4 to 6.2)	9 (9.8)	11 (12)	2.2 (−6.8 to 11.2)
Ileus	26 (28.3)	26 (28.3)	0 (−13 to 13)	8 (8.7)	9 (9.8)	1.1 (−7.3 to 9.5)
Blood bilirubin increased	28 (30.4)	26 (28.3)	−2.2 (−15.3 to 11)	4 (4.3)	2 (2.2)	−2.2 (−7.3 to 2.9)
Lymphedema	25 (27.2)	23 (25)	−2.2 (−14.9 to 10.5)	0	0	0
Wound dehiscence	25 (27.2)	22 (23.9)	−3.3 (−15.9 to 9.3)	10 (10.9)	14 (15.2)	4.3 (−5.4 to 14.1)
Acute kidney injury	6 (6.5)	19 (20.7)	14.1 (4.4 to 23.8)	3 (3.3)	2 (2.2)	−1.1 (−5.8 to 3.6)
Urticaria	19 (20.7)	19 (20.7)	0 (−11.7 to 11.7)	4 (4.3)	4 (4.3)	0 (−5.9 to 5.9)
Febrile neutropenia	10 (10.9)	9 (9.8)	−1.1 (−9.9 to 7.7)	10 (10.9)	9 (9.8)	−1.1 (−9.9 to 7.7)
Thromboembolic event	5 (5.4)	8 (8.7)	3.3 (−4.1 to 10.7)	0	1 (1.1)	1.1 (−1 to 3.2)
Depression	8 (8.7)	8 (8.7)	0 (−8.1 to 8.1)	1 (1.1)	0	−1.1 (−3.2 to 1)
Fistula, perforation, or leakage of bowel	0	7 (7.6)	7.6 (2.2 to 13)	0	5 (5.4)	5.4 (0.8 to 10.1)
Anal hemorrhage	2 (2.2)	6 (6.5)	4.3 (−1.5 to 10.2)	0	1 (1.1)	1.1 (−1 to 3.2)
Intra-abdominal hemorrhage	0	3 (3.3)	3.3 (−0.4 to 6.9)	0	1 (1.1)	1.1 (−1 to 3.2)
Sepsis	0	3 (3.3)	3.3 (−0.4 to 6.9)	0	3 (3.3)	3.3 (−0.4 to 6.9)
Fall	2 (2.2)	3 (3.3)	1.1 (−3.6 to 5.8)	0	1 (1.1)	1.1 (−1 to 3.2)
Colonic fistula	0	2 (2.2)	2.2 (−0.8 to 5.2)	0	2 (2.2)	2.2 (−0.8 to 5.2)
Colonic perforation	0	2 (2.2)	2.2 (−0.8 to 5.2)	0	2 (2.2)	2.2 (−0.8 to 5.2)
Large intestinal anastomotic leak	0	2 (2.2)	2.2 (−0.8 to 5.2)	0	1 (1.1)	1.1 (−1 to 3.2)
Delirium	2 (2.2)	2 (2.2)	0 (−4.2 to 4.2)	0	1 (1.1)	1.1 (−1 to 3.2)
Syncope	1 (1.1)	2 (2.2)	1.1 (−2.6 to 4.7)	1 (1.1)	2 (2.2)	1.1 (−2.6 to 4.7)
Urinary fistula	1 (1.1)	1 (1.1)	0 (−3 to 3)	0	1 (1.1)	1.1 (−1 to 3.2)
Duodenal perforation	0	1 (1.1)	1.1 (−1 to 3.2)	0	0	0
Jejunal hemorrhage	0	1 (1.1)	1.1 (−1 to 3.2)	0	1 (1.1)	1.1 (−1 to 3.2)
Seizure	0	1 (1.1)	1.1 (−1 to 3.2)	0	1 (1.1)	1.1 (−1 to 3.2)
Postoperative hemorrhage	1 (1.1)	0	−1.1 (−3.2 to 1)	1 (1.1)	0	−1.1 (−3.2 to 1)

Abbreviations: HIPEC, hyperthermic intraperitoneal chemotherapy; INR, international normalized ratio; WBC, white blood cell.

^a^ Data are given for adverse events that occurred in at least 1 patient in either trial group during the trial intervention or up to 6 weeks after adjuvant chemotherapy after cytoreductive surgery and are listed in descending order of frequency in the HIPEC group. The adverse events were graded according to the National Cancer Institute Common Terminology Criteria for Adverse Events, version 4.

^b^ Differences were calculated using the proportion of HIPEC group minus the proportion of control group.

With regard to any-grade adverse events, increased prothrombin time (75 [81.5%] vs 60 [65.2%]; *P* = .01) and acute kidney injury (19 [20.7%] vs 6 [6.5%]; *P* = .005) were prevalent in the HIPEC group compared with the control group. Electrolyte disturbance (74 [80.4%] vs 41 [44.6%]; *P* < .001) was prevalent as a grade 3 or 4 adverse event in the HIPEC group. Amifostine use decreased the incidence of elevated serum creatinine level (82% to 24%; *P* < .001) and acute kidney injury (27% to 0%; *P* = .005) in the HIPEC group (eTable 4 in Supplement 2).

## Discussion

This randomized clinical trial in patients with stage III or IV epithelial ovarian, fallopian tubal, and primary peritoneal cancer who underwent primary or interval cytoreductive surgery did not find a significant improvement of progression-free survival or overall survival between the HIPEC and control groups. This is the first trial, to our knowledge, to identify the clinical benefit of HIPEC after primary or interval cytoreductive surgery in primary advanced ovarian cancer (eFigure 1 in Supplement 2). In this study, a survival benefit from HIPEC was identified in women with primary stage III and IV epithelial ovarian cancer who underwent interval cytoreductive surgery after neoadjuvant chemotherapy. The current findings are consistent with the survival benefit reported in the OVHIPEC-01 trial despite the different setting of HIPEC with regard to the HIPEC technique (closed vs open), temperature (41.5 °C vs 40.0 °C), dose of the chemotherapeutic agent (75 mg/m^2^ with 100% of the dose perfused initially vs 100 mg/m^2^ with 50% of the dose perfused initially [25% at 30 minutes and 25% at 60 minutes]), and time of bowel anastomoses (before vs after HIPEC).^9^ The median duration of surgery was longer in this trial (507 minutes) compared with OVHIPEC-01 (338 minutes) because of the extensiveness of cytoreductive surgery. However, the adjuvant chemotherapy was initiated earlier in the current trial than in OVHIPEC-01 (22 vs 33 days). However, this trial did not identify a survival benefit of HIPEC in women with primary stage III and IV epithelial ovarian cancer who underwent primary cytoreductive surgery.

Hyperthermic intraperitoneal chemotherapy is considered a regional treatment for intraperitoneal disease. A previous randomized study^16^ of HIPEC focused on stage III ovarian cancer. From the merged analysis^17^ of 3 Gynecologic Oncology Group studies, residual tumor in the peritoneal cavity is the most important prognostic factor for progression-free survival and overall survival in patients with stage IV ovarian cancer. Disease control within the intraperitoneal disease is consistently important in stage IV ovarian cancer, as identified from the recent investigations. Therefore, intraperitoneal disease control with cytoreductive surgery and locoregional treatment, including HIPEC, is important to improve survival outcome. In addition, regional hyperthermia might induce activation of systemic immune response by activating heat shock protein, which is a potent immune modulator that stimulates innate and adaptive immune responses.^18^ Hyperthermic intraperitoneal chemotherapy can increase systemic antitumor response by the maturation of dendritic cells via heat shock protein. With these backgrounds, locoregional disease control in the peritoneal cavity, including radical surgery and HIPEC, is an important and reasonable treatment strategy in stage IV ovarian cancer.

This finding engenders the question of why HIPEC is effective only after recent exposure of chemotherapy and not in chemotherapy-naive women with ovarian cancer. From the first randomized trial of HIPEC for recurrent ovarian cancer, there was an observable profound survival benefit of HIPEC after recent exposure to chemotherapy and survival improvement in platinum-resistant recurrent disease (HR, 0.4 for overall survival; estimated from the 1-year survival curve).^19^ A retrospective study^20^ showed that, in primary ovarian cancer, the effect of HIPEC after adjuvant chemotherapy is maximized (HR, 0.2–0.4 for overall survival). In platinum-sensitive recurrent ovarian cancer, surgery with carboplatin HIPEC was well tolerated but not superior to the surgery in the Memorial Sloan Kettering team ovary phase 2 study (progression-free survival, 12.3 vs 15.7 months; *P* = .05).^21^ There could be several explanations for the consistent findings, including the effect of HIPEC only after recent exposure of chemotherapy. First, even normal-appearing peritoneum during interval cytoreductive surgery after neoadjuvant chemotherapy could have microscopic residual tumor cells, thereby potentially harboring a subpopulation of cancer cells, including chemotherapy-resistant cancer stem cells.^22,23^ After neoadjuvant chemotherapy, condensed and subpopulated chemotherapy-resistant cancer stem cells within the effective penetrating depth of cisplatin by HIPEC provide an actionable space wherein the therapeutic effect of HIPEC is demonstrable.^24^ Second, the chemotherapy-sensitive subgroup could be selected for neoadjuvant chemotherapy. Women with *BRCA* mutations respond well to neoadjuvant chemotherapy, and the *BRCA* mutation is a good predictive biomarker for better survival outcomes after intraperitoneal chemotherapy.^25,26,27^ In a randomized trial of intraperitoneal chemotherapy for ovarian cancer, the survival benefit was observed only in women with aberrant BRCA1 (OMIM 113705) expression.^4,27^ Third, hyperthermia induces degradation of *BRCA2* (OMIM 600185) and inhibits homologous recombination.^28^ Therefore, hyperthermia could induce a subpopulated chemotherapy-resistant cancer cell line in the peritoneum, whereby a homologous recombination-proficient tumor changes into a homologous recombination-deficient tumor. Fourth, after neoadjuvant chemotherapy, quiescent cells within the hypoxic and nutrient-deprived tumor regions could be targeted by hyperthermia through the repositioning of the cell cycle and reoxygenation of the hypoxic tumor, thereby triggering the immune system through the production of heat shock proteins and improving the microenvironment that is symbiotically related to the cancer stem cell.^18^ In the current trial, 9 patients with pathological complete remission on intraoperative frozen histopathological examination (Figure 1) were excluded, and their inclusion may have indicated maximization of the treatment benefit of HIPEC.

In a previous randomized trial^4^ of intraperitoneal chemotherapy, left colon surgery, including low anterior resection, was a predictive factor for the likelihood of noncompletion of the planned intraperitoneal chemotherapy. In this trial, HIPEC was safely administered after anastomosis during low anterior resection in approximately two-thirds of the women. A colostomy or ileostomy after bowel surgery was more frequently performed in the HIPEC group (72% vs 43%, *P* = .04) of OVHIPEC-1. However, in the current trial, ileostomy after bowel surgery was less frequently and similarly performed in both the groups, without intergroup differences in the anastomosis leakage.

Most toxic effects, including anemia and neuropathy, in this trial were related to the surgery and adjuvant chemotherapy. Acute kidney toxic effects and electrolyte imbalance were observed more frequently in the HIPEC group because of the absence of sodium thiosulfate use in most patients (n = 71). With the use of sodium thiosulfate in 21 patients, the incidence of these adverse events decreased significantly, without any intergroup differences between the HIPEC and control groups (eTable 4 in Supplement 2).

### Limitations

This study has several limitations, including small sample size. The enrollment into the trial was not stratified by the primary or interval cytoreductive surgery, stage, and *BRCA* status or homologous recombination deficiency. Although there is no statistical difference, the current treatment outcomes still need to be carefully interpreted because of the potential imbalance between 2 groups in terms of stage and type of primary treatment. An advantage of this trial is that the results provide preliminary clues to ascertain a possible benefit of HIPEC based on the primary treatment of primary advanced epithelial ovarian cancer.

## Conclusions

The addition of HIPEC after interval cytoreductive surgery following neoadjuvant chemotherapy was observed to reduce recurrence and mortality rates in women with primary stage III or IV epithelial ovarian cancer. Hyperthermic intraperitoneal chemotherapy could be performed safely after maximal cytoreductive surgery, including left colon surgery, without any delay in the initiation of adjuvant chemotherapy. The survival benefit of HIPEC immediately after primary cytoreductive surgery has not been identified in this trial and needs to be further investigated in future clinical trials.

## Notes

Supplement 1.
Trial Protocol

Supplement 2. **eTable 1.** FIGO Staging Classification of Ovarian Cancer **eTable 2.** Peritoneal Carcinomatosis Index (PCI) **eTable 3.** Additional Baseline Patients’ Characteristics and Surgical and Post-surgical Outcomes **eTable 4.** Comparison of Adverse Events Before and After Use of Amifostine **eTable 5.** Univariable and Multivariable Cox Proportional Hazard Regression Models for PFS and OS **eFigure 1.** Study Population of GOG 172, OVHIPEC-1, and This Trial **eFigure 2.** Subgroup Analysis of Progression-Free Survival **eFigure 3.** Subgroup Analysis of Progression-Free Survival in Patients With Primary Cytoreductive Surgery **eFigure 4.** Subgroup Analysis of Progression-Free Survival in Patients With Interval Cytoreductive Surgery After Neoadjuvant Chemotherapy

Supplement 3.
Nonauthor Collaborators

Supplement 4.
Data Sharing Statement
