# Supplementary material for: Survival After Hyperthermic Intraperitoneal Chemotherapy and Primary or Interval Cytoreductive Surgery in Ovarian Cancer: A Randomized Clinical Trial
Source: JAMA Surg. 2022 Mar 9;157(5):374–83. doi: 10.1001/jamasurg.2022.0143 (PMC8908225; doi:10.1001/jamasurg.2022.0143)
Supplement: Supplement 1. — Trial Protocol [file jamasurg-e220143-s001.pdf]

Protocol and statistical analysis plan

Hyperthermic Intraperitoneal Chemotherapy for Ovarian Cancer

Contents

1. Original protocol..2.
2. Final protocol...30.
3. Summary of the protocol changes..74.
4. Statistical analysis plan...82.

국립암센터 NATIONAL CANCER CENTER

Protocol No. :

연구제목: 난소암 환자의 수술 중 복강내온열항암화학요법 후 정맥 항암화학요법에 대한 2상 연구
(The Phase II Study of Intraoperative Hyperthermic Intraperitoneal Chemotherapy Followed by Intravenous Chemotherapy in Patients with Ovarian Cancer)

Protocol Number:

연구 책임자: 자궁암 센터 박상윤

공동 연구자: 자궁암 센터 강석범
자궁암 센터 서상수
자궁암 센터 정대철
자궁암 센터 임명철
자궁암 센터 박세현
자궁암 센터 정수연
암관리 연구과 윤영호
암통계 연구과 박소희

Approved Date:

Revised Date:

NATIONAL CANCER CENTER
323 Ilsan-ro, Ilsandong-gu,Goyang-si,
Gyeonggi-do, 410-769, Republic of Korea
TEL ;+82-31-920-2381
FAX ;+82-31-920-1238

국립암센터
NATIONAL CANCER CENTER

Protocol No. :

목 차

Protocol Abstract

I. .연구 개요 및 추진체계 10

II. 연구 배경 12

III. 연구 목적 15

IV. 임상 시험 방법 및 치료 방법 15

V. 대상자 수 및 피험자의 선정 19

VI. 치료효과를 판정하기 위한 기준 21

VII. 연구도중 제외되는 기준 23

VIII. 통계 23

IX. 피험자의 보호 25

X. 참고문헌 26

Appendix A: 증례 기록서 (Case Report Form)

Appendix B: 동의서 (Informed Consent)

19

약어	정의
CT	Computed tomography
Hb	Hemoglobin
Hct	Hematocrit
Plt	Platelet
WBC	White blood cell
RBC	Red blood cell
Cr	Creatinine
SGPT	Serum Glutamic-pyrubic transaminase
SGOT	Serum Glutamic-oxaloacetic transaminase
GOG	Gynecologic Oncology Group
CA 125	Cancer antigen 125
CA 19-9	Cancer antigen 19-9
CEA	Carcinoembryonic antigen
LFT	Liver function test
RFT	Renal function test
CBC	Complete blood count
MRI	Magnetic resonance imaging
PET	Positron emission tomography
HIPEC	Hyperthermic intraperitoneal chemotherapy
CTCAE	Common Terminology Criteria for Adverse Events
EORTC QOL-C30	European Organization for Research and Treatment of Cancer Quality of Life Questionaire - Core 30
EORTC QOL-OV28	European Organization for Research and Treatment of Cancer Quality of Life Questionaire - Ovary 28
MDASI	MD Anderson Symptom Inventory
RECIST	Response Evaluation Criteria Solid Tumors

20

국립암센터
NATIONAL CANCER CENTER

Protocol No. :

Protocol Abstract

Title: 난소암 환자의 수술 중 복강내온열항암화학요법 후 정맥 항암화학요법에 대한 2상 연구 (The Phase II Study of Intraoperative Hyperthermic Intraperitoneal Chemotherapy Followed by Intravenous Chemotherapy in Patients with Ovarian Cancer

Principal investigator: 박상윤(자궁암센터)

Co-investigator: 강석범(자궁암센터), 서상수(자궁암센터), 정대철(자궁암센터), 임명철(자궁암센터), 박세현(자궁암센터), 정수연(자궁암센터), 윤영호 (암관리 연구과), 박소희 (암통계연구과)

Objectives :

Primary objectives:

난소암 환자에서 수술 중 복강내온열항암화학요법 및 수술 후 정맥 항암화학요법의 2년 무병 생존율을 관찰한다.

Secondary objectives :

i) 난소암 환자들에서 상기 시술을 시행한 후 합병증과 3년 생존율을 관찰한다.

ii) 난소암 환자들에서 시행한 상기 시술과 연관한 삶의 질을 분석한다.

Rationale :

난소암, 난관암, 원치성 복막암 (이하 난소암)의 치료는 항암제 치료의 효과를 어떻게 극대화하느냐가 관건이라 사료되며 이의 핵심은 최대한의 종양감축술과 항암화학제의 투여방법 및 투여시기이다. 난소암은 효과적인 조기 검진법이 없으며, 조기에 발견이 될 수 있는 특이증상도 없어서, 진단시 2/3에서 복강내 전이가 되어있는 3기 이상 상태에서 발견되고 있다. 그러나, 대개의 경우 진단시 복강내에 국한되어 있으며, 재발시에도 대개 복강내에 국한되어 있다. 그래서, 난소암의 항암화학요법은 복강내에 제한하여 시행하는 것이 전신적인 부작용을 줄이고, 위치 특이적 항암화학제의 투여로 항암화학제의 치료 효과를 높일 수 있을 것이라는 생각하에 복강내 항암화학제 투여가 시도되었고, 1996년; 2001년, 2006년에 걸친 3개의 대규모 전향적 무작위 연구를 통해 수술 후 복강내 항암화학요법의 시행이 정맥 항암화학제 투여보다 통계적으로 유의하게 무병생존율과 생존율 향상을 보임이 밝혀졌다. 가장 최근 연구인 GOG #172 연구에서 복강내 항암화학요법군의 정중 생존율이 65.6개월, 대조군인 정맥 항암화학요법군의 정중 생존 기간이 49.7개월로 복강내 항암화학요법의 생존율을 향상을 보고되었다. 이에, 2006년 1월 NCI clinical announcement에서 난소암의 수술 후 표준항암화학요법으로 복강내 투여가 정맥투여보다 생존율의 향상을 보이므로 표준치료로 받아들여져야 한다고 하였다.

그러나, 복강내 항암화학요법군에서 항암화학제에 의한 여러 독성이 증가하였고, 도관에 의한 여러 합병증 및 치료 후 1년까지 삶의 질이 저하되는 등의 문제로 논란이 지속되고 있는 실정이다. 이렇게 복강내 항암화학요법이 초기 중단율이 높다고 하더라도 생존율의 향상을 보인 것은 복강내 유착발생이 미미한 시기인 수술 후 초기 복강내 약제투여에 의한 것으로

4

21

해석되고 있으므로, 이론적으로 수술 후 가장 이른 시기인 수술 중 복강내온열항암화학요법을 시행하면 도관삽입에 의한 여러 합병증 및 복부에 도관유지로 인한 삶의 질 저하를 방지하며, 더 좋은 치료효과를 기대할 수 있을 것이다.

복강내온열항암화학요법(HIPEC)은 이미 여러 연구자들에 의해서 가점액성 복막암, 복막중피종에서 시행되고 있으며, 복막파종을 동반한 대장암에서 전향적무작위연구가 시행되어 좋은 성적을 보고하고 있다. Panteix G 등은 진행된 난소암 환자에게 cisplatin을 이용하여 복강내온열항암화학요법을 시행하면서 pharmacokinetics에 대한 연구를 시행하여 복강 내 투여된 약제의 약 65%가 체내 흡수되었고, 이중 약 45%가 종양조직에 흡수되고, 약 20%만 전신흡수됨을 보고하였다. 따라서, 치료 목표조직에 항암화학제의 축적을 통해 효과를 최대화하고, 전신 부작용을 최소화할 수 있는 좋은 치료라 할 수 있다. 이후 난소암, 난관암 및 원발성 복막암에 대한 종양감축술 후 수술 중 복강내온열항암화학요법은 세계적으로 여러 보고자들 (독일의 Hager ED, Chatzigeorgiou K, 이탈리아의 Piso P, Zanon C, Raspagliesi F 아르헨티나의 Gori J)이 연구성과를 보고하였지만, 대부분 후향적 연구로 연구설계에 한계가 있는 실정이다. 수술 중 복강내온열항암화학요법의 환자 및 의료인에 대한 안전성이 여러 연구에서 확인되었고, 밀란의 NCI에서 난소암을 포함한 복막내 암 환자 209명에 대한 복강내온열항암화학요법에 대한 전향적 2상 임상연구를 시행하여 cancer지에 보고하였는데, 주요 유병율이 12%였고, 사망률이 0.9%으로 보고 되었지만, 난소암에 대한 전향적 연구가 필요한 실정이다. HIPEC 합병증은 문합부위 누공이나 천공(23명), 복강내 출혈(4명), 패혈증(4명)이 있었다.

국립암센터 자궁암센터에서 가점액성 복막암과, 복막 중피종에 대하여 2001년도부터 현재까지 10례를 HIPEC으로 치료하였고, 2006년도부터 재발성 난소암 6례를 HIPEC을 시행하였고, 장문합부위 누공이나 누출, 종양형성 등과 같은 주요 합병증 없이 안전하게 시술할 수 있음을 2006년도 복막암 연구회와 2006년도 산부인과 학회에 보고한 바 있으며, 2008년 6월까지 재발성 난소암 환자 12명에게 HIPEC을 시행하였다. HIPEC시행시 온도와 관련된 안전성 문제가 제일 중요한 것으로 지적되고 있다. 따라서 세계 각국에서 사용되고, 2009년 KFDA승인받은 HIPEC기기를 이용하여, 엄격한 안전 protocol에 대한 사전 교육과 HIPEC시행 시 protocol에 대한 철저한 기록으로 안전한 HIPEC시행에 만전을 기하였다.

난소암에서의 HIPEC이 환자의 삶의 질 향상 및 생존율에 도움이 되는지에 대한 전향적인 연구를 수행하여 난소암 치료에 있어서 종양감축술 개발뿐만 아니라 신개념의 치료법을 연구하고 임상에 도입할 수 있을 것으로 기대된다. 현재 세계적으로 일부 선두적인 기관에서 임상연구를 준비 중에 있는데, 협력과 선의의 경쟁을 통해서 통합적인 좋은 결과를 도출할 것으로 기대한다. 따라서 본 센터에서도 재발성 난소암 환자 중 적절한 최대종양감축술이 가능했던 환자에 대하여 종양감축술 후 수술 중 복강내고온항암화학요법을 시행하여, 일차적으로 수술 중 복강내고온항암화학요법 및 수술 후 정맥 항암화학요법 후 2년 무병 생존율 및 3년 생존율 및 안전성, 합병증과 삶의 질을 평가하고자 한다.

Eligibility :

. 연구대상자의 선정 >

22

Inclusion Criteria:

피험자들은 다음의 조건을 모두 만족해야 한다.

(1) 질환의 상태에 따른 조건

A. 일차성 난소암 - 난소암, 난관암, 일차성 복막암 등으로 확인된 환자 (3기 이상)

B. 재발성 난소암 - 재발성 난소암, 난관암, 일차성 복막암 환자로 일차암으로 치료 후 무병생존기간이 6개월 이상인 환자

(2) 종양감축술 후 잔류종양의 최대 직경이 1cm 미만인 경우

(2) 연령이 75세 이하인 환자

(3) 임상적 판단으로 환자의 예상수명이 최소한 3개월 이상인 경우

(4) Performance status 가 ECOG 0-1인 환자

(5) 적절한 골수기능을 가진 경우

혈색소 ≥8 g/dl(단순한 철결핍성 빈혈의 경우는 교정 후)

백혈구 ≥ 3,000/mm3,

혈소판 ≥ 100,000/mm3

(6) 적절한 신기능을 가진 경우

크레아티닌 ≤ 1.5 mg/dl

(7) 적절한 간기능을 가진 경우

빌리루빈 ≤ 1.5 mg/dl이고, 간효소치(AST/ALT) ≤ 80 IU/L

(8) 심장, 폐 등에 현저한 기능장애가 없는 경우

수술 전 폐기능 검사상 이상을 보이거나 심전도와 흉부 방사선 촬영상 이상을 보인 경우, 그리고 이전에 심장 및 폐질환이 있었던 경우에는 순환기 및 호흡기내과와의 협진으로 기능장애로 인한 수술관련 위험성이 크지 않다고 판단되는 경우

(9) 수술 전에 본 임상시험에 자의로 참여를 결정하고 환자동의서(written informed consent)에 서명한 경우

Exclusion Criteria:

다음 조건 중 1가지 이상에 해당되는 환자는 본 임상시험에 참여할 수 없다.

(1) 복강을 벗어난, 즉 뇌, 골, 폐, 경추늑골근 림프절, 쇄골하 림프절 등에 전이가 있는 경우 (단, 국소 전이로 완전 절제가 가능한 경우는 제외)

(2) 수술 중 적절한 종양감축술이 불가능했던 환자 (잔류종양 1cm 이상)

(3) 여성생식기암 이외의 다른 암을 앓은 적이 있는 환자(단, 피부암으로 근치적 절제를 받은 환자 제외)

(4) 심각한 심질환 및 심부전이 있는 경우

(5) 심각한 폐질환 및 폐 기능부전이 있는 경우

(6) 비경구 항생제 투여를 요하는 활동성 세균 감염증이 있는 환자

(7) 기타 심각한 내과적 질환이 동반된 환자

(8) 정신질환이 있는 경우

(9) 법적으로 임상시험에 참가가 불가능한 환자

(10) 임신 중이거나 수유 중인 환자

(11) 기타 의사의 판단으로 임상시험에 참가가 불가능한 환자

Treatment Plan :

A. 피험자의 선정 및 등록

임상시험의 대상 환자는 수술 전에 연구자나 연구원에 의해 등록되어야 한다.

B. 수술 전 평가

피험자의 선징과 기초적인 상태를 파악하기 위해 치료 전 검사를 실시한다. 치료 전 검사를 통해 선정기준 및 제외기준에 적합한 피험자인지 검토하여 유의한 이상이 있는 대상자는 제외한다.

C. 문서 동의

임상시험에 피험자로 참가할 수 있다고 판단되는 환자에게 본 임상 시험에 대한 구술 설명과 더불어 설명서를 제시하고 임상 시험 참여 여부에 대한 문서상 동의를 받는다. 임상시험에 참여할 것을 동의한 환자에게 수술 및 마취에 대하여 수술 전 문서상 동의를 받는다.

D. 수술 중 적절한 종양 감축 시행시 연구 참여 후 치료

본 연구에 참여하기로 동의한 경우에는 수술 중 적절한 종양 감축술이 이루어졌다고 판단될 경우에는 본 연구에 참여하게 되어 아래 E. 항 이후의 치료를 받게 된다.

E. HIPEC의 시행

복강을 폐쇄한 후 식염수을 복강내로 순환시킨다. 관류액은 41.5 ℃ 로 주입하며, cisplatin (75 mg/m^2)을 90분간 관류시킨다. 관류액의 체내 주입온도는 기계적 및 인적 감시 (손으로 직접 온도를 감지)하여 온도관찰 기록지에 표기하며, 온도 이상소견 시 정해진 protocol에 따라서 대응한다(상세 protocol 계획서 내 첨부).

F. 보조항암화학요법

수술 후 식이가 가능하고, 활동성 감염 등의 징후 없는 상태로 항암화학요법이 가능하다고 판단되면 질환의 상태에 따라서 paclitaxel & carboplatin / topotecan / docetaxel 정맥 항암화학요법 3주 간격으로 총 6회 투여한다. 독성 등으로 약제를 중단하거나, 재발하는 등의 항암화학요법을 변경해야 하는 경우에는 국립암센터 자궁암센터 지침에 의한다.

G. 치료 후 추적 검사

1)항암화학요법 3주기, 6주기에 신체검사, CA-125, CA19-9, CEA 중 임상적으로 의미있는 종양표식자 검사, 영상검사를 시행한다. 항암화학요법 시행 후 3년까지는 3개월 간격으로, 이후 2년간 6개월 간격으로, 이후 3년간 1년 간격으로 시행한다.

2) 수술 전, 수술 후 7일 후, 항암화학요법 3주기, 6주기 마나, 항암화학요법 후 1년까지는 3개월 간격으로, 이후 매년 환자의 삶의 질을 평가한다.

국립암센터
NATIONAL CANCER CENTER

Protocol No. :

24

Estimated Accrual :

1)시험기간; IRB 승인일 ~ 2017년 12월 31일

Accrual period: 3 years, Minimum Follow up period: 5 years (Total Period: 8 years)

2)총 대상자 수 214명 (일차성 난소암 168명, 재발성 난소암 46명)

일차성 난소암: 대조군에서의 중앙생존율을 18개월로 가정하고 Hazard ratio를 1.6으로 가정하였을 때(1종오류=0.05, 2-sided test, 통계적 검정력=80%) 필요한 환자수는 각 군당 79명이며, 5%의 drop out rate를 고려하여 총 84*2 = 168명이 된다.

재발성 난소암: 본 연구는 single-arm 2상 연구로 아직 복강내온열항암화학요법 후 정맥 항암화학요법을 받은 환자에서의 2년 무병생존율에 대하여 뚜렷하게 알려진 정보는 없으나, 기존 국립암센터의 자료에 따르면 중앙 무병생존율이 약 13개월인 것을 고려하여 매년 약 16명의 재발성 난소암 환자를 3년 동안 등재할 경우 총 48명의 환자가 본 연구에 포함될 예정이다.

Patient Evaluation : (Pretreatment and Interim Testing)

A. 기초검사

CBC diff. and platelet, LFT, RFT, serum electrolyte, serum calcium and magnesium, EKG, chest x-ray

B. 진단 및 질병의 범위를 정하기 위한 검사

개복수술이나 복강경수술을 통한 조직검사

복수나 흉수의 세포검사

CT and/or MRI and/or PET-CT, tumor marker(CA-125, CA 19-9, CEA)중에서 선택한다.

Statistical Consideration:

✦ 생존기간은 처음 질병이 진단된 시점으로부터 최종 시점까지의 기간을 개월수로 나타내고, 무병 생존기간은 치료 종료 후 완전 관해가 온 시점에서 질병의 증거가 다시 나타날 때까지의 기간을 개월수로 나타낸다. 생존확률은 Kaplan-Meier method를 이용하여 추정하고, 군간 생존률 비교는 logrank test를 이용한다. 임상적으로 중요한 다른 변수들과 생존기간과의 연관성을 보기 위해서는 Cox proportional hazards model, 또는 exponential model 등을 이용하고, 효과크기로는 Hazard ratio를 이용한다. 합병증의 유무는 빈도수로 나타내고 필요한 경우 Fisher' s exact test를 사용하여 빈도 분포의 차이를 검정한다. 통계적 유의성은 $p <$ 0.05인 경우로 한다.

삶의 질의 평가는 EORTC QOL-C30, EORTC QOL-OV28, MDASI를 이용하고 3분류로 평가(1.Functional scale 2.Global quality of life 3.Symptom scale)한다. 각 분류마다, 매 평가할 때 마다 raw answer에 대한 frequency와 평균치 분석, 반복적 측정으로 인한 시간의 변화에 따른 삶의 질 변화 평가를 시행한다. 통계학적 분석은 ANCOVA (Analysis of Covariance), ANOVA for repeated measures, Mixed Effects model, GEE (Generalized Estimating Equation)등을 이용한다.

8

25 Protocol No. :

Site of Study :		
This protocol is performed as an :		
☐Inpatient	☐Outpatient	☒Both
Where will study be conducted :		
☒Only at NCC	☐NCC + Community Program	☐Independent Multicenter Arrangements
Name of Research Nurse / Data Manager Responsible for Protocol :		
정수연		
Submit Protocol to Clinical Research Center Review Committee :		
☒Yes	☐No	

Ⅰ. 연구 개요 및 추진 체계

[일차성 상피성 난소암]

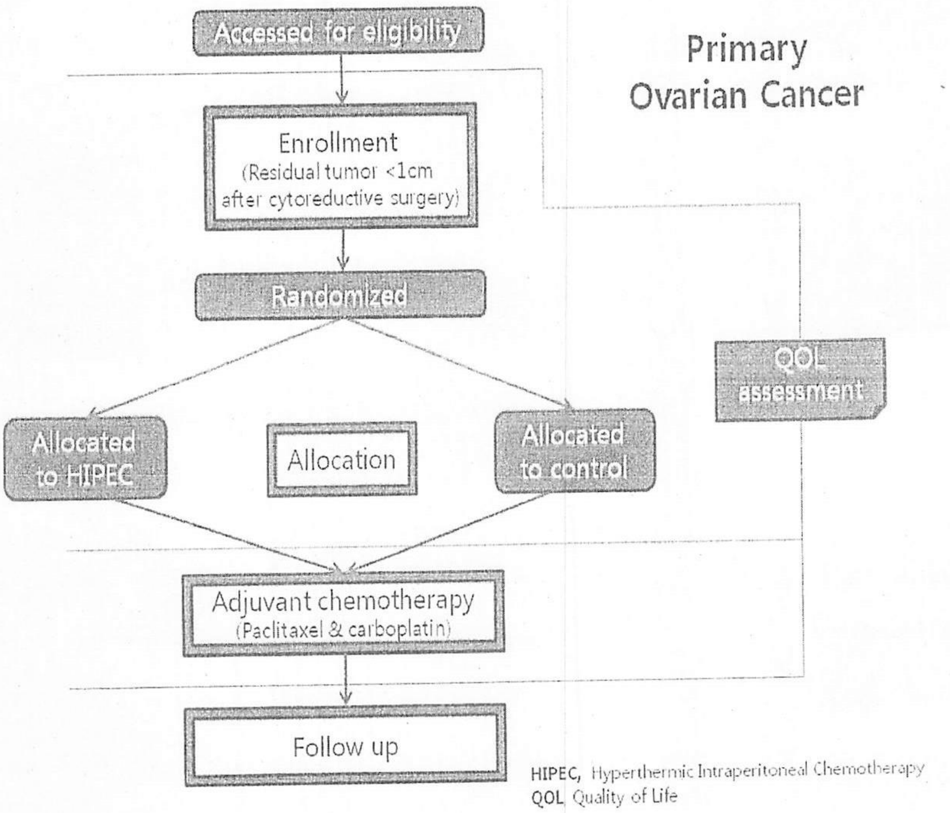

HIPEC, Hyperthermic Intraperitoneal Chemotherapy
QOL, Quality of Life

[재발성 상피성 난소암]

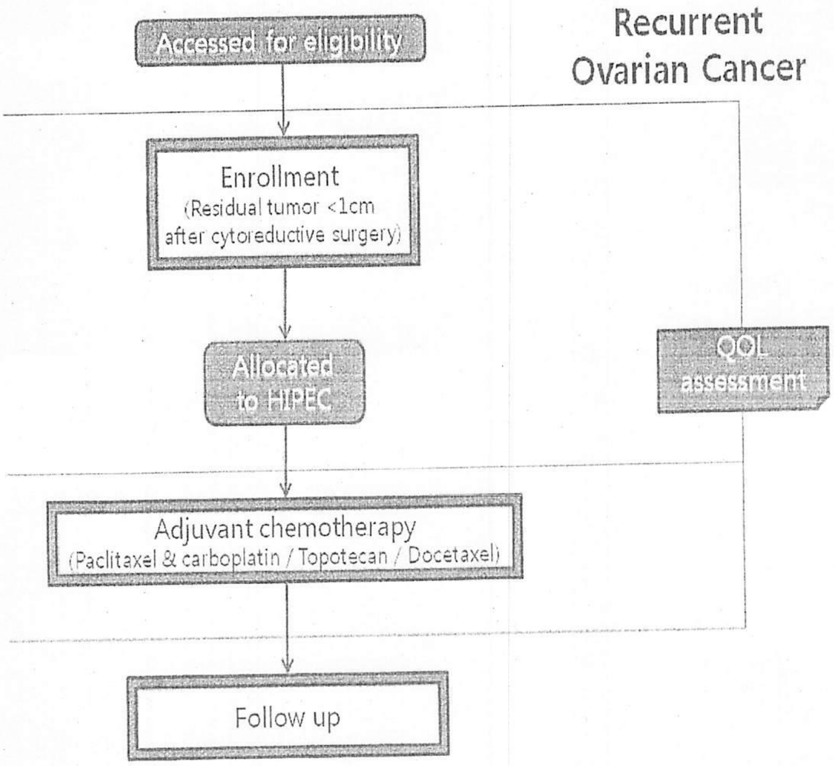

HIPEC, Hyperthermic Intraperitoneal Chemotherapy
QOL Quality of Life

Ⅱ. 연구의 배경

A. 난소암에서 시행한 수술중 복강내온열항암화학요법에 대한 국내외 연구 및 기술현황

1. 외국 현황

Panteix G 등은 난소암 환자 16명에 대한 phase I 연구 (cisplatin 60mg, 80mg, 100mg)를 시행하였다. HIPEC 시행 후 평균 65%가 체내로 흡수되는데, 최대 혈장 농도를 보이는 시간은 투여 후 1~1.5시간 뒤였고, 제거 반감기는 80시간이었다. 투여된 cisplatin의 20%만 소변으로 배출되고, 복강내 투여된 cisplatin의 45%는 종양조직에 흡수되므로 전신 부작용은 줄이고, 치료 효과를 최대화 할 수 있는 좋은 치료(진행된 난소암에서 3년 생존율: 37.5%, 7년 생존율: 12.5%)라 할 수 있다.

난소암, 난관암 및 원발성 복막암에 대한 종양감축술 후 수술 중 복강내 온열항암화학요법은 세계적으로 여러 보고자들 (독일의 Hager ED, Chatzigeorgiou K, 이탈리아의 Piso P, Zanon C, Raspagliesi F 아르헨티나의 Gori J)이 연구성과를 보고하였다. 수술 중 복강내온열항암화학요법 시행시 이중 latex glove착용으로 의료인에게 chemotherapeutic agent가 피부로 흡수되는 것이 방지되는 것을 확인하였고 (Schmid K, Eur J Surg Oncol, 2006), 의료인 및 환자에게 수술 중 복강내온열항암화학요법이 안전하게 시행될 수 있음이 보고되었다 (Gonzalez-Bayon L, Eur J Surg Oncol, 2006). 또한, 밀란의 NCI에서 난소암을 포함한 복강 내 암환자 209명에 대한 수술 중 복강내온열항암화학요법에 대한 전향적 2상 임상연구를 시행하여 cancer지에 보고하였는데, 주요 유병율이 12%였고, 사망률이 0.9%였다. 주요 합병증은 문합부위 누공이나 천공(23명), 복강내 출혈(4명), 폐혈증(4명)이 있었다. 난소암에서 시행된 대부분의 연구에서 알 수 있듯이 수술 전 여러 번의 항암화학요법 후에도 재발한 난소암 환자에게 안전하게 시행되었으므로, 항암화학제에 노출이 되지 않은 환자에게 시행된다면 보다 안전하고 보다 좋은 성적을 보일 것으로 기대된다.

2. 국내 현황

가) 난소암, 난관암 및 원발성 복막암의 수술적 치료 및 수술 중 복강내온열항암화학요법에 대하여 본 연구자 팀(국립암센터 자궁암센터) 외에는

카톨릭대학교 이준모 팀이 연구와 치료를 시도하고 있으며, 일부 성과를 보고하였지만, 후향적 연구로 다양한 환자군을 포함하고 있어서, safety와 feasibility확인 외에 표준 진료 지침으로 제시되기에는 한계가 있는 것으로 지적되고 있다.

나) 본 연구자 팀(국립암센터 자궁암센터)은 2001년부터 현재까지 복막 위점액종(난소기원-4례, 일차성 복막암 기원-1례, 충수돌기기원 3례) 및 복막 중피종(2례), 재발성 난소암(6례) 총 16례에 대하여 종양감축술 및 수술 중 복강내온열항암화학요법을 시행하였다. 수술로 인한 사망률이나, 누공, 농양형성, 백혈구 감소 등과 같은 주요 합병증은 없었고, 안전하게 시술될 수 있음을 2006년 복막암 연구회, 산부인과 학회에 보고하였다. 2008년 6월까지 재발성 난소암 환자 12명에게 HIPEC을 시행하였다. 특히, 일차성 난소암 환자에게는 세계적으로 처음으로 phase II연구를 수행하여 2009 Annals of Surgical Oncology에 HIPEC의 안전성을 보고하였다.

B. 난소암 임상 양상과 연구의 필요성

난소암 치료는 항암제 치료의 효과를 어떻게 극대화하느냐가 관건이라 사료되며 이의 핵심은 최대한의 종양감출술을 시행하여 최대잔여종양의 직경을 최소화하는 것과 수술 후 항암화학제 투여 경로와 투여시기의 설정에 달려 있다고 할 수 있다. 난소암은 복강 내에 국한되어 발생하고, 재발하더라도 대개 복강 내에 국한되어 있다. 그래서 복강내 항암화학요법을 시행하면, 전신투여 보다 복강내 치료효과는 높이고 전신적 부작용은 감소시킬 수 있을 것이라 생각되고 있으며, 이미 여러 연구들을 통하여 밝혀졌다. 2006 년 1 월 NCI clinical announcement 를 통해 난소암 치료의 수술 후 표준 항암화학요법은 복강내 항암화학요법으로 바뀌어 가고 있는 추세이다.

앞으로 난소암에서 수술 후 항암화학요법의 효과를 최대화 하기위해서 항암화학요법의 투여시기, 투여효과를 최대화하여야 생존율의 향상을 기대할 수 있을 것이다. 이러한 방법은 현재 복막 위점액종, 복막 중피종에 적용되고 있는 수술 중 복강내 온열 항암 화학 요법이라 할 수 있다. 수술 중 항암화학요법을 복강내 시행함으로 수술 후 발생하는 유착에 의한 항암화학제의 고른 분포의 저해가 없으며, 종양감축술 시 탈락된 암세포를 세척하고, 항암화학제로 살상할 수 있어서 상처부위로 부착 및 성장을 원천적으로 차단 할 수 있는 큰 장점이 있다. 또한 온열은 온열 자체의 세포독성 이외에 항암제의 세포독성을 향상시킨다. 이는 약물의 세포막 투과성 증가, 세포막 회복의 감소, 온열에 따른 약의 대사항진 등이 작용하기 때문으로 설명되고 있다. '수술 중 복강 내 온열 항암제치료(HIPEC)' 은 1980 년대

이후 복막가점액종, 위암, 대장암, 재발성 난소암 등에 보고되고 있었으며, 이탈리아, 독일 등의 국가에서 이미 난소암, 일차성 복막암에 대한 일차적 치료로 1990 년대 후반부터 점차 1도되고 있으며, 그 안정성 및 효용성이 보고되고 있는 실정이다. 이에 국립암센터 자궁암센터는 본 연구에서 난소암, 난관암, 일차성 복막암에 대하여 적절한 종양감축술이 시행된 경우 HIPEC 을 시행하고, 수술 후 식이가 가능하고, 활동성 감염 등의 징후 없는 상태로 항암화학요법이 가능하다고 판단되면 paclitaxel, carboplatin 으로 정맥 항암화학요법을 시행하여 총 6 차례의 항암화학요법을 시행하고자 한다.

현재, 이러한 치료효과를 확인하기 위한 연구들이 계획되고 있다. 아직 현재까지 난소암에서의 종양감축술 후 수술 중 복강내항암화학요법이 생존율과 삶의 질 향상에 도움이 되는지에 대한 전향적인 연구는 아직 보고가 없는 실정으로, 이에 대한 연구 및 검토가 시급한 실정이다.

1) 일차성 난소암에서 HIPEC 연구의 필요성

이미 복강내 항암화학요법이 정맥항암화학요법보다 생존율을 증가시킴이 미국 부인종양학회의 3 개의 대규모 Randomized control study 로 입증되었고, 2006 년 복강내 항암화학요법이 선호되는 치료법으로 사용되어야 한다고 NCI clinical announcement 가 있었지만, 독성과 삶의 질 감소로 임상적용에 한계가 있었던 것이 분명하다. 이론적으로 복강내 항암화학요법의 장점 (유착이 없는 수술직후 복강내 직접 투여, 온열로 항암화학요법의 효과 증대 등)을 극대화하고 단점(도관으로 인한 복통 및 감염 등)을 최소화할 수 있을 것으로 제시되고 있다. 또한 2009 년 국립암센터에서 세계 최초로 Annals of surgical oncology 지에 일차성 난소암에 대한 phase II 연구결과 안전하고 유용하다는 점을 보고하였다. 이미 수술 후 복강 내 항암화학요법이 생존율은 유의하게 증가시키지만, 삶의 질 감소로 임상에 널리 사용되지 못하는 점을 고려할 때, HIPEC 에서 안전성 및 효과 이외에 삶의 질에 미치는 영향이 매우 중요한 평가 항목이라고 할 수 있다. 따라서, 일차성 난소암에서는 phase II RCT 를 통해서 비교연구가 필요하며, 특히 삶의 질에서의 결과를 확인하는 것이 매우 중요할 것으로 지적되고 있다.

2) 재발성 난소암에서 HIPEC 연구의 필요성

난소암은 복강 내에 이미 전이된 채로 진단되지만, 재발과 사망시에도 주로 복강내 질환으로 국한된 채로 제한된다는 점이 난소암의 고유한 특징이라고 할 수 있으며, 이점이 난소암 치료법 개발의 핵심이라고 할 수도 있다. 따라서, 재발성 난소암도 복강 내에 절제 가능한

부위에 국한되어 있다면, 최대 종양감축술을 시행하고 복강 내 종양에 대한 항암화학요법을 시행하는 것이 이론적으로 가장 좋은 치료로 제시된다. 이미 여러 후향적 연구에서 재발성 난소암에서도 안전하게 HIPEC 을 시행할 수 있음이 보고되었지만, 이 역시 전향적인 연구로 시행되어야 하며, 무엇보다 삶의 질 결과를 도출하여야 한다.

따라서 본 센터에서는 난소암 환자 중 적절한 최대종양감축술이 가능할 것으로 판단되는 환자에 대하여 종양감축술을 시행하고, 수술 중 복강내항암화학요법을 시행하여, 수술 중 복강내항암화학요법과 관련된 합병증을 분석 비교하며, 이러한 환자의 삶의 질을 전향적 연구를 통하여 평가하고자 한다.

Ⅲ. 연구 목적

Primary objectives:

난소암 환자에서 수술 중 복강내온열항암화학요법 및 수술 후 경맥 항암화학요법의 2년 무병 생존율을 관찰한다.

Secondary objectives:

i) 난소암 환자들에서 상기 시술을 시행한 후 합병증과 3년 생존율을 관찰한다.

ii) 난소암 환자들에서 시행한 상기 시술과 연관한 삶의 질을 분석한다.

IV. 임상 시험 방법 및 치료 방법

A. 피험자의 선정 및 등록

임상시험의 대상 환자는 수술 전에 연구자나 연구원에 의해 등록이 되어야 한다.

B. 수술 전 평가

피험자의 선정과 기초적인 상태를 파악하기 위해 치료 전 검사를 실시한다. 치료 전 검사를 통해 선정기준 및 제외기준에 적합한 피험자인지 검토하여 유의한 이상이 있는 대상자는 제외한다.

1)병력/약물 사용력 청취 및 신체검사(부인과 검사 포함): 신장/체중, 운동능력(ECOG) 등

2)기본적인 임상병리 검사

일반혈액검사: WBC, RBC, Hb, Hct, PLT, differential count

일반화학검사: Ca, P, glucose, uric acid, BUN, creatinine, LDH, cholesterol, protein, albumin, bilirubin, Alk. phosphatase, AST, ALT, 전해질(Na, K, Cl), Mg

혈액응고검사: PT/aPTT

혈액은행검사; ABO/Rh

혈청검사: VDRL, HBsAg, HIV Ab, HCV Ab

소변검사: 비중, pH, albumin, glucose, ketone, blood, urobilinogen, bilirubin, nitrite, microscopy

3)수술 전 환자의 기본적인 삶의 질 평가 (EORTC QOL-C30, EORTC QOL_OV28, MDASI)

4)종양표지자 검사; CA-125, CA19-9, CEA

5) Chest PA/Lat

6) 심전도

7) Pelvis CT scan 또는 MRI 및 기타 필요한 검사

8) 양전자 단층촬영(PET-CT); 선택적

C. 문서 동의

임상시험에 피험자로 참가할 수 있다고 판단되는 환자에게 본 임상 시험에 대한 구술 설명과 더불어 설명서를 제시하고 임상 시험 참여 여부에 대한 문서상 동의를 받는다. 임상시험에 참여할 것을 동의한 환자에게 수술 및 마취에 대하여 수술 진 문서상 동의를 받는다.

D. 수술 후 적절한 종양감축술이 이루어 진 경우

아래 E. 항 이후의 치료를 받게 된다.

E. 수술 중 복강내온열항암화학요법 (HIPEC)

1) Catheter & thermometer의 위치

-Inflow catheter는 양측 횡경막 밑에, outflow catheter는 골반 내에 위치시킨다.

- 복강 내 온도측정을 위한 thermometer을 횡경막 밑에, 골반강내 위치시킨다.

복강 내 관류를 위해서 피부 연속 봉합으로 폐쇄된 복강 공간을 만든다. 절개부위

중심 2-5Cm 정도는 봉합하지 않은 체 노출 시켜서 처음 관류액이 복강을 채울 때 공기가 빠질 수 있도록 하며, 관류액이 복강을 채우면, 완전 봉합한다.

2) 관류

- 관류 Belmont® Hyperthermia pump approved by KFDA 사용
- 유입되는 수액은 1분에 약 1L로 유지한다.
- 관류하는 90분 동안 환자의 측면을 흔들어서 항암화학제의 복강 내 고른 분포를 돕도록 한다.

3) 온도조절

- Nasopharyngeal-temperature probe를 이용하여 core temperature를 측정한다.
- 관류를 시작할 때 0.9% saline solution을 41.5 ℃까지 올려서 관류를 시작한다.
- 관류하는 동안 5분마다 체온을 측정하여 core temperature가 39 ℃를 넘지 않도록 한다.
- 복강 내로 유입되는 수액의 온도는 41.5 ℃로 한다.

☆Hyperthermia pump 온도 안전성

●기계적 감시

1. Temperature range: 37 ° C-46 ° C 로 HIPEC 시 안전한 범주 내에서만 가온 되도록 설계됨.
2. Continuously temperature monitoring system on display: (기계적 감시)

   True pump output temperature

   4 user controlled patient temperature (T1, T2, T3, T4)

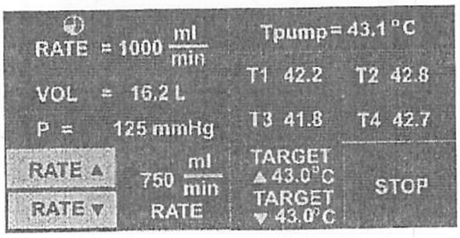

3. Safety function (over temperature 관련):

① Unsafe condition 이 발생할 경우 (heating fault or over temperature)

A. Alarm 경고음

B. 알람 매시지 표시

C. 펌프정지, 가온정지, 환자 라인 벨브 클램프(recirculation)

② 펌프 및 가온정지 조건

A. Target temperature < 39 ° C, flow rate ≥40ml/min: > 42 ° C for 20ml, 45 ° C for 0.25 seconds

B. Target temperature < 39 ° C, flow rate < 40ml/min: > 42 ° C for 10ml

C. Target temperature > 39 ° C, flow rate ≥40ml/min: > 3 ° C for 20ml

D. Target temperature > 39 ° C, flow rate < 40ml/min: > 3 ° C for 10ml 또는 6 ° C for 0.25 seconds

- 인적 감시 병행. 시술이 진행되는 동안은 담당 의료진이 5분마다 온도를 측정 하여 기록한다. 온도 측정과 별개로 체내로 투입되는 관류관과 체외로 배출되는 관류관을 손으로 직접 확인하고 이를 기록지에 기재한다.

- Core temperature가 39 ℃ 초과 시 즉시 다른 의료진들과 마취과에 연락을 취하며 다음의 조치를 취한다.

① 가온 정지

② 마취과 조치

i. Ice pack apply around head

ii. Rapid inflow of cold saline

iii. Cooling of blanket

③ 수술field내 조치

i. 개복고려 & 복강 내 직접 관류 고려

ii. Drain out 후 복강 내에 가온 되지 않은 saline 투여

4) Cisplatin 투여

- 복강 내 체온이 41.5 ℃ 이상 유지되면 관류 시 cisplatin (75 mg/m²)을 90분간 관류시킨다. 관류 전, 관류 후에 적절한 수액을 공급하고, 항 구토제를 투여한다.

F. 수술 후 항암화학요법

수술 후 식이가 가능하고, 활동성 감염 등의 징후 없는 상태로 항암화학요법이 가능하다고 판단되면 질환의 상태에 따라서 paclitaxel & carboplatin / topotecan / docetaxel 정맥 항암화학요법 3주 간격으로 총 6회 투여한다. 항암화학요법을 시작하거나, 독성 등으로 약제를 중단 혹은 감량하거나, 재발하는 등의 항암화학요법을 변경해야 하는 경우에는 국립암센터 자궁암센터 지침에 의한다.

G. 치료 후 추적 검사

1)항암화학요법 3주기, 6주기에 신체검사, CA-125, CA19-9, CEA 중 임상적으로 의미있는 종양표식자 검사, 영상검사를 시행한다. 항암화학요법 시행 후 3년까지는 3개월 간격으로, 이후 2년간 6개월 간격으로, 이후 3년간 1년 간격으로 시행한다.

2) 수술 전, 수술 후 7일 후, 항암화학요법 3주기, 6주기 마다, 항암화학요법 후 1년까지는 3개월 간격으로, 이후 매년 환자의 삶의 질을 평가한다.

V. 대상자 수 및 피험자의 선정

A. 시험 기간 및 대상자 수

B. 피험자의 선정

1)시험기간; IRB 승인일 ~ 2017년 12월 31일

Accrual period: 3 years, Minimum Follow up period: 5 years (Total Period: 8 years)

2)총 대상자 수 214명 (일차성 난소암 168명, 재발성 난소암 46명)

일차성 난소암: 대조군에서의 중앙생존율을 18개월로 가정하고 Hazard ratio를 1.6으로 가정하였을 때(1종오류=0.05, 2-sided test, 통계적 검정력=80%) 필요한 환자수는 각 군당 79명이며, 5%의 drop out rate를 고려하여 총 84*2 = 168명이 된다.

재발성 난소암: 본 연구는 single-arm 2상 연구로 아직 복강내온열항암화학요법 후 정맥 항암화학요법을 받은 환자에서의 2년 무병생존율에 대하여 뚜렷하게 알려진 정보는 없으나, 기존 국립암센터의 자료에 따르면 중앙 무병생존율이 약 13개월인 것을 고려하여 매년 약 16명의 재발성 난소암 환자를 3년 동안 등재할 경우 총 48명의 환자가 본 연구에 포함될 예정이다.

Inclusion Criteria:

피험자들은 다음의 조건을 모두 만족해야 한다.

(1) 질환의 상태에 따른 조건

A. 일차성 난소암 - 난소암, 난관암, 일차성 복막암 등으로 확인된 환자 (3기 이상)

B. 재발성 난소암 - 재발성 난소암, 난관암, 일차성 복막암 환자로 일차암으로 치료 후 무병생존기간이 6개월 이상인 환자

(2) 종양감축술 후 잔류종양의 최대 직경이 1cm 미만인 경우

(2) 연령이 75세 이하인 환자

(3) 임상적 판단으로 환자의 예상수명이 최소한 3개월 이상인 경우

(4) Performance status 가 ECOG 0-1인 환자

(5) 적절한 골수기능을 가진 경우

혈색소 ≥8 g/dl(단순한 철결핍성 빈혈의 경우는 교정 후)

백혈구 ≥ 3,000/mm3,

혈소판 ≥ 100,000/mm3

(6) 적절한 신기능을 가진 경우

크레아티닌 ≤ 1.5 mg/dl

(7) 적절한 간기능을 가진 경우

빌리루빈 ≤ 1.5 mg/dl이고, 간효소치(AST/ALT) ≤ 80 IU/L

(8) 심장, 폐 등에 현저한 기능장애가 없는 경우

수술 진 폐기능 검사상 이상을 보이거나 심전도와 흉부 방사선 촬영상 이상을 보인 경우, 그리고 이전에 심장 및 폐질환이 있었던 경우에는 순환기 및 호흡기내과와의 협진으로 기능장애로 인한 수술관련 위험성이 크지 않다고 판단되는 경우

(9) 수술 전에 본 임상시험에 자의로 참여를 결정하고 환자동의서(written informed consent)에 서명한 경우

Exclusion Criteria:

다음 조건 중 1가지 이상에 해당되는 환자는 본 임상시험에 참여할 수 없다.

(1) 복강을 벗어난, 즉 뇌, 골, 폐, 경추늑골근 림프절, 쇄골하 림프절 등에

전이가 있는 경우 (단, 국소 전이로 완전 절제가 가능한 경우는 제외)

(2) 수술 중 적절한 종양감축술이 불가능했던 환자 (잔류종양 1cm 이상)

(3) 여성생식기암 이외의 다른 암을 앓은 적이 있는 환자(단, 피부암으로 근치적 절제를 받은 환자 제외)

(4) 심각한 심질환 및 심부전이 있는 경우

(5) 심각한 폐질환 및 폐기능부전이 있는 경우

(6) 비경구 항생제 투여를 요하는 활동성 세균 감염증이 있는 환자

(7) 기타 심각한 내과적 질환이 동반된 환자

(8) 정신질환이 있는 경우

(9) 법적으로 임상시험에 참가가 불가능한 환자

(10) 임신 중이거나 수유 중인 환자

(11) 기타 의사의 판단으로 임상시험에 참가가 불가능한 환자

VI. 치료효과를 판정하기위한 기준

A. 종양감축술 및 수술 중 복강내온열항암화학요법과 관련된 parameter들의 분석

수술시간, 출혈양, 수혈 여부, 수술중의 합병증, 체온 변동, 신체 활력 징후

B. 종양감축술 및 수술 중 복강내온열항암화학요법 후의 유병률

중환자실 재원일수 그리고 수술 후 혈액검사결과, 재원일수, G/O까지 소요일, 식이진행까지의 소요일, 흉관 삽입 여부, 보조항암화학요법까지의 기간 등을 분석한다. 문합부 누출, 농양, 수술 후 출혈, 패혈증, 췌장염, 폐렴, 신경학적 합병증, 골수독성, 신독성 등의 합병증은 수술 후 30일을 기준으로 급성과 만성합병증으로 나누어 관찰하며, 이에 대해 행하여진 처치를 상세히 기록한다. 수술에 의한 사망은 30일을 기준으로 한다.

C. 임상적 반응

RECIST(Response Evaluation Criteria Solid Tumors)를 이용하여 임상적 반응을 평가한다.

D. 항암제 독성의 평가

복강 내 온열 항암화학요법으로서 cisplatin 투여 후 부작용의 평가는 Common

Terminology Criteria for Adverse Events v3.0 (CTCAE)을 이용한다. 각 항암화학요법 전에 병력의 청취 및 신체검사를 시행하고, 임상병리검사와 방사선 및 심전도 검사를 시행한다.

이상반응의 평가는 다음과 같은 계획으로 이루어 진다.

가) HIPEC 후 1일째

- 문진, 신체검사, vital sign, ECOG PS 및 24시간 뇨량
- 일반혈액검사: WBC, RBC, Hb, Hct, PLT, differential count
- 일반화학검사: BUN/creatinine, 전해질(Na, K, Cl), Mg
- Chest AP or Chest PA

나) HIPEC 후 2일째

- 문진, 신체검사, vital sign, ECOG PS 및 24시간 뇨량
- 일반혈액검사: WBC, RBC, Hb, Hct, PLT, differential count
- 일반화학검사: BUN/creatinine, 전해질(Na, K, Cl), Mg
- Chest AP or Chest PA (1일째 검사 소견 이상 시)

다) HIPEC 후 4일째

- 문진, 신체검사, vital sign, ECOG PS 및 24시간 뇨량
- 일반혈액검사: WBC, RBC, Hb, Hct, PLT, differential count
- 일반화학검사: BUN/creatinine, 전해질(Na, K, Cl), Mg
- Chest AP or Chest PA (2일째 검사 소견 이상 시)

라) HIPEC 후 7일째

- 문진, 신체검사, vital sign, ECOG PS
- 일반혈액검사: WBC, RBC, Hb, Hct, PLT, differential count
- 일반화학검사: BUN/creatinine, 전해질(Na, K, Cl), Mg
- Chest AP or Chest PA (4일째 검사 소견 이상 시)

3. 삶의 질 평가

EORTC QOL-C30, EORTC QOL-OV28, MDASI을 이용한다. 평가하는 시점은 앞의 IV항의 G.

추적검사에서 기술한 바와 같고. 통계적인 방법에 대해서는 VIII항에서 기술하는 바와 같다.

.. 생존율의 분석

대상환자들의 추적진료를 통하여 무병생존율과 생존율을 분석한다. 통계적인 방법에 대해서는 VIII항에서 기술하는 바와 같다.

VII. 연구도중 제외되는 기준

1. 환자가 원하는 경우
2. 다른 심각한 내과적 이상소견 등 중대한 이상 반응 발생 시

VIII. 통계

< 대상자수 산정 >

총 대상자 수 214명(일차성 난소암 168명, 재발성 난소암 46명)이 될 것이고, 환자 등재 기간은 3년, 마지막 환자 등재 이후 총 추적기간은 5년으로 한다.

ㄱ* 일차성 난소암: 대조군에서의 중앙생존율을 18개월로 가정하고 [N Engl J Med 2006;354:34-43] Hazard ratio를 1.6으로 가정하였을 때(1종오류=0.05, 2-sided test, 통계적 검정력=80%) 필요한 환자수는 각 군당 79명이며, 5%의 dropout rate를 고려하여 총 84*2 = 168명이 된다.

□* 재발성 난소암: 본 연구는 single-arm 2상 연구로 아직 복강내온열항암화학요법 후 정맥 항암화학요법을 받은 환자에서의 2년 무병생존율에 대하여 뚜렷하게 알려진 정보는 없으나, 기존 국립암센터의 자료에 따르면 중앙 무병생존율이 약 13개월인 것을 고려하여 매년 약 16명의 재발성 난소암 환자를 3년 동안 등재할 경우 총 48명의 환자가 본 연구에 포함될 예정이다 (alternative 중앙 무병생존율: 20개월 가정, 1종오류=5%, 2-sided test, power=80%).

< 통계 분석 방법 >

1. 증례기록 서식에 따라 기록하고 통계 처리한다. 환자들의 기본적인 특성 및 중요 변수들은 기술통계적인 방법을 이용하여 요약한다. 범주형 변수의 경우 교차표 및 카이제곱 검정 또는 Fisher' s Exact Test를 이용하고 연속변수의 경우 평균, 표준편차 또는 중앙값 등으로 요약한다.

2. 이환율과 사망률을 분석한다.

문합부 누출, 농양, 수술 후 출혈, 폐혈증, 췌장염, 폐렴, 신경학적 합병증, 골수독성, 신독성 등의 합병증은 수술 후 30일을 기준으로 급성과 만성합병증으로 나누어 관찰한다. 수술에 의한 사망은 30일을 기준으로 한다.

합병증과 수술에 의한 사망여부는 빈도수로 제시하며 임상적인 가능한 예후인자에 따른 차이를 볼 경우 Fisher' s exact test를 이용하여 분석한다.

3. 생존기간은 처음 질병이 진단된 시점으로부터 최종 시점까지의 기간을 개월수로 나타내고, 무병 생존기간은 치료 종료 후 완전 관해가 온 시점에서 질병의 증거가 다시 나타날 때까지의 기간을 개월수로 나타낸다. 생존확률 및 median survival time은 Kaplan-Meier method를 이용하여 추정하고 군간 생존률 비교는 log rank test를 이용한다. 임상적으로 중요한 다른 변수들과 생존기간과의 연관성을 보기 위해서는 Cox proportional hazards model, 또는 exponential model 등을 이용하고, 효과크기로는 Hazard ratio를 이용한다. 합병증의 유무는 빈도수로 나타내고 필요한 경우 Fisher' s exact test를 사용하여 빈도 분포의 차이를 검정한다.

4. 중요한 예후 인자를 평가한다.

생존에 영향을 미칠 것으로 보이는 인자들 즉, 환자의 연령, 난소암의 병기, 조직형, 분화도, 수술 후 잔류 종양의 크기 등을 포함하여 수술명, 수술 시간, 수술 중 합병증, 중환자실 재원일수, 수술 후 재원일수, G/O까지 소요일, 식이진행까지의 소요일, 흉관 삽입 여부, 보조항암화학요법까지의 기간, 사용된 항암화학요법의 종류, 혈전증과 같은 내과적 동반질환, 재발종양의 크기, 1차 치료 후 재발까지의 기간 등의 예후 인자로서의 가치를 Cox's proportional hazard model 또는 exponential model을 이용하여 추정한다. 통계적 유의성은 $p<0.05$인 경우로 한다.

5. 삶의 질은 국립암센터 삶의질향상연구과에서 편집한 양식을 사용하여 HIPEC 전, HIPEC 1주일 후, 항암화학요법 후 매 3주기 마다, 항암화학요법 후 1년까지는 3개월 간격으로, 이후 매년 삶의 질을 평가한다. 삶의 질은 EORTC QOL-C30, EORTC QOL-OV28, MDASI를 이용하여 평가하고, 3분류로 (1.Functional scale 2.Global quality of life 3.Symptom scale) 평가한다. 각 분류마다, 매 평가할 때 마다 raw answer에 대한 frequency와

평균치 분석, 반복적 측정으로 인한 시간의 변화에 따른 삶의 질 변화 평가를 시행한다. 통계적 분석은 repeated ANCOVA (Analysis of Covariance), ANOVA for repeated measures, Mixed Effects model, GEE (Generalized Estimating Equation)를 이용한다.

IX. 피험자의 보호

시험자는 피험자에게 본 임상시험에 대해 충분히 설명하고, 각 피험자가 본 시험에 참여 할 것인가에 대해 피험자로부터 반드시 문서 동의를 받아야 한다. 동의는 동의서 양식에 피험자의 서명과 날짜를 기입함으로써 문서화된다. 시험자가 규정에 따라 엄격히 임상시험을 실시했음에도 불구하고 피험자가 본 임상시험에서 시행된 검사 및 수술적 처치와 인과론적인 관련이 있는 합병증의 발생으로 고통을 당하게 되면 이에 대한 합리적인 의학적 조치를 취하게 된다. 그러나 그에 따른 경제적인 보상은 시행하지 않는다.

X. Reference

[illegible]. Shingleton HM, Kim RY. Treatment of cancer of the cervix, In: Gusberg SB, Female genital cancer, NY, Churchill Livingstone, 1988, 297-335.

2. DiSaia PJ, Creasman WT. Clinical Gynecologic Oncology, St. Louis, Mosby-Year Book, 1993, 111.

3. Pecorelli S, Beller U, Heintz APM, Benedett JL, Creasman WT, Pettersson F. Annual report on the results of treatment in gynecologic cancer. J Epi Biostat 2001; 6; 116.

4. McClay EF, Howell SB. A review: i.p. cisplatin in the management of patients with ovarian cancer. Gynecol Oncol 1990; 36: 1-6.

5. Albert DS, Liu PY, Hanningan EV, et al. Intraperitoneal cisplatin plus intravenous cyclophosphamide versus intravenous cisplatin plus intravenous cyclophosphamide for stage III ovarian cancer. N Eng J med 1996; 335: 1950-5.

6. Dahl O. Mechanism of thermal enhancement of chemotherapeutic cytotoxicity. In: Urano M, Douple E (eds) Hyperthermia and Oncology: Trecht 1994; 4: 9-28.

[illegible]. Vange NV, Goethem ARV, Zoetmulder FAN, Kaag MM, Vaart PJMV, Huinink WWTB, and Beijnen JH. Extensive cytoreductive surgery combined with intra-operative intraperitoneal perfusion with cisplatin under hyperthermic condition (OVHIPEC) in patients with recurrent ovarian cancer: a feasibility pilot. Eur j Sur Oncol 2000; 26: 663-668.

8. Sugarbaker PH, Kern K, Lack E. Malignant pseudomyxoma peritonei of colonic origin. Natural history and presentation of a curative approach to treatment. Dis Colon Rectum 1987; 30: 772-779.

9. Koga S, Hamazoe R, Maetra M, Shimizu N, Murakami A, Wakatsuki T. Prophylactic therapy for peritoneal recurrence of gastric cancer by continuous hyperthermic peritoneal perfusion with mitomycin C. Cancer 1988; 61: 232-7

10. Schneebaum S, Lange M, Arnold M, et al. I.P. hyperthermic perfusion for recurrent colorectal cancer, a feasibility study. Reg Cancer Treat 1992; 4: 277-281.

11. Scarabelli C, Gallo A, Carbone A: Secondary cytoreductive surgery for patients with recurrent epithelial ovarian carcinoma. Gynecol Oncol 2001;83:237-241

[illegible]2. Janicke F, Holscher M, Kuhn W, et al: Radical surgical procedure improves survival time in patients with recurrent ovarian cancer. Cancer 1992;70:2129-2136

13. Eisenkop SM, Friedman RL, Spirtos NM: The role of secondary cytoreductive surgery in the treatment of patients with recurrent epithelial ovarian carcinoma. Cancer 2000;88:144-153

14. A.R. Munkarah, R.L. Coleman: Critical evaluation of secondary cytoreduction in recurrent ovarian cancer. Gyn Oncol 2004;95:273-280

15. Armstrong DK, Bundy B, Wenzel L, et al: Intraperitoneal cisplatin and paclitaxel in ovarian cancer. N Engl J Med 2006;354:34-43

16. Markman M, Bundy BN, Alberts DS, et al: Phase III trial of standard-dose intravenous cisplatin plus paclitaxel versus moderately high-dose carboplatin followed by intravenous paclitaxel and intraperitoneal cisplatin in small-volume stage III ovarian carcinoma: An intergroup study of the Gynecologic Oncology Group, Southwestern Oncology Group, and Eastern Cooperative Oncology Group. J Clin Oncol 2001 ;19:1001-1007

17. Hager ED, Dziambor H, Hohmann D, Muhe N, Strama H. Intraperitoneal hyperthermic perfusion chemotherapy of patients with chemotherapy-resistant peritoneal disseminated ovarian cancer. Int J Gynecol Cancer 2001;11(Suppl. 1):57–63.

18. Chatzigeorgiou K, Economou S, Chrysafis G, Dimasis A, Zafiriou G, Setzis K, et al. Treatment of recurrent epithelial ovarian cancer with secondary cytoreduction and continuous intraoperative intraperitoneal hyperthermic chemoperfusion (CIHIPECP). Zentralbl Gynakol 2003; 125:424–9.

19. Piso P, Dahlke MH, Loss M, Schlitt HJ. Cytoreductive surgery and hyperthermic intraperitoneal chemotherapy in peritoneal carcinomatosis from ovarian cancer. World J Surg Oncol 2004;2:21.

20. Ryu KS, Kim JH, Ko HS, Kim JW, Ahn WS, Park YG, et al. Effects of intraperitoneal hyperthermic chemotherapy in ovarian cancer. Gynecol Oncol 2004;94:325- 32.

21. Zanon C, Clara R, Chiappino I, Bortolini M, Cornaglia S, Simone P, et al. Cytoreductive surgery and intraperitoneal chemohyperthermia for recurrent peritoneal carcinomatosis from ovarian cancer. World J Surg 2004;28:1040–5.

22. Gori J, Castano R, Toziano M, Habich D, Staringer J, De Quiros DG, et al. Intraperitoneal hyperthermic chemotherapy in ovarian cancer. Int J Gynecol Cancer 2005;15:233–9.

23. 박상윤. 난소암의 복막내항암화학요법 (IPC; intraperitoneal chemotherapy) 과 수술중온열항암화학요법 (HIPEC; hyperthermic intraperitoneal chemotherapy). 대한 복막암 연구회 2006 년 9 월 2 일, 무주

57

24. 임명철, 배재만, 임소이, 박정열, 강석범, 서상수, 박상윤. Feasibility of intraoperative intraperitoneal hyperthermic chemotherapy for peudomyxoma peritonei and diffuse malignant peritoneal mesothelioma. 대한산부인과 학회 2006 년 9 월 29 일~30 일, 서울 그랜드 힐튼 호텔

25. Kusamura S, Younan R, Baratti D, Costanzo P, Favaro M, Gavazzi C, Deraco M. Cytoreductive surgery followed by intraperitoneal hyperthermic perfusion: analysis of morbidity and mortality in 209 peritoneal surface malignancies treated with closed abdomen technique. Cancer. 2006 Mar 1;106(5):1144-53.

26. Rossi CR, Foletto M, Mocellin S, Pilati P, De SM, Deraco M, Cavaliere F, Palatini P, Guasti F, Scalerta R, Lise M. Hyperthermic intraoperative intraperitoneal chemotherapy with cisplatin and doxorubicin in patients who undergo cytoreductive surgery for peritoneal carcinomatosis and sarcomatosis: phase I study. Cancer. 2002 Jan 15;94(2):492-9.

27. Walker JL, Armstrong DK, Huang HQ, Fowler J, Webster K, Burger RA, Clarke-Pearson D. Intraperitoneal catheter outcomes in a phase III trial of intravenous versus intraperitoneal chemotherapy in optimal stage III ovarian and primary peritoneal cancer: a Gynecologic Oncology Group Study. Gynecol Oncol. 2006 Jan;100(1):27-32.

28. Panteix G, Beaujard A, Garbit F, Chaduiron-Faye C, Guillaumont M, Gilly F, Baltassat P, Bressolle F. Population pharmacokinetics of cisplatin in patients with advanced ovarian cancer during intraperitoneal hyperthermia chemotherapy. Anticancer Res. 2002 Mar-Apr;22(2B):1329-36.

29. Schmid K, Boettcher MI, Pelz JO, Meyer T, Korinth G, Angerer J, Drexler H. Investigations on safety of hyperthermic intraoperative intraperitoneal chemotherapy (HIPEC) with Mitomycin C. Eur J Surg Oncol. 2006 Dec;32(10):1222-1225.

30. Gonzalez-Bayon L, Gonzalez-Moreno S, Ortega-Perez G. Safety considerations for operating room personnel during hyperthermic intraoperative intraperitoneal chemotherapy perfusion. Eur J Surg Oncol. 2006 Aug;32(6):619-24.

Protocol Version: 5.1
Version Date: 20200309
ClinicalTrials.gov Identifier: NCT00426257

The Randomized trial of Intraoperative Hyperthermic Intraperitoneal Chemotherapy Followed by Intravenous Chemotherapy in Patients with Advanced Epithelial Ovarian Cancer

The Randomized Trial of Intraoperative Hyperthermic Intraperitoneal Chemotherapy Followed by Intravenous Chemotherapy in Patients with Advanced Epithelial Ovarian Cancer

Principal Investigator:	Center for Gynecologic Cancer	Sang-Yoon Park, M.D.
Co-investigator:	Center for Gynecologic Cancer	Myong Cheol Lim, M.D.
	Department of Pathology	Chong Woo Yoo, M.D.
	Department of Radiology	Sun Ho Kim, M.D.
	Department of Nuclear Medicine	Tae-Sung Kim, M.D.
	Biostatistics Collaboration Team	Boram Park, Ph.D.
Research Coordinator:	Center for Gynecologic Cancer	Yohan Woo, R.N.
	Department of Cancer Control & Policy	Kyojin Bae, R.N.

Correspondence:

Sang-Yoon Park, M.D.
Center for Gynecologic Cancer
National Cancer Center
323 Ilsanro Ilsandonggu
Goyangsi Gyeonggido, Republic of Korea, 10408
TEL: +82-31-920-2381
FAX: +82-31-920-1238
Email: parksang@ncc.re.kr, sypark.ncc@hotmail.com

목차

1. 연구의 배경 45

2. 연구 목적 47

3. 시험변수 47

4. 임상시험 설계 48

5. 시험대상자 선택/참여중단 50

6. 임상시험 진행 53

7. 임상시험 변수의 수집 57

8. 통계분석계획 61

9. 자료관리 63

10. 윤리 및 규제관련 고려사항 63

11. 참고문헌 63

Abbreviation

**CA 125**	Cancer antigen 125
**CA 19-9**	Cancer antigen 19-9
**CBC**	Complete blood count
**CEA**	Carcinoembryonic antigen
**Cr**	Creatinine
**CT**	Computed tomography
**CTCAE**	Common Terminology Criteria for Adverse Events
**EORTC QOL-C30**	European Organization for Research and Treatment of Cancer Quality of Life Questionnaire – Core 30
**EORTC QOL-OV28**	European Organization for Research and Treatment of Cancer Quality of Life Questionnaire – Ovary 28
**GOG**	Gynecologic Oncology Group
**Hb**	Hemoglobin
**Hct**	Hematocrit
**HIPEC**	Hyperthermic intraperitoneal chemotherapy
**LFT**	Liver function test
**MDASI**	MD Anderson Symptom Inventory
**MRI**	Magnetic resonance imaging
**PET**	Positron emission tomography
**Plt**	Platelet
**RBC**	Red blood cell
**RCT**	Randomized Controlled Trial
**RECIST**	Response Evaluation Criteria Solid Tumors
**RFT**	Renal function test
**SGOT**	Serum Glutamic-oxaloacetic transaminase
**SGPT**	Serum Glutamic-pyrubic transaminase
**SOTOC**	Synoptic operative template for ovarian cancer
**WBC**	White blood cell

118 # Protocol Abstract

**Title**	The Randomized trial of Intraoperative Hyperthermic Intraperitoneal Chemotherapy Followed by Intravenous Chemotherapy in patients with Advanced Epithelial Ovarian Cancer
**Principal Investigator**	Sang-Yoon Park, M.D., Ph.D.
**Background**	It is important to know how to maximize the efficacy of anticancer therapy in the treatment of ovarian cancer, fallopian tube cancer, primary peritoneal cancer (hereinafter "ovarian cancer"). Maximal tumor resection followed by the timely administration of adjuvant chemotherapy is one of the important issues. Ovarian cancer has no effective early screening, and there are no specific symptoms that can be detected at an early stage, so it is found with peritoneal metastasis in two-thirds of the cases at the initial diagnosis. However, in most cases, the diagnosis is confined to the peritoneal cavity. In conclusion, the use of chemotherapy for ovarian cancer in the abdominal cavity could reduce the systemic side effects and enhance the therapeutic effect of chemotherapy by the administration of the site-specific chemotherapy. From three GOG randomized trials, published in 1996, 2001 and 2006, postoperative combined intraperitoneal and intravenous chemotherapy showed statistically significant progression-free survival and overall survival improvement compared to intravenous chemotherapy. The most recent study, the GOG # 172 study, reported a median survival of 65.6 months in the intraperitoneal chemotherapy group and a median survival of 49.7 months in the control group, the intravenous chemotherapy group, improving the survival rate of intraperitoneal chemotherapy. Therefore, the NCI clinical announcement in January 2006

suggested that intraperitoneal administration of chemotherapy after surgery should be considered as a standard treatment for ovarian cancer based on the better survival than intravenous administration.

However, there has been a growing debate in the intraabdominal chemotherapy group due to the increased incidence of various toxicities due to chemotherapeutic agents from the intraperitoneal administration, various complications caused by catheterization, and poor quality of life until 1 year after treatment. Although the early abortion rate of the use of intraperitoneal administration of chemotherapy was high in this case, the survival rate improved because it was interpreted that the initial intraperitoneal drug has been administered before the formation of the intraperitoneal adherence. Intraperitoneal chemotherapy during operation may prevent catheter-related complications and the deterioration of the quality of life due to the maintenance of chemotherapeutic agents in the abdominal cavity and may provide an equivalent or better therapeutic effect.

The intraperitoneal hyperthermic chemotherapy (HIPEC) has already been performed in patients with pseudomyxoma peritonei and peritoneal mesothelioma. Prospective randomized trials have reported good results in colon cancer with peritoneal dissemination. Pharmacokinetics studies of cisplatin as an intraperitoneal chemotherapy in advanced ovarian cancer patients was tolerable. Approximately 65% of the drugs administered intraperitoneally were absorbed into the body and about 45% of the drugs were absorbed into the tumor tissue. And only about 20% of the cells were absorbed systematically. Therefore, it is one of the ideal therapies that can maximize the effect through the accumulation of the chemotherapeutic agent in the target tissue and minimize the systemic side effect. Several investigators reported

that HIPEC could be used in many patients with ovarian, fallopian tubal and primary peritoneal cancer after cytoreductive surgery. But, the value of the studies has been limited because of the study design, retrospective nature. The safety of HIPEC as parts of surgical procedures has been confirmed in several studies, and in NCI of Milan, 209 patients with intraperitoneal cancer including ovarian cancer were prospectively evaluated for HIPEC. Although the prevalence of morbidity was 12% and the mortality rate was reported as 0.9%, the prospective study for ovarian cancer is needed. Complication after cytoreductive surgery followed by HIPEC was fistula or perforation (23 patients), intraabdominal bleeding (4 patients), and sepsis (4 patients). The complication directly related to HIPEC could not be separately identified from surgery-related complications. The rate of complication of HIPEC might be differentiated from that of cytoreductive surgery in the randomized trial.

In the National Cancer Center, HIPEC has been performed for peritoneal surface malignancies such as mesothelioma, pseudomyxoma peritonei, and recurrent ovarian cancer from 2001. Safe use of HIPEC after cytoreductive surgery for 16 cases of peritoneal surface malignancy has been reported to the 2006 Annual Meeting of Korean Society of Peritoneal Surface Malignancy and 2006 Annual Meeting of Korean Society of Obstetrics and Gynecology. US FDA-approved HIPEC device (Belmont Hyperthermia Pump HIPEC) that has been used in various countries and the HIPEC devices has been approved Korean FDA also. The device will be used in this clinical trial.

The current randomized trial could investigate the impact of HIPEC on survival outcomes and quality of life in patients with primary advanced ovarian cancer. Therefore, patients with primary

	advanced ovarian cancer and residual tumor <1cm after cytoreductive surgery will be allocated to the HIPEC group vs. no HIPEC to evaluate the progression-free survival rate, overall survival, safety, complications, and quality of life.
**Objective**	➢ Primary objectives: To assess progression-free survival outcome after intraoperative Hyperthermic Intraperitoneal Chemotherapy (HIPEC) vs. no HIPEC followed by intravenous chemotherapy in patients with primary advanced ovarian cancer.<br>➢ Secondary objectives:<br>◆ To assess recurrence-free, disease-free, or overall survival outcome after intraoperative HIPEC vs. no HIPEC followed by intravenous chemotherapy in patients with primary advanced ovarian cancer.<br>◆ To assess toxicity and quality of life after intraoperative HIPEC vs. no HIPEC followed by intravenous chemotherapy in patients with primary advanced ovarian cancer.<br>◆ To investigate the pattern of recurrence and treatment outcomes according to clinical variables including genomic status and immune-histochemical staining after intraoperative HIPEC vs. no HIPEC followed by intravenous chemotherapy in patients with primary advanced ovarian cancer.
**HIPEC and follow-up**	A. **Selection and registration of subjects**<br>Patients subject to clinical trials should be registered by the researcher before surgery.<br><br>B. **Preoperative evaluation**<br>Pre-treatment tests are performed to determine the subject's

selection and basic condition. Through pre-treatment screening, examine whether the subjects are unsuitable for the selection criteria and exclusion criteria, and exclude subjects with significant abnormalities.

1) Medical history and physical examination including gynecologic examination, height, weight, exercise ability, performance status, etc.

2) Basic clinical pathology examination

Blood test: WBC, RBC, Hemoglobin, Hematocrit, PLT, differential count, ABO/Rh

Biochemistry: Ca, P, glucose, uric acid, BUN, creatinine, cholesterol, protein, albumin, bilirubin, Alkaline phosphatase, AST, ALT, Electrolyte (Na, K, Cl), Mg, calcium-ionized

Blood coagulation test: PT/aPTT

Serology; VDRL, HBsAg, HIV Ab, HCV Ab

Urinalysis: gravity, pH, albumin, glucose, ketone, blood, urobilinogen, bilirubin, nitrite, microscopy

3) Preoperative evaluation of the quality of life: EORTC QOL-C30, EORTC QOL-OV28, MDASI

4) Tumor markers: CA-125

5) Chest PA

6) EKG

7) CT scan or MRI or PET-CT

C. Informed consent

Patients judged to be able to participate in clinical trials will be presented with a written explanation of the clinical trial and instructions for participation in clinical trials. Patients who agree

to participate in the clinical trial will receive preoperative documentation of the operation and anesthesia.

D) Intraoperative HIPEC

1) Location of the catheter & thermometer

- The inflow catheter will be placed in the pelvis, and the outflow catheter will be placed below the diaphragm.
- The HIPEC procedure after all surgical procedures including bowel anastomosis will be performed in closed method.

2) Perfusion

- Perfusion Belmont® Hyperthermia pump approved by KFDA will be used.
- The inflow fluid should be maintained at approximately 1 L per minute.
- Shaking the patients from side to side for 90 minutes to help distribute chemotherapeutic agents evenly throughout the whole peritoneal cavity.

3) Regulation of thermometer

- Measure the core temperature using a Nasopharyngeal-temperature probe.
- 0.9% saline solution will be infused from the HIPEC machine into the abdominal cavity with a flow rate of 800mL/min, with the target temperature of 41.5 ℃ (range, 41-42 ℃). The temperature of inflow fluid and flow rate will be adjusted based on the size of the abdominal cavity and general condition including body temperature.
- Measure the body temperature every 5 minutes during perfusion so that the core temperature does not exceed 39 ℃.

4) Safety in the thermometer of the Hyperthermia pump

- **Temperature monitoring**
  - Temperature range: Designed to heat within 37 ℃ to 46 ℃ safe range in HIPEC
  - Continuously temperature monitoring system on display: (mechanical surveillance)
  - True pump output temperature 4 user-controlled patient temperature (T1, T2, T3, T4)

- **Safety function about over temperature**
  - When an unsafe condition occurs
    A. Alarm
    B. Alarm message display
    C. Stop the pump, stop warming, and clamp the patients' line valve

- **Double check of the temperature monitoring by mechanically or manually.**

- During the procedure, the medical staff will measure and record the temperature of inflow and outflow tubes from the monitor of the HIPEC machine every 5 minutes. Simultaneously, the temperature of the inflow tube will be manually checked and recorded every 5 minutes.

- If the core temperature exceeds 39 ^{o}C, immediately contact other health care providers and anesthesiologists and take the following measures:
  ① Stop heating
  ② Management from anesthesiologic department

i. Ice pack apply around head
ii. Rapid inflow of cold saline
iii. Cooling of blanket

③ Management in the operative field

i. Consider open the surgical wound
ii. Drain out of the fluid in the peritoneal cavity
iii. Direct irrigation with cold saline

5) Administration of cisplatin

- If the intraperitoneal temperature is maintained above 41.5 ^{o}C, cisplatin (75 mg/m^2) will be perfused for 90 minutes. Before and after perfusion, intravenous fluid, anti-vomiting agent, and amifostine will be administered adequately.

6) Postoperative recovery

• The patients will be kept in the intensive care unit to postoperative 1 day. Enteric diet, intravenous antibiotics, and anti-thrombotic prophylaxis will follow the Critical Pathway of Cytoreductive Surgery for Peritoneal Surface Malignancies.

F. Postoperative chemotherapy

If the patients are tolerable to general diet without evidence of active infection and with an acceptable condition for chemotherapy, intravenous chemotherapy with paclitaxel and carboplatin will be given to the patient. If the patient has progressive disease or intolerable toxicity, alternative cytotoxic treatments including topotecan, pegylated liposomal doxorubicin, docetaxel, or vinorelbine or targeted therapy including the olaparib or bevacizumab could be used optimally.

	**G. Post-treatment surveillance**<br>1) During adjuvant chemotherapy, disease status including CA-125 and CT, toxicity using CTCAE 4.03, quality of life (EORTC QOL-C30, EORTC QOL-OV28, MDASI) will be evaluated. After completion of chemotherapy, surveillance will be done every 3 months during the first 2 years after treatment, every 6 months for 3 years. After 5 years from completion of treatment, the patient will be under annual surveillance. MRI or PET/CT will be selectively used to evaluate the disease status.<br>2) The quality of life will be evaluated preoperatively, 7 days after the operation, at 3 and 6 cycles of chemotherapy, and every 3 months during the first year after treatment, every 12 months for 4 years.<br><br>H. Intraperitoneal tissue and blood sample will be harvested in patients with written informed consent before randomization for the translational researches.
**Study period**	Date of IRB approval – Dec 31, 2023
**Criteria**	**Eligibility:**<br>**Inclusion Criteria:**<br>1. Disease status primary ovarian cancer, tubal cancer, and primary peritoneal cancer (Stage III or IV)<br>A. Primary debulking surgery group who are expected to be optimally debulked.<br>B. Three cycle of neoadjuvant chemotherapy with carboplatin and paclitaxel in case of difficult optimal debulking with primary surgery or in case with poor general condition.<br>2. Residual tumor < 1cm after completion of cytoreductive

surgery

3. Age < 75 year
4. Expected survival > 3 months
5. Performance status: ECOG 0-1
6. Adequate bone marrow function Hb ≥8 g/dl (After correction in case of iron deficient anemia) WBC ≥ 3,000/mm3, Platelet ≥ 100,000/mm3
7. Adequate renal function Creatinine ≤ 1.5 mg/dl
8. Adequate hepatic function Bilirubin ≤ 1.5 mg/dl and AST and ALT ≤ 80 IU/L
9. Optimal cardiopulmonary function for surgery
10. Voluntary participation after getting written informed consent.

Exclusion Criteria:

1. Unresectable extraperitoneal metastasis (brain, bone, lung parenchyma, or lymph node)
2. Suboptimal debulking (residual tumor ≥ 1cm)
3. Previous history of other malignancies (except excision of skin cancer)
4. Serious heart disease or renal failure
5. Serious cardiopulmonary insufficiency
6. Uncontrolled infection
7. Uncontrolled intercurrent disease
8. Psychogenic disorder
9. Patients who are suitable candidates by legally
10. Pregnant or breast-feeding patients

	11. Patients who are unsuitable candidates by doctor's decision<br>12. Cancer tissue is not confirmed during surgery after neo-adjuvant chemotherapy
**Intervention**	If the intraperitoneal temperature is maintained above 41.5 ^{o}C, cisplatin (75 mg/m^2) will be perfused for 90 minutes. Before and after perfusion, intravenous fluid and anti-vomiting agent will be administered adequately. Before perfusion, amifostine (Sodium thiosulfate) 4g/m^2 will be infused. After perfusion, amifostine (Sodium thiosulfate) 12g/m^2 will be administered for 6 hours.
**Statistical Analysis**	**Sample size calculation**<br><br>Total number of subjects: 184<br><br>We assumed that the median survival times are 1.5 year for control group and 2.0 year for treatment group, that is a hazard ratio (HR) of 0.75. The total duration of the study is 8 years, which is the accrual time of 6 years plus the follow-up period of 2 years. A log-rank test with a total sample size of 184 subjects (per each group 92 subjects) achieves 82.3% power at a one-sided 0.20 significance level to detect a HR of 0.75. We additionally calculated that a log-rank test with the sample size of 184 subjects achieves 68.8% power at one-sided 0.1 significance level to detect a HR of 0.75. This result was calculated using the PASS version 2019.

1. 연구의 배경

1.1. 난소암에서 시행한 수술 중 복강내온열항암화학요법에 대한 국내 외 연구 및 기술 현황.

1.1.1. 외국 현황

Panteix G 등은 난소암 환자 16 명에 대한 phase I 연구 (cisplatin 60mg, 80mg, 100mg)를 시행하였다. HIPEC 시행 후 평균 65%가 체내로 흡수되는데, 최대 혈장 농도를 보이는 시간은 투여 후 1~1.5 시간 뒤였고, 제거 반감기는 80 시간이었다. 투여된 cisplatin 의 20%만 소변으로 배출되고, 복강내 투여된 cisplatin 의 45%는 종양조직에 흡수되므로 전신 부작용은 줄이고, 치료 효과를 최대화할 수 있는 좋은 치료(진행된 난소암에서 3 년 생존율: 37.5%, 7 년 생존율: 12.5%)라 할 수 있다. 난소암, 난관암 및 원발성 복막암에 대한 종양감축술 후 수술 중 복강내 온열항암화학요법은 세계적으로 여러 보고자들 (독일의 Hager ED, Chatzigeorgiou K, 이탈리아의 Piso P, Zanon C, Raspagliesi F 아르헨티나의 Gori J)이 연구성과를 보고하였다. 수술 중 복강내온열항암화학요법 시행시 이중 latex glove 착용으로 의료인에게 chemotherapeutic agent 가 피부로 흡수되는 것이 방지되는 것을 확인하였고 (Schmid K, Eur J Surg Oncol, 2006), 의료인 및 환자에게 수술 중 복강내온열항암화학요법이 안전하게 시행될 수 있음이 보고되었다 (Gonzalez-Bayon L, Eur J Surg Oncol, 2006). 또한, 밀란의 NCI 에서 난소암을 포함한 복강 내 암환자 209 명에 대한 수술 중 복강내온열항암화학요법에 대한 전향적 2 상 임상연구를 시행하여 cancer 지에 보고하였는데, 주요 유병율이 12%였고, 사망률이 0.9%였다. 주요 합병증은 문합부위 누공이나 천공(23 명), 복강내 출혈(4 명), 패혈증(4 명)이 있었다. 난소암에서 시행된 대부분의 연구에서 알 수 있듯이 수술 전 여러 번의 항암화학요법 후에도 재발한 난소암 환자에게 안전하게 시행되었으므로, 항암화학제에 노출이 되지 않은 환자에게 시행된다면 보다 안전하고 보다 좋은 성적을 보일 것으로 기대된다.

1.1.2. 국내 현황

난소암, 난관암 및 원발성 복막암의 수술적 치료 및 수술 중 복강내온열항암화학요법에 대하여 본 연구자 팀(국립암센터 자궁암센터) 외에는 카톨릭대학교 이준모 교수팀이 연구와 치료를 시도하고 있으며, 일부 성과를 보고하였지만, 후향적 연구로 다양한 환자군을 포함하고 있어서, safety 와 feasibility 확인 외에 표준 진료 지침으로 제시되기에는 한계가 있는 것으로 지적되고 있다.

본 연구자 팀(국립암센터 자궁암센터)은 2001 년부터 현재까지 복막 위점액종(난소기원-4 례, 일차성 복막암 기원-1 례, 충수돌기기원 3 례) 및 복막 중피종(2 례), 재발성 난소암 (6 례) 총

16 례에 대하여 종양감축술 및 수술 중 복강내온열항암화학요법을 시행하였다. 수술로 인한 사망률이나, 누공, 농양형성, 백혈구 감소 등과 같은 주요 합병증은 없었고, 안전하게 시술될 수 있음을 2006 년 복막암 연구회, 산부인과 학회에 보고하였다. 2007 년 1 월부터 2008 년 2 월까지 31 명을 대상으로 phase II 연구를 수행하여 세계 최초로 2009 Annals of Surgical Oncology 에 HIPEC 의 유용성과 안전성을 보고하였다.

1.2. 난소암 임상 양상과 연구의 필요성

난소암 치료는 항암제 치료의 효과를 어떻게 극대화하느냐가 관건이라 사료되며 이의 핵심은 최대한의 종양감출술을 시행하여 최대잔여종양의 직경을 최소화하는 것과 수술 후 항암화학제 투여 경로와 투여시기의 설정에 달려 있다고 할 수 있다. 난소암은 복강 내에 국한되어 발생하고, 재발하더라도 대개 복강 내에 국한되어 있다. 그래서 복강내 항암화학요법을 시행하면, 전신투여 보다 복강내 치료효과는 높이고 전신적 부작용은 감소시킬 수 있을 것이라 생각되고 있으며, 이미 여러 연구들을 통하여 밝혀졌다. 2006 년 1 월 NCI clinical announcement 를 통해 난소암 치료의 수술 후 표준 항암화학요법은 복강내 항암화학요법으로 바뀌어 가고 있는 추세이다.

앞으로 난소암에서 수술 후 항암화학요법의 효과를 최대화 하기위해서 항암화학요법의 투여시기, 투여효과를 최대화하여야 생존율의 향상을 기대할 수 있을 것이다. 이러한 방법은 현재 복막 위점액종, 복막 중피종에 적용되고 있는 수술 중 복강내 온열 항암 화학 요법이라 할 수 있다. 수술 중 항암화학요법을 복강내 시행함으로 수술 후 발생하는 유착에 의한 항암화학제의 고른 분포의 저해가 없으며, 종양감축술 시 탈락된 암세포를 세척하고, 항암화학제로 살상할 수 있어서 상처부위로 부착 및 성장을 원천적으로 차단할 수 있는 큰 장점이 있다. 또한 온열은 온열 자체의 세포독성 이외에 항암제의 세포독성을 향상시킨다. 이는 약물의 세포막 투과성 증가, 세포막 회복의 감소, 온열에 따른 약의 대사항진 등이 작용하기 때문으로 설명되고 있다. '수술 중 복강 내 온열 항암제치료(HIPEC)'은 1980 년대 이후 복막가점액종, 위암, 대장암, 재발성 난소암 등에 보고되고 있었으며, 이탈리아, 독일 등의 국가에서 이미 난소암, 일차성 복막암에 대한 일차적 치료로 1990 년대 후반부터 점차 시도되고 있으며, 그 안정성 및 효용성이 보고되고 있는 실정이다. 이에 국립암센터 자궁암센터는 본 연구에서 난소암, 난관암, 일차성 복막암에 대하여 적절한 종양감축술이 시행된 경우 HIPEC 을 시행하고, 수술 후 식이가 가능하고, 활동성 감염 등의 징후 없는 상태로 항암화학요법이 가능하다고 판단되면 paclitaxel 과 carboplatin 으로 정맥 항암화학요법을 시행하여 총 6 차례의 항암화학요법을 시행하고자 한다.

현재, 이러한 치료효과를 확인하기 위한 연구들이 계획되고 있다. 아직 현재까지 난소암에서의 종양감축술 후 수술 중 복강내항암화학요법이 생존율과 삶의 질 향상에 도움이 되는지에 대한 전향적인 연구는 아직 보고가 없는 실정으로, 이에 대한 연구 및 검토가 시급한 실정이다.

이미 복강내 항암화학요법이 정맥항암화학요법보다 생존율을 증가시킴이 미국 부인종양학회의 3 개의 대규모 Randomized control study 로 입증되었고, 2006 년 복강내 항암화학요법이 선호되는 치료법으로 사용되어야 한다고 NCI clinical announcement 가 있었지만, 독성과 삶의 질 감소로 임상적용에 한계가 있었던 것이 분명하다. 이론적으로 복강내 항암화학요법의 장점 (유착이 없는 수술직후 복강내 직접 투여, 온열로 항암화학요법의 효과 증대 등)을 극대화하고 단점(도관으로 인한 복통 및 감염 등)을 최소화할 수 있을 것으로 제시되고 있다. 또한 2009 년 국립암센터에서 세계 최초로 Annals of surgical oncology 지에 일차성 난소암에 대한 phase II 연구결과 안전하고 유용하다는 점을 보고하였다. 이미 수술 후 복강 내 항암화학요법이 생존율은 유의하게 증가시키지만, 삶의 질 감소로 임상에 널리 사용되지 못하는 점을 고려할 때, HIPEC 에서 안전성 및 효과 이외에 삶의 질에 미치는 영향이 매우 중요한 평가 항목이라고 할 수 있다. 따라서, 일차성 난소암에서는 phase II RCT 를 통해서 비교연구가 필요하며, 특히 삶의 질에서의 결과를 확인하는 것이 매우 중요할 것으로 지적되고 있다.

따라서 본 센터에서는 난소암 환자 중 적절한 최대종양감축술이 가능할 것으로 판단되는 환자에 대하여 종양감축술을 시행하고, 수술 중 복강내항암화학요법이 시행하여, 수술 중 복강내항암화학요법과 관련된 합병증을 분석 비교하며, 이러한 환자의 삶의 질을 전향적 연구를 통하여 평가하고자 한다.

2. 연구 목적

2.1. Primary Objectives:

일차성 난소암 환자에서 수술 중 복강내온열항암화학요법 시행 여부에 따른 무진행생존율(PFS)을 관찰한다.

2.2. Secondary Objectives:

2.2.1. 일차성 난소암 환자에서 수술 중 복강내온열항암화학요법 시행 여부에 따른 무재발생존율(RFS), 무병생존율(DFS), 생존율(OS)를 관찰한다.

2.2.2. 일차성 난소암 환자에서 수술 중 복강내온열항암화학요법 시행 여부에 따른 독성과 삶의 질을 분석한다.

2.2.3. *BRCA1*, *BRCA2* 등 유전자 상태와 면역조직화학염색 등 임상변수에 따른 재발의 유형과 치료 성적을 탐색적으로 비교한다.

3. 시험변수

3.1. 인구통계 및 베이스라인 특성

베이스라인 특성은 표준 인구통계(예: 연령, 체중, 신장, Performance Status 등)와 질병 특성, 종양조직학(장액성선암/투명세포암 등), 의학적 병력(수술력 포함) 및 종양 치료력을 포함한다. 종양학 병력은 백금제제, 파클리탁셀, 베바시주맙 등의 항암화학요법을 포함한다

3.2. 일차 및 이차 평가변수

3.2.1. 일차평가변수

무작위 배정일로부터 치료 종료 후 첫 영상학적 재발일 까지의 시간(Month)을 PFS 로 정의한다. 재발 전 사망한 경우, 무작위 배정일로부터 모든 원인으로 인한 사망일까지의 시간을 PFS 에 포함한다.

3.2.2. 이차평가변수

3.2.2.1. Recurrence free survival (RFS)는 무작위 배정일로부터 난소, 난관, 복막암의 재발까지의 시간(Month)으로 정의한다.

3.2.2.2. Disease free survival (DFS)는 난소, 난관, 복막암의 무병 상태부터 질환의 재발까지의 시간(Month)으로 정의한다.

3.2.2.3. Overall Survival (OS)는 무작위 배정일로부터 사망일까지의 시간(Month)으로 정의한다.

3.2.2.4. 독성은 수술 (±HIPEC) 후 회복기간, 퇴원시점, 항암시작, 마지막 항암 후 6 주까지 시기를 나누어 분석한다.

3.2.2.5. 삶의 질 분석은 등재 시점부터 5 년까지 조사한다.

3.2.2.6. Genomic test 및 immunohistochemical staining 은 난소암의 진단, 치료 특히 온열 치료에 관련 marker 에 대해서 탐색적 연구를 수행한다.

3.2.2.7. 각종 혈액검사 결과, 특히 leukocyte subset 의 변화에 따른 생존, 재발에 대한 영향을 탐색적으로 분석한다.

3.3. 기타 이차 평가변수

3.3.1. HIPEC 치료와 관련된 안전성 확인.

3.3.1.1. 이상반응은 CTCAE 4.03 에 따라 용어를 정하여 수집한다.

- Post OP Adverse Event: 무작위배정 시점으로부터 다음 항암 시작 전까지
- Chemotherapy Adverse Event: 첫 항암화학요법 시작일로부터 마지막 항암화학요법 시행일 이후 6 주째 시점까지

3.4. 탐색적 평가변수

3.4.1. 수술날짜 및 수술명, 잔류종양 및 기타 수술 관련 정보를 수집한다.

3.4.2. 신보조요법(Neo-Adjuvant Chemotherapy; NAC)및 보조항암화학요법(Adjuvant Chemotherapy: AC) 정보를 수집한다.

3.4.3. 입원과 관련한 정보를 수집한다(재원일, ICU 재원일 수 등).

3.4.4. 기 시행된 BRCA 정보 및 genomic data 를 분석 및 수집한다.

3.4.5. SOTOC 을 사용하여 Tumor Burden 을 평가한다.

4. 임상시험 설계

본 연구는, 과거 Single Arm 으로 진행하다, 연구 방법을 변경하여 Two Arm 으로 변경하여 진행하였습니다. Single Arm 은 Pilot Study 로 통계분석에서 제외될 것입니다.

4.1. Single Arm

4.1.1. Single Arm Schema

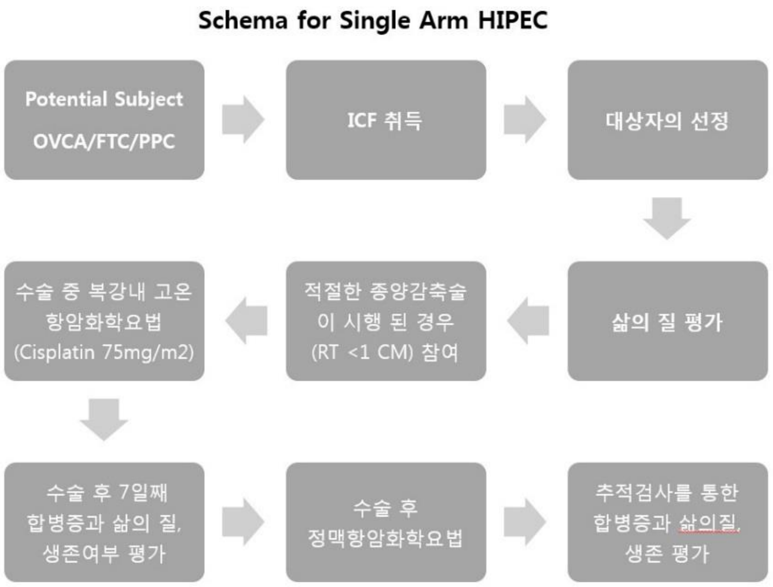

4.2. Two Arm

Two Arm 은 RCT(Randomized Controlled Trial)로 다음과 같이 시행됩니다.

4.2.1. Two Arm Schema

Schema for Two Arm HIPEC

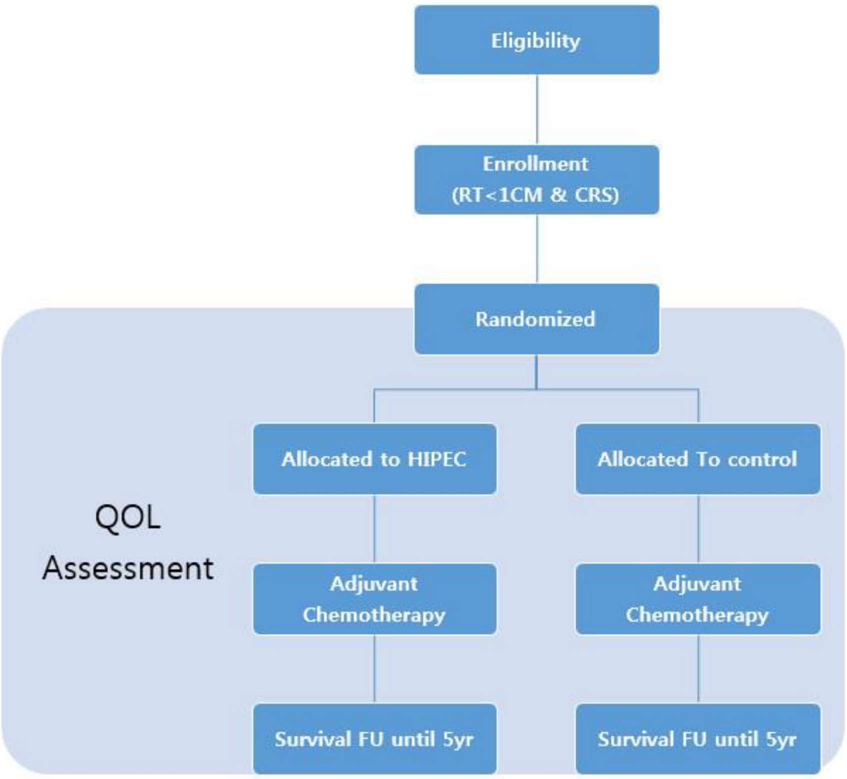

5. 시험대상자 선택/참여중단

5.1. 계획된 시험 대상자 수

5.1.1. Single Arm

본 기관에서 모두 등록하며, 총 대상자는 48 명이다.

5.1.2. Two Arm

다기관 연구로 본 기관과 아주대학병원에서 대상자를 등록하며, 총 대상자는 184 명이다.

Ajou University Hospital	Suk Joon Chang, M.D.
Chungnam University Hospital	Heon Jong Yoo, M.D.
Herings	Byung Ho Nam, Ph.D.

5.2. 시험모집단

5.2.1. Single Arm 선정기준

본 임상시험 선정에 적합 하려면, 시험대상자가 다음 기준에 부합해야 한다.

5.2.1.1. 질환의 상태에 따른 조건, 3 기이상의 난소암(난관암, 일차성 복막암 포함)

5.2.1.2. 종양감축술 후 잔류종양의 최대 직경이 1Cm 미만인 경우

5.2.1.3. 연령이 75 세 이하인 환자

5.2.1.4. 임상적 판단으로 환자의 예상수명이 최소한 3 개월 이상인 경우

5.2.1.5. Performance Status 가 ECOG 0-1 인 환자

5.2.1.6. 적절한 골수 기능을 가진 경우

혈색소 ≥10 g/dl(단순한 철결핍성 빈혈의 경우는 교정 후)

백혈구 ≥ 3,000/mm3,

혈소판 ≥ 100,000/mm3

5.2.1.7. 적절한 신기능을 가진 경우

크레아티닌 ≤ 1.5 mg/dl

5.2.1.8. 적절한 간기능을 가진 경우

빌리루빈 ≤ 1.5 mg/dl 이고, 간효소치(AST/ALT) ≤ 80 IU/L

5.2.1.9. 심장, 폐 등에 현저한 기능장애가 없는 경우

수술 전 폐기능 검사 상 이상을 보이거나 심전도와 흉부 방사선 촬영상 이상을 보인 경우, 그리고 이전에 심장 및 폐질환이 있었던 경우에는 순환기 및 호흡기내과와의 협진으로 기능장애로 인한 수술 관련 위험성이 크지 않다고 판단되는 경우

5.2.1.10. 수술 전에 본 임상시험에 자의로 참여를 결정하고 환자동의서에 서명한 경우

5.2.2. Single Arm 제외기준

다음 조건 중 1 가지 이상에 해당되는 환자는 본 임상시험에 참여할 수 없다.

5.2.2.1. 복강을 벗어난, 즉 뇌, 골, 폐, 경추늑골근 림프절, 쇄골하 림프절 등에 전이가 있는 경우

5.2.2.2. 수술 중 적절한 종양감축술이 불가능했던 환자(잔류종양 1Cm 이상)

5.2.2.3. 여성생식기암 이외의 다른 암을 앓은 적이 있는 환자(단, 피부암으로 근치적 절제를 받은 환자)

5.2.2.4. 심각한 심질환 및 신부전이 있는 경우

5.2.2.5. 심각한 폐질환 및 폐 기능부전이 있는 경우

5.2.2.6. 비경구 항생제 투여를 요하는 활동성 세균 감염증이 있는 환자

5.2.2.7. 기타 심각한 내과적 질환이 동반된 환자

5.2.2.8. 정신질환이 있는 경우

5.2.2.9. 법적으로 임상시험에 참가가 불가능한 환자

5.2.2.10. 임신중이거나 수유중인 환자

5.2.2.11. 기타 의사의 판단으로 임상시험에 참가가 불가능한 환자

5.2.3. Two Arm 선정기준

본 임상시험 선정에 적합하려면, 시험대상자가 다음 기준에 부합해야 한다.

5.2.3.1. 질환의 상태에 따른 조건

5.2.3.2. 3 기 이상의 일차성 난소암(난관암, 일차성 복막암 포함)으로 종양감축술 후 잔류종양의 최대 직경이 1Cm 미만인 경우

5.2.3.3. 연령이 75 세 이하인 환자

5.2.3.4. 임상적 판단으로 환자의 예상수명이 최소한 3 개월 이상인 경우

5.2.3.5. Performance Status 가 ECOG 0-1 인 환자

5.2.3.6. 적절한 골수 기능을 가진 경우

혈색소 $\geq$8 g/dl(단순한 철 결핍성 빈혈의 경우는 교정 후)

백혈구 $\geq$ 3,000/mm3,

혈소판 $\geq$ 100,000/mm3

5.2.3.7. 적절한 간기능을 가진 경우

크레아티닌 $\leq$ 1.5 mg/dl

5.2.3.8. 적절한 간기능을 가진 경우

빌리루빈 $\leq$ 1.5 mg/dl 이고, 간효소치(AST/ALT) $\leq$ 80 IU/L

5.2.3.9. 심장, 폐 등에 현저한 기능장애가 없는 경우

수술 전 폐기능 검사 상 이상을 보이거나 심전도와 흉부 방사선 촬영상 이상을 보인 경우, 그리고 이전에 심장 및 폐질환이 있었던 경우에는 순환기 및 호흡기내과와의 협진으로 기능장애로 인한 수술 관련 위험성이 크지 않다고 판단되는 경우

5.2.3.10. 수술 전에 본 임상시험에 자의로 참여를 결정하고 환자동의서에 서명한 경우

5.2.4. Two Arm 제외기준

다음 조건 중 1 가지 이상에 해당되는 환자는 본 임상시험에 참여할 수 없다.

5.2.4.1. 복강을 벗어난, 즉 뇌, 골, 폐, 경추늑골근 림프절, 쇄골하 림프절 등에 전이가 있는 경우(단, 국소 전이로 완전 절제가 가능한 경우 및 수술 전 항암화학요법을 통해 영상검사상 병변이 사라진 경우는 제외)

5.2.4.2. 수술 중 적절한 종양감축술이 불가능했던 환자(잔류종양 1Cm 이상)

5.2.4.3. 여성생식기암 이외의 다른 암을 앓은 적이 있는 환자(단, 피부암으로 근치적 절제를 받은 환자)

5.2.4.4. 심각한 심질환 및 신부전이 있는 경우

5.2.4.5. 심각한 폐질환 및 폐 기능부전이 있는 경우

5.2.4.6. 비경구 항생제 투여를 요하는 활동성 세균 감염증이 있는 환자

5.2.4.7. 기타 심각한 내과적 질환이 동반된 환자

5.2.4.8. 정신질환이 있는 경우

5.2.4.9. 법적으로 임상시험에 참가가 불가능한 환자

5.2.4.10. 임신중이거나 수유중인 환자

5.2.4.11. 기타 의사의 판단으로 임상시험에 참가가 불가능한 환자

5.2.4.12. NAC(Neoadjuvant Chemotherapy) 후 수술을 진행했을 때, 수술 중 Tissue 의 확인이 되지 않는 환자.

5.3. 조기 임상시험 참여중단

대상자는 언제라도 어떤 사유로든 이후 치료에 영향을 받지 않고 임상시험 참여를 중단할 권리가 있다. 제외기준은 다음과 같다.

5.3.1. 환자가 원하는 경우(동의철회)

5.3.2. 다른 심각한 내과적 이상소견 등 중대한 이상 반응 발생 시

5.3.3. 선정제외기준을 위반한 경우

5.3.4. 시술에 따른 직접적인 부작용이 예상되는 경우

6. 임상시험 진행

6.1. 환자 등록과 무작위 배정, 임상시험용 의약품의 개시

6.1.1.1. 임상시험과 관련된 어떠한 절차도 수행하기 전에 잠재적인 환자로부터 서명된 시험대상자 동의서를 확보한다.

6.1.1.2. 잠재적인 환자에게 Screening No 를 부여한다. C222-001 …..

6.1.1.3. 환자의 등록 적합성을 결정한다. 수술 중 적절한 종양감축술이 이루어 졌다고 연구자가 판단하는 경우 Random 배정을 진행한다.

6.1.1.4. Random 배정 시, 독립적 무작위 넘버 배정자를 통해, Random Number 를 받는다. 무작위넘버 배정자는 Random Table 을 이용하여 HIPEC Allocation 을 진행한다. HIPEC Allocation 군의 경우 NH01, NH02, NH03…..과 같이 번호가 배정되며, HIPEC Control 군의 경우 NN01, NN02, NN03….과 같이 번호가 배정된다. 1:1 의 확률로 무작위배정 될 것이다.

6.2. 수술 전 Baseline 평가

Baseline 평가를 시행하고, Two Arm 연구에서는 수술 전날 오후부터 수술실에 도착하기 전까지 Normal Saline 1.5L 를 12 시간 동안 투여한다. 이때, 대상자의 병력과 특성에 따라 수액의 종류 및 주입용량을 연구자 판단에 따라 조절할 수 있다.

6.3. 수술 중 HIPEC 시행 방법

6.3.1. 수술 중 적절한 종양감축술이 이루어졌다고 연구자가 판단하는 경우 Random Table 을 통해 Random 배정을 진행하여 HIPEC 군으로 배정되면 아래의 절차를 수행한다.

6.3.2. Catheter 와 Thermometer 의 위치를 확인한다

6.3.2.1. Inflow catheter 는 골반강 내에, outflow catheter 는 횡격막과 간 사이에 위치시킨다.

6.3.2.2. 복강 내 온도측정을 위한 thermometer 을 횡격막 밑에, 골반강 내 위치시킨다. 복강 내 관류를 위해서 피부연속봉합으로 폐쇄된 복강 공간을 만든다. 절개부위중심 2-5Cm 정도는 봉합하지 않은 채 노출시켜서 처음 관류액이 복강을 채울 때 공기가 빠질 수 있도록 하며, 관류액이 복강을 채우면, 완전 봉합한다.

6.3.2.3. 관류 시 Belmont® Hyperthermia pump approved by KFDA 를 사용한다.

6.3.2.4. 관류하는 90 분동안 환자의 측면을 흔들어서 항암화학제가 복강내에 고루 분포할 수 있도록 한다.

6.4. 온도 및 속도 조절

6.4.1.1. Nasopharyngeal-temperature probe 를 이용하여 core temperature 를 측정한다.

6.4.1.2. 관류를 시작 시 42.5 ˚C, 0.9% saline solution, 800mL/min, inflow 로 관류를 시작하여 복강 내 온도를 41.5 (41~42) ℃로 유지하도록 한다. 복강 내 목표 온도에 도달하면 42 ℃로 inflow 를 유지하고, 목표 온도의 변화만큼 inflow 온도를 변경하여 목표온도를 유지하도록 한다. Abdominal cavity 환경 및 체온 등 환자의 전신 상태에 따라서 inflow fluid 의 온도와 flow rate 를 조절한다.

6.4.1.3. 관류하는 동안 5 분마다 체온을 측정하여 core temperature 가 39 ℃를 넘지 않도록 한다.

6.5. Hyperthermia Pump 온도 안전성 모니터링

6.5.1. 기계적감시

6.5.1.1. Temperature range: 37 ˚C-46 ˚C 로 HIPEC 시 안전한 범주 내에서만 가온 되도록 설계됨

6.5.1.2. Continuously temperature monitoring system on display: (기계적 감시):
True pump output temperature 4 user-controlled patient temperature (T1, T2, T3, T4)

6.5.1.3. Safety function (over temperature 관련):

- **Unsafe condition 이 발생할 경우 (heating fault or over temperature)**
  - Alarm 경고음
  - 알람 메시지 표시
  - 펌프정지, 가온 정지, 환자 라인 밸브 클램프 (recirculation)

6.5.2. 인적 감시 병행

6.5.2.1. 시술이 진행되는 동안은 담당 의료진이 5 분마다 온도를 측정하여 기록한다. 온도 측정과 별개로 체내로 투입되는 관류관과 체외로 배출되는 관류관을 손으로 온도를 직접 확인하고 이를 기록지에 기재한다

6.5.2.2. Core temperature 가 39 ˚C 초과 시 즉시 다른 의료진들과 마취과에 연락을 취하며 다음의 조치를 취한다.

- **가온 정지**
- **마취과 조치**
  - **Ice pack apply around head**
  - **Rapid inflow of cold saline**
  - **Cooling of blanket**
- **수술 필드 내 조치**

■ **개복고려 & 복강 내 직접 관류 고려**
● **Drain out 후 복강 내에 가온 되지 않은 Saline 투여**

6.6. Cisplatin 투여

6.6.1 수술 중 종양을 최대한 제거한 후 41.5 ℃ (범위, 41-42 ℃)의 수액과 항암제(cisplatin 75 mg/m^2)를 90 분간 복강 내에 관류시킨다. 관류 전, 관류 후에 적절한 수액을 공급하고, 필요시 항구토제를 투여한다.

6.6.2. Cisplatin 독성 예방을 위해 cisplatin 투여 직전 Amifostine(Sodium thiosulfate) 4g/m^2 를 투여하고 cisplatin 투여 후 Amifostine(Sodium thiosulfate) 12g/m^2 를 6 시간 동안 점적 정주한다.

6.6.3. Hyperthermia Pump 보관 및 관리방법 Hyperthermia pump 는 연구에 사용되는 의료기기로 의료기기 보관소에 보관해야 하나 수술 시행 중 대상자에게 적용하는 특수한 상황을 고려하여 수술실 내에 보관하고 책임연구자 감독하에 관리 업무를 위임받은 담당자가 관리한다

6.7. 수술 후 회복과정

6.7.1 수술 후 통상 중환자실에서 수술 후 1 일까지 관찰한다.

6.7.2. 식이, 항생제 사용, 혈전방지 등 수술 후 회복과정은 원내 Critical Pathway 를 따른다.

6.8. 수술 후 항암화학요법

수술 후 식이가 가능하고, 활동성 감염 등의 징후 없는 상태로 항암화학요법이 가능하다고 판단되면 질환의 상태에 따라서 paclitaxel & carboplatin 정맥 항암화학요법 3 주 간격으로 총 6 회 투여한다. 항암화학요법을 시작하거나, 독성 등으로 약제를 중단 혹은 감량하거나, 재발하는 등의 항암화학요법을 변경해야 하는 경우에는 기관의 지침에 의한다. 재발 시 paclitaxel & carboplatin, topotecan, docetaxel, pegylated liposomal doxorubicin, vinorelbine, olaparib, niraparib, bevacizumab 등약제를 사용할 수 있다.

6.9. 항암화학요법 후 추적 관찰

6.9.1. Single Arm 추적관찰

6.9.1.1. **재발 시점 확인 혈액검사:** 항암화학요법 2 주기, 4 주기, 6 주기에 신체검사, 종양표식자(CA-125, CA19-9, CEA) 검사를 시행한다. 항암화학요법 시행 후 1 년까지는 3 개월 간격으로, 이후 2 년간 6 개월 간격으로, 이후 2 년간 1 년 간격으로 신체검사, 종양표식자(CA125) 검사를 시행한다.

6.9.1.2. **재발 시점 확인 영상검사**: 항암화학요법 3 주기, 6 주기에 영상적 검사를 시행한다. 항암화학요법 시행 후 1 년까지는 3 개월 간격으로, 이후 2 년간 6 개월 간격으로, 이후 2 년간 1 년 간격으로 영상적 검사를 시행한다.

6.9.1.3. **삶의 질 평가:** 수술 전, 수술 후 일주일 뒤, 항암화학요법 3 주기, 6 주기 마다, 항암화학요법 후 1 년까지는 3 개월간격으로, 이후 매년마다 환자의 삶의 질을 평가한다.

6.9.2. Two Arm 추적관찰

6.9.2.1. 환자의 PFS 를 조사하기 위해 아래와 같은 일정으로 추적관찰을 시행한다.

6.9.2.2. **재발 시점 확인 혈액검사:** 베이스라인(수술 전), 화학요법 3 주기, 화학요법 6 주기, 마지막 항암치료 이후 초기 2 년동안은 3 개월(±1 개월) 마다 종양표지자 자료를 수집하고, 2 년 이후부터 3 년 동안은 6 개월(±1 개월)마다 종양표지자 자료를 수집한다. 유효성 평가는 질병의 진행이 확인된 시점에 추적 및 수집을 중단한다.
*질병특이적 종양표지자: CA125 를 임상적 필요에 따라 시행하고, 베이스 라인 및 유효성 평가 시점에 가장 가까운 데이터를 CRF 로 수집한다.

6.9.2.3. **재발 시점 확인 영상검사**: 베이스라인(수술 전), 화학요법 3 주기, 화학요법 6 주기, 마지막 항암치료 이후 초기 2 년동안은 3 개월(±1 개월) 마다 영상 자료를 수집하고, 2 년 이후부터 3 년 동안은 6 개월(±1 개월)마다 영상 자료를 수집한다. 유효성 평가는 질병의 진행이 확인된 시점에 추적 및 수집을 중단한다.
*종양평가 스캔은 임상적 필요에 따라 시행하고, 스캔 부위 역시 임상적 필요에 따라 시행하나, 복부와 골반 부위를 기본적으로 포함한다. CT 를 기반으로 하고, 필요 시 MRI, PET-CT 검사를 시행한다. 베이스라인 및 유효성 평가 시점에 가장 가까운 데이터를 CRF 로 수집한다.

6.9.2.4. **삶의 질 평가:** 수술 전, 수술 후 7 일 후, 항암화학요법 3 주기, 6 주기, 마지막 항암치료 이후 초기 1 년동안은 3 개월(±1 개월) 마다 환자의 삶의 질을 평가하고, 1 년 이후부터 4 년 동안은 12 개월(±1 개월)마다 환자의 삶의 질을 평가한다. 종양의 진행이 확인된 경우에는 QOL f/u 을 중단하고 환자의 생존 정보에 대해서 확인한다.

6.9.2.5. **PFS 추적관찰을 종료하는 경우:**

- 문서화된 질병의 진행
- 환자의 동의 철회
- Follow up loss
- 새로운 항암요법을 시작할 시 PFS 는 새로운 항암화학요법 시행 전 재발을 판정한 영상 검사 날짜로 수집하고, PFS 추적관찰을 종료한다.
- 질병의 진행 전 사망

6.9.2.6. **생존 추적관찰**

생존 추적관찰을 수술 후 보조항암화학요법의 치료 종료일 시점으로부터 5 년까지의 생존을 조사하며, PFS 추적관찰과 함께 시행한다. 생존 추적관찰은 PFS 추적관찰이 중단되더라도, 종양의

진행이 확인된 시점을 기준으로 1 년마다(window period: ±1 개월) 생존 여부를 확인 후 증례기록서에 작성한다. 수술일로부터 90 일 내의 사망 시, 사망원인의 조사가 포함된다.

6.9.2.7. **생존 추적관찰을 종료하는 경우**

- 환자의 사망
- 환자의 동의 철회
- Follow up loss

7. 임상시험 변수의 수집

7.1. 자료의 기록

자료 수집은 EMR 의 데이터를 사용하며, EMR 데이터로 획득이 어려운 부분은 Worksheet 를 사용하여 자료를 수집한다. 증례기록서에 수집된 자료를 기록해야 한다.

연구자는 완결된 CRF 에 최종 서명을 하여 완결된 CRF 의 사본을 연구기관에 보관한다.

7.2. 등록과 추적조사 시점의 자료 수집

국립암센터
NATIONAL CANCER CENTER

	Screening /Baseline	Surgery ±HIPEC	POD #1	POD #2	POD #4	POD #7	The 3rd Adjuvant Chemotherapy	Last Adjuvant Chemotherapy	#1 3Mo	#2 6Mo	#3 9Mo	#4 12Mo	#5 15Mo	#6 18Mo	#7 21Mo	#8 24Mo	#9 30Mo	#10 36Mo	#12 42Mo	#13 48Mo	#14 54Mo	#15 60Mo
Informed Consent	*																					
Eligibility	*																					
Medical History	*																					
Body Weight, Height	*																					
ECOG	*																					
Chest X-ray†	*																					
ECG†	*																					
Lab† CBC/Admin panel Electrolyte	*																					
Randomization‡		*																				
CT & Tumor Marker	*	*	*	*	*	*	*	*	*	*	*	*	*	*	*	*	*	*	*	*	*	*
QOL	*	*	*	*	*	*	*	*	*	*	*	*		*		*		*		*		*
Adverse Event¶	*	*	*	*	*	*	*	*														
Survival Status	*	*	*	*	*	*	*	*	*	*	*	*	*	*	*	*	*	*	*	*	*	*

†Preoperative lab, CT, tumor marker, chest x-ray, and ECG which was performed within 30 days is available.
‡The participant will be randomized if the patients have the residual tumor less than 1 cm after cytoreductive surgery.
†Postoperative lab could be selectively evaluated based on the patients's status and operation type.
¶Adverse event will be evalated from the time of randomication to the 6 weeks after the last adjuvant chemotherapy.

7.2.1. 등록/스크리닝 절차

임상시험 대상자는 수술 전에 문서 동의를 취득하며, 본 임상시험에서 수행할 검사와 평가 일정은 본 장과 표 1 에 기술하였다.

- 생년월일, 이니셜, 성별, 나이
- 이전의 항암요법과 방사선 요법을 포함한 병력 및 수술 이력
- 신보조항암치료 유무
- 신체검사, ECOG 활동도 상태, 활력징후, ECG
- 혈액학, 임상화학, 요분석, 종양표지자검사(CA125)
- BRCA 1/2 변이 상태 정보수집(검사가 기 시행된 경우)
- 종양 평가(CT, PET-CT. or MRI)
- 환자 삶에 질에 대한 베이스라인 점수를 확보한다. (EORTC QOL-C30, EORTC QOL_OV28, MDASI)

7.2.2. 임상시험 중의 평가

환자는 수술 후 연구에서 정하는 일정에 따라 검사를 시행할 것이다. (표 1 참고)

- 문진, 신체검사: 수술 전, 매 환자 조사 시점마다 확인하며, 자료를 CRF 에 수집할 필요는 없으나, 유의한 이상반응이 있는 경우 CRF 이상반응으로 수집되어야 한다.
- ECOG 활동도:
  - Single Arm: 수술 전, HIPEC 투약 후 24 시간, 48 시간, 96 시간, 7 일 시점에 수집한다
  - Two Arm: 수술 전 확인하여 수집한다
- 활력징후:
  - Single Arm: 수술 전, HIPEC 투약 후 24 시간, 48 시간, 96 시간, 7 일 시점에 시행하되, 유의한 이상이 있는 경우 이상반응으로 CRF 에 수집한다. 신장과 체중은 베이스라인 시점에만 확인한다.
  - Two Arm: 수술 전, HIPEC 투약 후 POD 1, 2, 4, 7 일(±1 일) 시점에 시행하되, 유의한 이상이 있는 경우 이상반응으로 CRF 에 수집한다. 신장과 체중은 베이스라인 시점에만 확인한다.
- ECG: 수술 전에 시행한 결과 상 이상이 있는 경우 병력으로 수집한다.
- 혈액학/임상화학/소변검사: 안전성 혈액/임상화학/소변 검사는 임상적 필요에 따라 시행 될 것이다. 수술 전, HIPEC 투약 후 POD 1,2,4,7 일(±1 일) 시점에 시행된 검사결과는 CRF 에 수집한다. 안전성 데이터 수집 기간 동안, 환자가 수행한 혈액학 및 임상화학 자료는 CRF 에 수집되지 않으나, 안전성 데이터 수집 기간 동안에 유의한 이상이 있는 경우, 이상반응으로 CRF 에 수집한다.

- *BRCA1/2* 변이 상태: 대상자가 BRCA 검사를 시행한 경우, 시점에 관계없이 변이상태를 확인하여 CRF 에 수집한다.
- Amifostine 사용 여부를 수집한다.
- 유효성 평가 및 시점:

  베이스라인(수술 전), 마지막 항암치료 이후 초기 2 년동안은 3 개월(±1 개월) 마다, 2 년 이후부터 3 년 동안은 6 개월(±1 개월)마다 종양표지자, 종양평가 스캔 자료를 수집한다.

  질병특이적 종양표지자: CA125 를 임상적 필요에 따라 시행하고, 베이스 라인 및 유효성 평가 시점에 가장 가까운 데이터를 CRF 로 수집한다.

  ■ 종양평가 스캔은 임상적 필요에 따라 시행하고, 스캔 부위 역시 임상적 필요에 따라 시행하나, 복부와 골반 부위를 기본적으로 포함한다. CT 를 기반으로 하고, 필요 시 MRI, PET-CT 검사를 시행한다. 베이스라인 및 유효성 평가 시점에 가장 가까운 데이터를 CRF 로 수집한다.

- 안정성 평가 및 기간: CTCAE 4.03 에 따라 아래 기간 동안 이상반응을 수집한다.

  ■ Post OP Adverse Event: 무작위배정 시점으로부터 다음 항암 시작 전까지

  ■ Chemotherapy Adverse Event: 첫 항암화학요법 시작일로부터 마지막 항암화학요법 시행일 이후 6 주째 시점까지

- 수술명, 잔류종양, 종양부담평가(Tumor Burden with SOTOC), 수술 및 마취기록지 자료.
- 난소의 병기, 조직형, 분화도
- 수술 후 합병증(수혈 유무, 혈액/화학적 검사, 영상학적 검사) G/O 까지 소요일, 식이 진행까지의 소요일
- 배액관 삽입 여부, 보조항암화학요법까지의 기간, 사용된 항암화학요법의 종류
- 입원, ICU 이용, 입원 기간을 포함한 자원 사용을 수집한다.
- 환자 보고 결과 및 삶의 질 설문: 수술 전(Baseline), 수술 후 7 일(POD#7), 항암화학요법 3 주기(CTx#3), 6 주기(CTx#6), 마지막 항암화학요법일로부터 3 개월(3M), 6 개월(6M), 9 개월(9M), 1 년(1yr), 2 년(2yr), 3 년(3yr), 4 년(4yr), 5 년(5yr) 시점에, 직접 또는 전화로 생존 여부를 추적 조사할 때에 설문을 시행한다. 질병의 진행이 확인된 시점에 추적을 중단한다.

7.2.3. 추적관찰 및 생존 조사절차

- 질병 진행으로 인한 중단: 종양의 진행이 문서화된 경우, 종양평가 정보 수집 및 삶의 질 설문을 중단하고 사망일까지 생존정보만 추적 관찰을 진행한다. 생존 정보만 추적 관찰하는 경우, 종양의 진행이 확인된 시점을 기준으로 1 년(±1 개월) 마다 생존 여부를 확인하고 CRF 를 작성한다.
- Follow Up loss: 대상자가 본원 방문을 더 이상 진행하지 않으며, 대상자와 연락을 3 회 이상 시도하였으나 연결이 되지 않는 경우, Follow up loss 를 문서화하고, 마지막 Follow up 날짜를 생존 정보로 수집한다.
- 동의철회: 종양평가정보 수집 및 삶의 질 설문, 생존정보 조사 모두 추적을 중단한다.

7.2.4. 실험실 안전성 평가

임상적 필요에 따라, 혈액학적/생화학적/요분석을 시행하고, 임상적으로 유의한 결과값은 이상반응으로 CRF 수집한다.

8. 통계분석계획

8.1. 대상자 수 산정

8.1.1. Single Arm 대상자 수 산정: 총 대상자 수 48 명이며 환자 등재 기간은 3 년, 마지막 환자 등재 이후 총 추적기간은 2 년으로 한다.

본 연구는 single-arm 2 상 연구로 아직 복강내고온항암화학요법 후 정맥 항암화학요법을 받은 환자에서의 2 년 무병생존율에 대한 정보가 없으므로 표본 수 산출은 어떤 통계적인 검정에 기반되지 않는다. 매년 16 명의 환자의 참여가 가능할 것을 고려하여 3 년간의 accrual period 뒤에 48 명의 대상자를 포함할 것이다.

8.1.2. Two Arm 대상자 수 산정: 총 대상자 수 184 명이며 환자 등재 기간은 6 년. 대조군에서의 중앙생존율 18 개월, Hazard ratio 0.75 으로 가정하였을 때(20% type I error rate, one-sided log-rank test, 통계적 검정력=80%) follow-up 기간 동안 140 명의 progression event 가 요구된다. 통계분석 시 Design 상 Dropout rate 를 제외하고 생각할 때, Power 를 유지하기 위해 꼭 필요한 인원수는 162 명이다. 등재 후 병리결과 부적합[예: Early stage], 6 년간의 등재 기간, 마지막 등재 환자의 2 년 follow-up 기간 및 11%의 drop-out rate 를 고려하여 총 필요 환자수는 184 명이다. (Armstrong et al., NEJM 2006;354:34-43./Rubinstein et al., JCO 2005;23:7199-7206.)

8.2. 유효성 및 안전성 통계 분석 방법(Two Arm)

증례기록 서식에 따라 기록하고 통계 처리한다. 환자들의 기본적인 특성 및 중요 변수들은 기술통계적인 방법을 이용하여 요약한다. 범주형 변수의 경우 교차표 및 카이제곱 검정 또는 Fisher's Exact Test 를 이용하고 연속변수의 경우 평균, 표준편차 또는 중앙값 등으로 요약한다

8.2.1. 이환율과 사망률을 분석

8.2.1.1. 문합부 누출, 농양, 수술 후 출혈, 패혈증, 췌장염, 폐렴, 신경학적 합병증, 골수독성, 신독성 등의 합병증은 무작위배정시점으로부터 다음 항암 시작전까지, 그리고 첫 항암화학요법 시작일로부터 마지막 항암화학요법 시행일 이후 6 주째 시점까지 관찰한다.

8.2.1.2. 수술에 의한 사망은 수술일 기준으로 30 일 이내 사망했을 경우로 한다.

8.2.1.3. 합병증과 수술에 의한 사망여부는 빈도수로 제시하며 임상적인 가능한 예후인자에 따른 차이를 볼 경우 Fisher's exact test 를 이용하여 분석한다.

8.2.1.4. 생존기간은 무작위 배정 시점으로부터 최종 시점까지의 기간을 개월수로 나타내고, 무병 생존기간은 진단일로부터 질병의 증거가 다시 나타날 때까지의 기간을 개월수로 나타낸다. 생존확률 및 median survival time 은 Kaplan-Meier method 를 이용하여 추정하고, RCT 연구의 경우, 군간 생존률 비교는 log rank test 를 이용한다. 임상적으로 중요한 다른 변수들과 생존기간과의 연관성을 보기 위해서는 Cox proportional hazards model, 또는 exponential model 등을 이용하고, 효과크기로는 Hazard ratio 를 이용한다. 합병증의 유무는 빈도수로 나타내고 필요한 경우 Fisher's exact test 를 사용하여 빈도 분포의 차이를 검정한다.

8.2.2. 중요한 예후 인자의 평가

생존 및 안전성에 영향을 미칠 것으로 보이는 인자들 즉, 환자의 연령, 신보조항암치료 유무, 난소암의 병기, 조직형, 분화도, BRCA 변이정보, 수술 시 종양부담(SOTOC 평가), 수술 후 잔류 종양의 크기 등을 포함하여 수술명, 수술 시간, 수술 중 합병증, Amifostine 투약 여부, 수술 후 합병증(수혈 유무, 혈액/화학적 검사, 영상학적 검사) 중환자실 재원일수, 수술 후 재원일 수, G/O 까지 소요일, 식이진행까지의 소요일, 배액관삽입 여부, 보조항암화학요법까지의 기간, 사용된 항암화학요법의 종류, 혈전증과 같은 내과적 동반질환, 재발종양의 부위 및 크기, 1 차 치료 후 재발까지의 기간, 등의 예후 인자로서의 가치를 Cox's proportional hazard model 또는 exponential model 을 이용하여 추정한다. 통계적 유의성은 $p<0.05$ 인 경우로 한다.

8.2.3. 삶의 질

EORTC QOL-C30, EORTC QOL-OV28, MDASI 를 이용하여 평가하고, 3 분류로 (1.Functional scale 2.Global quality of life 3.Symptom scale) 평가한다. 각 분류마다, 매 평가할 때 마다 raw answer 에 대한 frequency 와 평균치 분석, 반복적 측정으로 인한 시간의 변화에 따른 삶의 질 변화 평가를 시행한다. 통계적 분석은 repeated ANCOVA (Analysis of Covariance), ANOVA for repeated measures, Mixed Effects model, GEE (Generalized Estimating Equation)를 이용한다.

8.2.4. 안전성

2009 년 출간된 Phase II 연구에서 HIPEC 과 관련된 안전성은 확인되었다. 본 임상연구에서는 CTCAE 4.03 기준에 따라서 side effect 를 평가 및 보고한다. 안전성에 대한 issues 는 필요시 독립적인 Data Monitoring Committee 와 IRB 의 의견을 듣고 결정이나 권고사항을 연구에 반영한다.

9. 자료관리

9.1. Source Document/Essential Document 의 관리

관련 국내법과 기관의 지침에 따라 관리한다.

9.2. 증례기록서(CRF)

CRF 는 Version 4.0 부터 Excel 로 이용하여 전자데이터로 관리한다. 이때 대상자 식별이 가능한 개인정보는 제외된다.

9.3. 자료의 폐기

통계 분석이 완료되고 기관 윤리위원회 결과 보고 후 3 년 동안 국립암센터 임상시험센터의 문서이관을 통하여 보관하며, 3 년이 경과 후 모든 연구자료는 폐기한다.

10. 윤리 및 규제관련 고려사항

10.1. 대상자의 보호

시험자는 대상자에게 본 임상시험에 대해 충분히 설명하고, 각 대상자가 본 시험에 참여할 것인가에 대해 대상자로부터 반드시 문서 동의를 받아야 한다. 동의는 동의서 양식에 대상자의 서명과 날짜를 기입함으로써 문서화된다. 시험자가 규정에 따라 엄격히 임상시험을 실시했음에도 불구하고 대상자가 본 임상시험에서 시행된 검사 및 수술적 처치와 인과론적인 관련이 있는 합병증의 발생으로 고통을 당하게 되면 이에 대한 합리적인 의학적 조치를 취하게 된다. 그러나 그에 따른 경제적인 보상은 시행하지 않는다.

11. 참고문헌

1. Shingleton HM, Kim RY. Treatment of cancer of the cervix, In: Gusberg SB, Female genital cancer, NY, Churchill Livingstone, 1988, 297-335.
2. DiSaia PJ, Creasman WT. Clinical Gynecologic Oncology, St. Louis, Mosby-Year Book, 1993, 111.
3. Pecorelli S, Beller U, Heintz APM, Benedett JL, Creasman WT, Pettersson F. Annual report on the results of treatment in gynecologic cancer. J Epi Biostat 2001; 6; 116.
4. McClay EF, Howell SB. A review: i.p. cisplatin in the management of patients with ovarian cancer. Gynecol Oncol 1990; 36: 1-6.

5. Albert DS, Liu PY, Hanningan EV, et al. Intraperitoneal cisplatin plus intravenous cyclophosphamide versus intravenous cisplatin plus intravenous cyclophosphamide for stage III ovarian cancer. N Eng J med 1996; 335: 1950-5.

6. Dahl O. Mechanism of thermal enhancement of chemotherapeutic cytotoxicity. In: Urano M, Douple E (eds) Hyperthermia and Oncology: Trecht 1994; 4: 9-28.

7. Vange NV, Goethem ARV, Zoetmulder FAN, Kaag MM, Vaart PJMV, Huinink WWTB, and Beijnen JH. Extensive cytoreductive surgery combined with intra-operative intraperitoneal perfusion with cisplatin under hyperthermic condition (OVHIPEC) in patients with recurrent ovarian cancer: a feasibility pilot. Eur j Sur Oncol 2000; 26: 663-668.

8. Sugarbaker PH, Kern K, Lack E. Malignant pseudomyxoma peritonei of colonic origin. Natural history and presentation of a curative approach to treatment. Dis Colon Rectum 1987; 30: 772-779.

9. Koga S, Hamazoe R, Maetra M, Shimizu N, Murakami A, Wakatsuki T. Prophylactic therapy for peritoneal recurrence of gastric cancer by continuous hyperthermic peritoneal perfusion with mitomycin C. Cancer 1988; 61: 232-7

10. Schneebaum S, Lange M, Arnold M, et al. I.P. hyperthermic perfusion for recurrent colorectal cancer, a feasibility study. Reg Cancer Treat 1992; 4: 277-281.

11. Scarabelli C, Gallo A, Carbone A: Secondary cytoreductive surgery for patients with recurrent epithelial ovarian carcinoma. Gynecol Oncol 2001;83:237-241

12. Janicke F, Holscher M, Kuhn W, et al: Radical surgical procedure improves survival time in patients with recurrent ovarian cancer. Cancer 1992;70:2129-2136

13. Eisenkop SM, Friedman RL, Spirtos NM: The role of secondary cytoreductive surgery in the treatment of patients with recurrent epithelial ovarian carcinoma. Cancer 2000;88:144-153

14. A.R. Munkarah, R.L. Coleman: Critical evaluation of secondary cytoreduction in recurrent ovarian cancer. Gyn Oncol 2004;95:273-280

15. Armstrong DK, Bundy B, Wenzel L, et al: Intraperitoneal cisplatin and paclitaxel in ovarian cancer. N Engl J Med 2006;354:34-43

16. Markman M, Bundy BN, Alberts DS, et al: Phase III trial of standard-dose intravenous cisplatin plus paclitaxel versus moderately high-dose carboplatin followed by intravenous paclitaxel and intraperitoneal cisplatin in small-volume stage III ovarian carcinoma: An intergroup study of the Gynecologic Oncology Group, Southwestern Oncology Group, and Eastern Cooperative Oncology Group. J Clin Oncol 2001 ;19:1001-1007

17. Hager ED, Dziambor H, Hohmann D, Muhe N, Strama H. Intraperitoneal hyperthermic perfusion chemotherapy of patients with chemotherapy-resistant peritoneal disseminated ovarian cancer. Int J Gynecol Cancer 2001;11(Suppl. 1):57–63.

18. Chatzigeorgiou K, Economou S, Chrysafis G, Dimasis A, Zafiriou G, Setzis K, et al. Treatment of recurrent epithelial ovarian cancer with secondary cytoreduction and continuous intraoperative intraperitoneal hyperthermic chemoperfusion (CIHIPECP). Zentralbl Gynakol 2003; 125:424–9.

19. Piso P, Dahlke MH, Loss M, Schlitt HJ. Cytoreductive surgery and hyperthermic intraperitoneal chemotherapy in peritoneal carcinomatosis from ovarian cancer. World J Surg Oncol 2004;2:21.

20. Ryu KS, Kim JH, Ko HS, Kim JW, Ahn WS, Park YG, et al. Effects of intraperitoneal hyperthermic chemotherapy in ovarian cancer. Gynecol Oncol 2004;94:325–32.

21. Zanon C, Clara R, Chiappino I, Bortolini M, Cornaglia S, Simone P, et al. Cytoreductive surgery and intraperitoneal chemohyperthermia for recurrent peritoneal carcinomatosis from ovarian cancer. World J Surg 2004;28:1040–5.

22. Gori J, Castano R, Toziano M, Habich D, Staringer J, De Quiros DG, et al. Intraperitoneal hyperthermic chemotherapy in ovarian cancer. Int J Gynecol Cancer 2005;15:233–9.

23. 박상윤. 난소암의 복막내항암화학요법 (IPC; intraperitoneal chemotherapy) 과 수술중온열항암화학요법 (HIPEC; hyperthermic intraperitoneal chemotherapy). 대한 복막암 연구회 2006 년 9 월 2 일, 무주

24. 임명철, 배재만, 임소이, 박정열, 강석범, 서상수, 박상윤. Feasibility of intraoperative intraperitoneal hyperthermic chemotherapy for peudomyxoma peritonei and diffuse malignant peritoneal mesothelioma. 대한산부인과 학회 2006 년 9 월 29 일~30 일, 서울 그랜드 힐튼 호텔

25. Kusamura S, Younan R, Baratti D, Costanzo P, Favaro M, Gavazzi C, Deraco M. Cytoreductive surgery followed by intraperitoneal hyperthermic perfusion: analysis of morbidity and mortality in 209 peritoneal surface malignancies treated with closed abdomen technique. Cancer. 2006 Mar 1;106(5):1144-53.

26. Rossi CR, Foletto M, Mocellin S, Pilati P, De SM, Deraco M, Cavaliere F, Palatini P, Guasti F, Scalerta R, Lise M. Hyperthermic intraoperative intraperitoneal chemotherapy with cisplatin and doxorubicin in patients who undergo cytoreductive surgery for peritoneal carcinomatosis and sarcomatosis: phase I study. Cancer. 2002 Jan 15;94(2):492-9.

27. Walker JL, Armstrong DK, Huang HQ, Fowler J, Webster K, Burger RA, Clarke-Pearson D. Intraperitoneal catheter outcomes in a phase III trial of intravenous versus intraperitoneal chemotherapy in optimal stage III ovarian and primary peritoneal cancer: a Gynecologic Oncology Group Study. Gynecol Oncol. 2006 Jan;100(1):27-32.

28. Panteix G, Beaujard A, Garbit F, Chaduiron-Faye C, Guillaumont M, Gilly F, Baltassat P, Bressolle F. Population pharmacokinetics of cisplatin in patients with advanced ovarian cancer during intraperitoneal hyperthermia chemotherapy. Anticancer Res. 2002 Mar-Apr;22(2B):1329-36.

29. Schmid K, Boettcher MI, Pelz JO, Meyer T, Korinth G, Angerer J, Drexler H. Investigations on safety of hyperthermic intraoperative intraperitoneal chemotherapy (HIPEC) with Mitomycin C. Eur J Surg Oncol. 2006 Dec;32(10):1222-1225.

30. Gonzalez-Bayon L, Gonzalez-Moreno S, Ortega-Perez G. Safety considerations for operating room personnel during hyperthermic intraoperative intraperitoneal chemotherapy perfusion. Eur J Surg Oncol. 2006 Aug;32(6):619-24.

31. Lim MC, Kang S, Choi J, Song YJ, Park S, Seo SS, Park SY. Hyperthermic intraperitoneal chemotherapy after extensive cytoreductive surgery in patients with primary advanced epithelial ovarian cancer: interim analysis of a phase II study. Ann Surg Oncol. 2009 Apr;16(4):993-1000. Epub 2009 Jan 24. PMID:19169758

32. WJ Van Driel, SN Koole, K Sikorska, et al. Hyperthemic Intraperitoneal Chemotherapy in Ovarian Cancer. N Engl J Med 2018 Jan 18; 378:230-240.

APPENDIX

Text in this APPENDIX taken directly from the references is written in italic font.

1. SOTOC form

- Synoptic Operative Template for Ovarian Cancer (SOTOC) form to describe preoperative and postoperative tumor burden. The largest diameter of the tumor (mm) per each area will be described at the SOTOC, as parts of operation record of Electronic Medical Record.

Site	Preoperative Tumor size (mm)	Postoperative Tumor size (mm)
① Omentum		
② Left upper quadrant		
③ Epigastric		
④ Right upper quadrant		
⑤ Colon		
⑥ Small bowel		
⑦ Para-colic gutters		
⑧ Pelvis		
⑨ Lymph node		
⑩ Others		

APPENDIX

2. RECIST Criteria

2.1. Evaluation of target lesions

- *Complete Response (CR): Disappearance of all target lesions. Any pathological lymph nodes (whether target or non-target) must have reduction in short axis to <10mm.*
- *Partial Response (PR): At least a 30% decrease in the sum of diameters of target lesions, taking as reference the baseline sum diameters.*
- *Progressive Disease (PD): At least a 20% increase in the sum of diameters of target lesions, taking as reference the smallest sum on study (this includes the baseline sum if that is the smallest on study). In addition to the relative increase of 20%, the sum must also demonstrate an absolute increase of at least 5 mm. (Note: the appearance of one or more new lesions is also considered progression).*
- *Stable Disease (SD): Neither sufficient shrinkage to qualify for PR nor sufficient increase to qualify for PD, taking as reference the smallest sum diameters while on study.*

2.2 Evaluation of non-target lesions

- *Complete Response (CR): Disappearance of all non-target lesions and normalisation of tumour marker level. All lymph nodes must be non-pathological in size (<10 mm short axis).*
- *Non-CR/Non-PD: Persistence of one or more non-target lesion(s) and/or maintenance of tumour marker level above the normal limits.*
- *Progressive Disease (PD): Unequivocal progression (see comments below) of existing non-target lesions. (Note: the appearance of one or more new lesions is also considered progression).*

REFERENCE

E.A. Eisenhauer; P. Therasse; J. Bogaerts; L.H. Schwartz; D. Sargent; R. Ford; J. Dancey; S. Arbuck; S. Gwyther; M. Mooney; L. Rubinstein; L. Shankar; L. Dodd; R. Kaplan; D. Lacombe; J. Verweij, "New response evaluation criteria in solid tumours: Revised RECIST guideline (version 1.1). European Journal of Cancer 2009; 45: 228–247.

APPENDIX

3. The GCIG CA125 criteria for Disease Progression

- *Progression or recurrence based on serum CA 125 levels will be defined on the basis of a progressive serial elevation of serum CA 125 according to the following criteria:*

  A. Patients with elevated CA-125 pretreatment and normalization of CA-125 must show evidence of CA-125 greater than, or equal to, 2 times the upper limit of the reference range on 2 occasions at least 1 week apart or

  B. Patients with elevated CA-125 before treatment, which never normalizes, must show evidence of CA-125 greater than, or equal to, 2 times the nadir value on 2 occasions at least 1 week apart or

  C. Patients with CA-125 in the reference range before treatment must show evidence of CA-125 greater than, or equal to, 2 times the upper limit of the reference range on 2 occasions at least 1 week apart.

REFERENCE

1) *E.A. Eisenhauer; P. Therasse; J. Bogaerts; L.H. Schwartz; D. Sargent; R. Ford; J. Dancey; S. Arbuck; S. Gwyther; M. Mooney; L. Rubinstein; L. Shankar; L. Dodd; R. Kaplan; D. Lacombe; J. Verweij, "New response evaluation criteria in solid tumours: Revised RECIST guideline (version 1.1). European Journal of Cancer 2009; 45: 228–247.*

2) *Rustin GJ, Vergote I, Eisenhauer E, et al. Definitions for response and progression in ovarian cancer clinical trials incorporating RECIST 1.1 and CA 125 agreed by the Gynecological Cancer Intergroup (GCIG). International journal of gynecological cancer: official journal of the International Gynecological Cancer Society 2011; 21: 419-23.*

APPENDIX

4. EORTC-C30, EORTC-OV28 and MDASI (Korean version)

Subject No :
Initial :

HIPEC NCCCTS-06-222
QOL

Visit:_______________
Date:_______________

EORTC QLQ-C30(3 판)

귀하와 귀하의 건강 상태에 대하여 몇가지 조사하고자 합니다. 모든 질문에 대한 응답은 귀하 스스로 해주시고, 각 문항마다 귀하와 가장 가깝다고 생각되는 부분에 동그라미 표시를 해주시기 바랍니다. 본 질의서에 게재되어 있는 질문에는 정답이나 오답이 정해져 있지 않으며 귀하가 제공하는 모든 정보에 대한 비밀은 엄격히 보호됩니다.

		전혀 아니다	약간 그렇다	꽤 그렇다	매우 그렇다
1	무거운 쇼핑 백이나 가방을 옮길 때처럼 힘을 쓰는 일을 할 때 곤란을 느끼십니까?	1	2	3	4
2	오래 걷는 것이 힘이 드십니까?	1	2	3	4
3	밖에서 잠깐 걷는 것이 힘이 드십니까?	1	2	3	4
4	낮 시간 중에 자리(침대)에 눕거나 의자에 기대고 싶습니까?	1	2	3	4
5	식사 도중 혹은 옷을 입는 동안, 세면을 할 때나 화장실을 이용할 때 누군가의 도움이 필요합니까?	1	2	3	4

* 지난 한 주를 기준으로 답변하여 주십시오.		전혀 아니다	약간 그렇다	꽤 그렇다	매우 그렇다
6	일을 하거나 기타 일상생활을 영위하는데 한계를 느낀 적이 있습니까?	1	2	3	4
7	취미생활이나 여가활동을 하는데 있어 한계를 느낀 적이 있습니까?	1	2	3	4
8	숨이 가쁜 적이 있습니까?	1	2	3	4
9	통증을 느껴 본 적이 있습니까?	1	2	3	4
10	휴식이 필요하다고 생각한 적이 있습니까?	1	2	3	4
11	숙면을 취하는데 곤란을 느낀 적이 있습니까?	1	2	3	4
12	몸이 허하다고 느낀 적이 있습니까?	1	2	3	4
13	식욕이 감퇴하셨습니까?	1	2	3	4
14	속이 메스꺼운 적이 있습니까?	1	2	3	4
15	구토를 하신 적이 있습니까?	1	2	3	4

* 지난 한 주를 기준으로 답변하여 주십시오.		전혀 아니다	약간 그렇다	꽤 그렇다	매우 그렇다
16	변비 증세를 경험한 적이 있습니까?	1	2	3	4
17	설사를 한 적이 있습니까?	1	2	3	4
18	피로를 느끼셨습니까?	1	2	3	4
19	통증으로 인해 일상생활을 영위하는데 지장을 받은 경험이 있습니까?	1	2	3	4
20	신문을 읽거나 텔레비전을 시청할 때 집중하는 데 곤란을 겪은 경험이 있습니까?	1	2	3	4
21	긴장감을 느끼셨습니까?	1	2	3	4
22	걱정에 시달리셨습니까?	1	2	3	4
23	짜증을 느끼셨습니까?	1	2	3	4
24	우울함을 느끼셨습니까?	1	2	3	4
25	기억력 감퇴를 느끼셨습니까?	1	2	3	4
26	귀하의 건강상태나 의약치료가 귀하의 가정 생활에 어떤 곤란을 야기 했습니까?	1	2	3	4

Subject No :<br>Initial :	**HIPEC NCCCTS-06-222**<br>**QOL**	Visit:______________<br>Date:______________

27	귀하의 건강상태나 의약치료가 귀하의 사회 생활에 어떤 곤란을 야기 했습니까?	1	2	3	4
28	귀하의 건강상태나 의약치료로 인하여 경제적인 어려움을 겪으셨습니까?	1	2	3	4

*** 다음 문항을 읽고 1에서 7까지 번호 중 귀하와 가장 가깝다고 생각되는 번호에 동그라미 표시를 해 주시기 바랍니다.**

29. 지난 한 주간의 전반적인 귀하의 건강 상태를 평가하신다면 다음 중 어디에 해당합니까?

1	2	3	4	5	6	7
매우 나쁨						아주 좋음

30. 지난 한 주간의 전반적인 귀하의 삶의 질을 평가하신다면 다음 중 어디에 해당합니까?

1	2	3	4	5	6	7
매우 나쁨						아주 좋음

© Copyright 1995 EORTC Study Group on Quality of Life. All rights reserved. Version 3.0

EORTC QLQ –OV28

환자들은 때때로 다음과 같은 증상이나 문제들을 호소합니다. 지난 일주일동안 이 증상들이나 문제들을 어느 정도 경험했는지 해당사항에 동그라미 표시를 해주시기를 바랍니다.

**지난 일주일 동안에:**		**전혀 아니다**	**약간 그렇다**	**꽤 그렇다**	**매우 그렇다**
31	배가 아픈 적이 있습니까?	1	2	3	4
32	배에 가스가 찬 느낌이 있었습니까?	1	2	3	4
33	옷이 너무 끼이는 문제점이 있었습니까?	1	2	3	4
34	질병 또는 치료의 결과로 배변습관에 변화를 경험했습니까?	1	2	3	4
35	방구가 나오거나 가스가 차거나 헛배가 불러 곤란했습니까?	1	2	3	4
36	먹기 시작한 후, 너무 빨리 배가 부른 적이 있습니까?	1	2	3	4
37	소화불량이나 가슴앓이가 있었습니까?	1	2	3	4
38	머리카락이 조금이라도 빠졌습니까?	1	2	3	4
39	머리카락이 조금이라도 빠졌던 분만 답하세요.<br>머리카락이 빠진 것에 대해 속상한 적이 있습니까?	1	2	3	4
40	음식이나 음료 맛이 평소와는 다르게 느껴진 적이 있습니까?	1	2	3	4
41	손이나 발이 쑤신 적이 있습니까?	1	2	3	4
42	손가락이나 발가락이 마비된 적이 있었습니까?	1	2	3	4
43	팔이나 다리가 약해진 느낌이 들었습니까?	1	2	3	4
44	근육이나 관절에 통증이 있었습니까?	1	2	3	4
45	청력에 문제가 있었습니까?	1	2	3	4
46	자주 소변을 보셨습니까?	1	2	3	4
47	피부에 문제가 있은 적이 있습니까?<br>(예를 들면 가렵거나, 피부건조)	1	2	3	4
48	얼굴이 화끈 달아 오른 적이 있습니까?	1	2	3	4
49	밤에 식은 땀이 났습니까?	1	2	3	4

793

Subject No :
Initial :

HIPEC NCCCTS-06-222
QOL

Visit:_______________
Date:_______________

**지난 일주일 동안에:**	**전혀 아니다**	**약간 그렇다**	**꽤 그렇다**	**매우 그렇다**
50 질병이나 치료로 인해 자신의 신체적인 매력이 전보다 못하다고 느낀 적이 있습니까?	1	2	3	4
51 자신의 신체에 대해 불만스러운 적이 있습니까?	1	2	3	4
52 질병이 얼마나 부담이 됩니까?	1	2	3	4
53 치료가 얼마나 부담이 됩니까?	1	2	3	4
54 앞으로의 건강에 대해 걱정을 했습니까?	1	2	3	4

**지난 4주 동안에:**	**전혀 아니다**	**약간 그렇다**	**꽤 그렇다**	**매우 그렇다**
55 성생활에 대해 어느 정도의 흥미를 느끼셨습니까?	1	2	3	4
56 성생활에 어느 정도나 활발하셨습니까?	1	2	3	4
**당신이 성적으로 적극적인 경우에만 다음의 두 질문에 대답해주십시오.**				
57 성생활이 어느 정도나 즐거웠습니까?	1	2	3	4
58 성행위를 하는 동안 질이 건조했습니까?	1	2	3	4

M.D.Anderson 증상 조사지 중심항목

1 부. 당신의 증상이 얼마나 심합니까?

암 환자들은 흔히 자신이 앓고 있는 질병 때문에, 그리고 그 질병을 치료하는 과정에서 여러가지 증상을 느낍니다. 당신이 **지난 24시간 동안** 다음의 증상들을 어떻게 느꼈는지 그 정도를 표현해 주십시오. 각각 항목에 대해 0(증상이 없음)부터 10(상상할 수 없을 정도로 심한 증상)까지 아래의 동그라미를 채워 주십시오.

	없음 0	1	2	3	4	5	6	7	8	9	상상할 수 없을 정도로 심함 10
1. 가장 심했을 때의 통증	○	○	○	○	○	○	○	○	○	○	○
2. 가장 심했을 때의 피로	○	○	○	○	○	○	○	○	○	○	○
3. 가장 심했을 때의 메스꺼움	○	○	○	○	○	○	○	○	○	○	○
4. 가장 심했을 때의 수면 장애	○	○	○	○	○	○	○	○	○	○	○
5. 가장 심했을 때의 괴로움(당혹감)	○	○	○	○	○	○	○	○	○	○	○
6. 가장 심했을 때의 숨가쁨	○	○	○	○	○	○	○	○	○	○	○
7. 가장 심했을 때의 건망증	○	○	○	○	○	○	○	○	○	○	○

Subject No :
Initial :

HIPEC NCCCTS-06-222
QOL

Visit:______________
Date:______________

	없음 0	1	2	3	4	5	6	7	8	9	상상할 수 없을 정도로 심함 10
8. 가장 심했을 때의 식욕부진	○	○	○	○	○	○	○	○	○	○	○
9. 가장 심했을 때의 졸음	○	○	○	○	○	○	○	○	○	○	○
10. 가장 심했을 때의 입이 마른 느낌	○	○	○	○	○	○	○	○	○	○	○
11. 가장 심했을 때의 슬픔	○	○	○	○	○	○	○	○	○	○	○
12. 가장 심했을 때의 구토	○	○	○	○	○	○	○	○	○	○	○
13. 가장 심했을 때의 무감각 하거나 저린 느낌	○	○	○	○	○	○	○	○	○	○	○

2 부. 당신의 증상들이 삶에 얼마나 지장을 주고 있습니까?

증상들은 흔히 우리들의 기분이나 일상생활에 여러 가지 지장을 줍니다. **<u>지난 24시간 동안</u>** 그런 증상들이 다음 항목들에 얼마나 지장을 주었습니까?

	지장을 주지 않음 0	1	2	3	4	5	6	7	8	9	완전히 지장을 줌 10
14. 일반적인 활동	○	○	○	○	○	○	○	○	○	○	○
15. 기분	○	○	○	○	○	○	○	○	○	○	○
16. 일(집안일 포함)	○	○	○	○	○	○	○	○	○	○	○
17. 대인관계	○	○	○	○	○	○	○	○	○	○	○
18. 보행 능력	○	○	○	○	○	○	○	○	○	○	○
19. 인생을 즐김	○	○	○	○	○	○	○	○	○	○	○

설문에 참여해 주셔서 감사합니다

795

Subject No :
Initial :

HIPEC NCCCTS-06-222
QOL

Visit:______________
Date:______________

설문지 작성자

1 환자
2 보호자
3 연구관련자

설문지 완성 여부

☐ 완성됨 ☐ 완성되지 않음

만약 완성 되지 않았다면, 해당 번호에 표기해주십시오.

1 환자가 너무 아픈 경우
2 의료인 또는 간호사가 판단하기에, 환자가 너무 아프다고 생각되는 경우
3 환자가 불편하고 시간이 오래 걸린다고 생각하는 경우
4 환자가 사생활 침해라고 생각하는 경우
5 환자가 말/글을 이해할 수 없는 경우
6 질문지 배포에 실패한 경우
7 기타(자세히 적을 것)
8 잘모름(unknown)

796

Summary of protocol change			
Protocol version	Date of approval	Area of Change	Amendments
NCCCTS-06-222-1	February 22, 2010	Research title	P2, 5<br>The Phase II Study of Intraoperative Hyperthermic Intraperitoneal Chemotherapy Followed by Intravenous Chemotherapy in Patients with Ovarian Cancer
NCCCTS-06-222-1	February 22, 2010	Randomization flowchart	P11<br>Flowchart of randomization
NCCCTS-06-222-1	February 22, 2010	Study population (Protocol in Korean)	P9, 20<br>Two separate study cohorts were described in the protocol (n=214)<br>· n= 168 for primary ovarian cancer (control and HIPEC arm for the randomized trial)<br>· n= 46 for recurrent ovarian cancer (single arm)
NCCCTS-06-222-1	February 22, 2010	Study objective (Protocol in Korean)	P5, 16<br>Primary objectives:<br>· To assess a 2-year progression-free survival outcome after intraoperative Hyperthermic Intraperitoneal Chemotherapy (HIPEC) vs. no HIPEC followed by intravenous chemotherapy in patients with primary advanced ovarian cancer.<br>Secondary objectives:<br>· To assess 3-year overall survival outcome and complication after intraoperative HIPEC vs. no HIPEC followed by intravenous chemotherapy in patients with primary advanced ovarian cancer.<br>· To assess the quality of life after intraoperative HIPEC vs. no HIPEC followed by intravenous chemotherapy in patients with primary advanced ovarian cancer.

NCCCTS-06-222-1	February 22, 2010	Inclusion Criteria (Protocol in Korean)	P7, 21<br>1. Disease status<br>A. Primary ovarian, tubal, and primary peritoneal cancer with stage III or more or,<br>B. Recurrent ovarian, tubal, and primary peritoneal cancer with a disease-free interval of 6 months or more<br>2. Residual tumor <1cm after completion of cytoreductive surgery<br>2. Age < 75 year<br>3. Expected survival> 3 months<br>4. Performance status: ECOG 0-1<br>5. Adequate bone marrow function<br>Hb≥8 g/dl (After correction in case of iron deficient anemia)<br>WBC≥3,000/mm3<br>Platelet≥100,000/mm3<br>6. Adequate renal function, Creatinine≤1.5 mg/dl<br>7. Adequate hepatic function, Bilirubin≤1.5 mg/dl and AST and ALT≤80 IU/L<br>8. Optimal cardiopulmonary function for surgery<br>9. Voluntary participation after getting written informed consent.
NCCCTS-06-222-1	February 22, 2010	Exclusion Criteria (Protocol in Korean)	P7, 8, 21, 22<br>1. Unresectable extraperitoneal metastasis (brain, bone, lung parenchyma, or lymph node)<br>2. Suboptimal debulking (residual tumor ≥ 1cm)<br>3. Previous history of other malignancies (except excision of skin cancer)<br>4. Serious heart disease or renal failure<br>5. Serious cardiopulmonary insufficiency<br>6. Uncontrolled infection<br>7. Uncontrolled intercurrent disease<br>8. Psychogenic disorder<br>9. Patients who are suitable candidates by legally<br>10. Pregnant or breast-feeding patients<br>11. Patients who are unsuitable candidates by doctor's decision

NCCCTS-06-222-1	February 22, 2010	Intervention of HIPEC (Protocol in Korean)	P17, 18<br>1) Location of the inflow and outflow catheter & thermometers<br>- Two inflow catheters and thermometers beneath pelvis.<br>- Two outflow catheters and thermometers beneath diaphragm.<br>- The abdominal wall was closed in layers with a water-tight fit.<br><br>2) Perfusion<br>- HIPEC Instrument: Belmont Hyperthermia pump approved by the Korea Food & Drug Administration<br>- Flow rate: 1L/min<br>- Manual shaking of the patients for even distribution of chemotherapeutic agents during 90 min<br><br>3) Temperature<br>- Check core temperature using a nasopharyngeal-temperature probe<br>- Perfusion of 0.9% saline solution with a temperature of 41.5 ℃<br>- Keep the core temperature not exceed 39 ℃ with 5 minutes interval
NCCCTS-06-222-1	February 22, 2010	Intervention (Protocol in Korean)	P18<br>If the intraperitoneal temperature is maintained above 41.5 ℃, cisplatin (75 mg/m^2) will be perfused for 90 minutes.
NCCCTS-06-222-1	February 22, 2010	Sample size calculation (Protocol in Korean)	P9, 24<br>Total number of subjects: 168 for primary ovarian cancer<br>- Median survival times are 18 months for control group<br>- A hazard ratio (HR) of 1.6.<br>- Type 1 error=0.05, 2 sided test, 80% power<br>- Required sample size for each arm, n=79<br>- Dropout rate, 5%<br>- Total sample size, n=168
NCCCTS-06-222-1	February 22, 2010	Evaluation (Protocol in Korean)	P9, 22, 23<br>- Tumor response, RECIST<br>- Toxicity, CTCAE 4.0 (Typo, described as 3.0 in Korean)<br>- Quality of life, EORTC-C30 and OV28 and MDASI

NCCCTS-06-222-2	May 17, 2010	Evaluation (Protocol in Korean)	P4, 5, 6, 8, 11, 12, 13, 14, 19, 20, 23<br>Removal of the content related to recurrent ovarian cancer
NCCCTS-06-222-3	June 16, 2010	Evaluation (Protocol in Korean)	P8<br>Addition of period of toxicity evaluation related to HIPEC<br>Postoperative 4 weeks (28 days)
NCCCTS-06-222-4	August 3, 2010	Research title	P1,4<br>Phase II Random study: Hyperthermic Intraperitoneal Chemotherapy (HIPEC) vs no HIPEC during the surgical management of primary epithelial ovarian cancer
NCCCTS-06-222-5	October 27, 2010	ICF (Protocol in Korean)	P4<br>Informed consent from (ICF version 3.0)<br>Addition for the risk of increased serum creatine because of cisplatin toxicity
NCCCTS-06-222-6	November 19, 2010	ICF (Protocol in Korean)	P4<br>Informed consent from (ICF version 3.1)<br>Addition for the risk of disseminated intravascular coagulopathy
NCCCTS-06-222-7	March 25, 2013	Institution (Protocol in Korean)	P9<br>Addition of Institution, Ajou University Hospital
NCCCTS-06-222-8	June 13, 2013	Exclusion Criteria (Protocol in Korean)	P6, 19<br>Removal of the thyroid cancer from the exclusion criteria<br>P7, 19<br>Addition of "no cancer tissue confirmed by frozen section" during interval cytoreductive surgery after neoadjuvant chemotherapy
NCCCTS-06-222-8	June 13, 2013	Accrual period (Protocol in Korean)	P8, 18<br>Accrual period: 6 years, Minimum Follow up period: 2 years (Total Period: 8 years)
NCCCTS-06-222-8	June 13, 2013	Sample size calculation (Protocol in Korean)	P8, 18, 21, 22<br>Total number of subjects: 170 for primary ovarian cancer<br>- Median survival times are 18 months for control group<br>- A hazard ratio (HR) of 1.33.<br>- Type 1 error=0.2, 1 sided test, 80% power<br>- Required sample size for each arm, n=85<br>- Dropout rate, 5%

			- Accrual period: 6 years and minimal follow up period: 2 year<br>- Total sample size, n=170
e-version 3.41	July 3, 2014	Research title	P1, 4<br>The Phase II Study of Intraoperative Hyperthermic Intraperitoneal Chemotherapy Followed by Intravenous Chemotherapy in Patients with Advanced Epithelial Ovarian Cancer
e-version 3.5	July 23, 2014	Randomization (Protocol in Korean)	P8, 18<br>Randomization<br>- The participants who are suitable for the inclusion and exclusion criteria will be randomized in the control or HIPEC group, 1:1 fashion by an independent person.
e-version 3.6	May 18, 2015	Preparation and procedure of HIPEC (Protocol in Korean)	P8, 18<br>Normal saline 1.5L will be infused for 12 hours before surgery to reduce the renal toxicity related cisplatin.<br>P8, 19<br>Temperature of infused fluid: 41.5→ 41-42 ℃<br>P8, 20<br>Use of Ametox (Sodium thiosulfate) in case of HIPEC arm<br>4g/m^2 before HIPEC<br>12g/m^2 during and after HIPEC for 6 hours<br>P20<br>Storage and management of Hyperthermia pump
e-version 3.7	June 12, 2015	Preparation of HIPEC (Protocol in Korean)	P8, 18<br>Type of fluid and infuse rate could be individualized based on the patient's physical status for the participant safety.
e-version 3.9	November 30, 2015	Sample size calculation (Protocol in Korean)	P10, 30<br>Total number of subjects: 184 for primary ovarian cancer<br>- Median survival times are 18 months for control group<br>- A hazard ratio (HR) of 1.33.<br>- Type 1 error=0.2, 1 sided test, 80% power<br>- Required sample size for each arm, n=92<br>- Dropout rate, 12%

			- Accrual period: 6 years, minimal follow up period: 2 years<br>- Total sample size, n=184
e-version 4.0	December 15, 2015	Exclusion criteria	P30<br>Withdrawal criteria<br>- Violation of inclusion or exclusion criteria<br>- Expected direct toxicity from the HIPEC
e-version 4.2	December 22, 2017	Study period (Protocol in Korean)	P10<br>Extension of study period to Dec 31, 2018
e-version 4.4	August 23, 2018	Study period (Protocol in Korean)	P10<br>Extension of the study period to Dec 31, 2023
e-version 4.5	January 3, 2019	Definition and collected variables (Protocol in Korean)	P10-11<br>· Clarification of collected variables and definition<br><br>P10<br>· Primary objectives<br>- Progression-free survival: from the date of diagnosis to the time of recurrence or death<br>· Secondary objectives<br>- The complication and 3-year overall survival<br>- Overall survival: from the date of diagnosis to the time of death<br><br>P 11<br>· Evaluation of the safety and adverse event: using CTCAE 4.03<br>- Postoperative Adverse Event: from postoperative day 1 to discharge day<br>- Convalescent Adverse Event: from the date of discharge to the first chemotherapy<br>- Chemotherapy Adverse Event: from the date of first chemotherapy to the 21 days after the last chemotherapy<br>· Exploratory variables (Neoadjuvant chemotherapy, adjuvant chemotherapy, information of BRCA, tumor burden estimated Peritoneal Carcinomatosis Index, Use of Ametox, insertion of drains

			P18<br>· Window period: 6 months to 1 month<br>· Reason for mortality should be investigated and described in case of mortality within the postoperative 90 days.<br><br>P23<br>· Specification of survival follow up,<br>- Death, withdrawal, or interruption of follow up
e-version 5.0	March 6, 2020	Research title	P1(P31)<br>The Randomized trial of Intraoperative Hyperthermic Intraperitoneal Chemotherapy Followed by Intravenous Chemotherapy in patients with Advanced Epithelial Ovarian Cancer
e-version 5.0	March 6, 2020	Affiliation	P2(P31)<br>Change of affiliation<br>- Center for Gynecologic Cancer
e-version 5.0	March 6, 2020	Study objective (Protocol in Korean)	P19<br>이차평가변수? – Overall Survival (OS)는 무작위 배정일로부터 사망일까지의 시간(Month)로 정의한다.<br>- RFS, DFS 내용<br>- QOL: 5 년

e-version 5.0	March 6, 2020	Abbreviation	P4(P33)<br>Addition of the abbreviation<br>- SOTOC, Synoptic operative template for ovarian cancer
e-version 5.0	March 6, 2020	Abstract	P5-14(P34-44)<br>Addition of English abstract
e-version 5.0	March 6, 2020	Evaluation of toxicity	P19, 30(P48, 60)<br>Evaluation of toxicity till 6 weeks after the last chemotherapy
e-version 5.0	March 6, 2020	Regimen of chemotherapy	P25(P55)<br>Addition of available treatment regimen for recurrent ovarian cancer<br>Drugs which can use for recurrent disease in daily clinical practice are updated.
e-version 5.0	March 6, 2020	Appendix	P 36-43(P66-73)<br>Addition of Appendix for Synoptic operative template for ovarian cancer (SOTOC), Response Evaluation Criteria in Solid Tumors (RECIST) criteria, CA125 criteria which used for this trial<br>Addition of Statistical Analysis Plan (SAP)
e-version 5.1	March 16, 2020	Sample size calculation	P15(P44)<br>We assumed that the median survival times are 1.5 year for control group and 2.0 year for treatment group, that is a hazard ratio (HR) of 0.75.<br>A log-rank test with a total sample size of 184 subjects (per each group 92 subjects) achieves 82.3% power at a one-sided 0.20 significance level to detect an HR of 0.75. We additionally calculated that a log-rank test with the sample size of 184 subjects achieves 68.8% power at one-sided 0.1 significance level to detect a HR of 0.75
Abbreviation: HIPEC, hyperthermic intraperitoneal chemotherapy; ICF, informed consent form,			
*The platform of the National Cancer Center Institutional Review Board for this trial was changed to the internet system from July 2014.			

Statistical Analysis Plan

(Version 1.1, Date: Mar 09, 2020)

I. Introduction

This statistical analysis plan describes the statistical methods to be performed for the analysis of data collected from a randomized clinical trial (National Cancer Center IRB protocol No. NCCCTS 06-222) “The Randomized trial of Intraoperative Hyperthermic Intraperitoneal Chemotherapy Followed by Intravenous Chemotherapy in patients with Advanced Epithelial Ovarian Cancer”.

The statistical analysis plan describes the details of sample size calculation and statistical methods for analyses of primary objective, secondary objective, toxicity and quality of life.

Note: Text in this statistical analysis plan taken directly from the study protocol is written in italic font.

II. Objectives of the trial and endpoints

2.1 Primary objective

- *The primary objective of this study is to compare the progression-free survival between Surgery group vs. Surgery + HIPEC group. Progression-free survival is defined as the duration from the time of randomization to the disease progression, recurrence or death.*

2.2 Secondary objectives

- *To assess recurrence-free, disease-free or overall survival outcome after intraoperative HIPEC vs. no HIPEC followed by intravenous chemotherapy in patients with primary advanced ovarian cancer.*
- *To assess toxicity and quality of life after intraoperative HIPEC vs. no HIPEC followed by intravenous chemotherapy in patients with primary advanced ovarian cancer.*
- *To investigate the pattern of recurrence and treatment outcomes according to clinical variables including genomic status including BRCA1 or BRCA2 mutation and immune-histochemical staining after intraoperative HIPEC vs. no HIPEC followed by intravenous chemotherapy in patients with primary advanced ovarian cancer.*

2.3 Sub-group analysis

- Sub-group analysis will be evaluated according to the use of neo-adjuvant chemotherapy, disease burden evaluated by synoptic operative template for ovarian cancer (SOTOC) score at surgery, and institute.

III. Study populations

3.1 Inclusion Criteria:

1. Disease status primary ovarian cancer, tubal cancer, and primary peritoneal cancer (Stage III or IV)

A. Primary debulking surgery group who are expected to be optimally debulked.

B. Three cycle of neoadjuvant chemotherapy with carboplatin and paclitaxel in case of difficult optimal debulking with primary surgery or in case with poor general condition.

2. Residual tumor < 1cm after completion of cytoreductive surgery

3. Age < 75 year

4. Expected survival > 3 months

5. Performance status: ECOG 0-1

6. Adequate bone marrow function Hb ≥8 g/dl (After correction in case of iron deficient anemia) WBC ≥ 3,000/mm3, Platelet ≥ 100,000/mm3

7. Adequate renal function Creatinine ≤ 1.5 mg/dl

8. Adequate hepatic function Bilirubin ≤ 1.5 mg/dl and AST and ALT ≤ 80 IU/L

9. Optimal cardiopulmonary function for surgery

10. Voluntary participation after getting written informed consent.

3.2 Exclusion Criteria:

1. Unresectable extraperitoneal metastasis (brain, bone, lung parenchyme, or lymph node)

2. Suboptimal debulking (residual tumor ≥ 1cm)

3. Previous history of other malignancies (except excision of skin cancer, thyroid cancer)

4. Serious heart disease or renal failure

5. Serious cardiopulmonary insufficiency

6. Uncontrolled infection

7. Uncontrolled intercurrent disease

8. Psychogenic disorder

9. Patients who are unsuitable candidates by legally

10. Pregnant or breast-feeding patients

11. Patients who are unsuitable candidates by doctor's decision

12. Cancer tissue is not confirmed during surgery after neo-adjuvant chemotherapy

IV. Sample size consideration

4.1 Total number of subjects: 184

4.2 We assumed that the median survival times are 1.5 year for control group and 2.0 year for treatment group, that is a hazard ratio (HR) of 0.75. The total duration of the study is 8 years, which is the accrual time of 6 years plus the follow-up period of 2 years. A log-rank test with a total sample size of 184 subjects (per each group 92 subjects) achieves 82.3% power at a one-sided 0.20 significance level to detect a HR of 0.75. We additionally calculated that a log-rank test with the sample size of 184 subjects achieves 68.8% power at one-sided 0.1 significance level to detect a HR of 0.75. This result was calculated using the PASS version 2019.

4.3 Analysis populations

Baseline characteristics and treatment outcome will be analyzed based on the intent-to-treat (ITT) population, defined as all participants as randomized in the trial. The Per Protocol (PP) population is a subgroup who received HIPEC or no HPEC after surgery in this trial.

4.4 Randomization

Randomization will be performed centrally with equal probability of assignment to each treatment regimen.

V. Analysis of survival outcomes

5.1 The recurrence or progression is evaluated by the Response Evaluation Criteria in Solid Tumors (RECIST) criteria version 1.1 and/or CA125 Gynecologic Cancer InterGroup criteria (GCIG) [2-4].

5.2 The analysis of survival outcome will be evaluated by the Kaplan-Meier methods using the log-rank test or stratified log-rank test. Cox's proportional hazard regression model will be evaluated to estimate the treatment effect after adjusting for confounding covariates.

5.3 Survival analysis

- All the data will be recorded according to the case report form and will be analyzed statistically. Basic characteristics and important parameters of patients are summarized using descriptive statistics. For categorical variables, cross-tabulation and Chi-square test or the Fisher's exact test will be used. For continuous variables, mean and standard deviation or median and range will be used.
- Complications such as anastomotic leakage, abscess, postoperative hemorrhage, sepsis, pancreatitis, pneumonia, neurological complications, bone marrow toxicity, and nephrotoxicity will be divided into postoperative period and after adjuvant chemotherapy. Mortality during intended treatment including operation (±HIPEC) and adjuvant chemotherapy will be specifically described. Complications and surgical mortality are presented as frequency and analyzed using Chi-square test or Fisher's exact test when differences are found according to possible clinical prognostic factors.
- Prognostic factors will be evaluated: age, use of neoadjuvant chemotherapy, ovarian cancer stage, histologic type, degree of differentiation, residual tumor size, and surgical history, operative time, intraoperative complications, the number of days after surgery, the days until gas out, the day before the dietary procedure, the chest tube insertion, the period until adjuvant chemotherapy, the

type of chemotherapy used, the medical comorbidities such as thrombosis, the time to first recurrence and recurrence. These factors will be evaluated using Cox's proportional hazard model.

- Survival outcome will be measured by Kaplan-Meier. Difference between two groups will be evaluated using log-rank test. The overall survival (OS) is the period from the randomization to the time of death from any causes. The progression-free survival (PFS) is the period from the randomization to the time of disease recurrence or progression or death from any causes. The recurrence-free survival (RFS) is the period from the randomization to the time of disease recurrence or progression of ovarian, fallopian or primary peritoneal carcinoma. Disease-free survival (DFS) is the period from the time of complete remission to the point at which evidence of disease reappears. The survival probability is estimated using the Kaplan-Meier method and the log-rank test is used to compare survival rates between groups in overall times. The Cox proportional hazards model is used to determine the relationship between other clinically important variables and the survival time, and the hazard ratio is used as the effect size. The presence or absence of complications is expressed as frequency and if necessary, the difference of frequency distribution is tested using Chi-square test or Fisher's exact test. Statistical significance is $p < 0.05$.
- The quality of life was assessed before HIPEC, 1 week after HIPEC, every 3 cycles after chemotherapy, and every 3 months until 1 year after chemotherapy and then the quality of life will be evaluated every year. The quality of life will be assessed using the EORTC QOL-C30, EORTC QOL-OV28, and MDASI. Raw data, frequency and an average value of the answer for each question and evaluation of the quality of life according to the designated surveillance interval will be assessed and collected.

5.4 Analysis of treatment related adverse events and toxicities

For analysis of treatment related adverse event and prognostic marker, the univariable analysis of each clinical or genomic variable will be performed to

determine the probability of association with each adverse event or treatment outcome. Continuous variables will be analyzed by using a Student’s t-test or Wilcoxon rank-sum test. Comparisons of the proportions of toxicities between the 2 arms will be done by use of a chi-square test or a two-sided Fisher’s exact test in cases of expected participants in a given category is less than 5.

REFERENCES

3) van Driel WJ, Koole SN, Sikorska K, Schagen van Leeuwen JH, Schreuder HWR, Hermans RHM, de Hingh IHJT1, van der Velden J, Arts HJ, Massuger LFAG, Aalbers AGJ, Verwaal VJ, Kieffer JM, Van de Vijver KK, van Tinteren H, Aaronson NK, Sonke GS. Hyperthermic Intraperitoneal Chemotherapy in Ovarian Cancer. N Engl J Med. 2018 Jan 18;378(3):230-240.

4) E.A. Eisenhauer; P. Therasse; J. Bogaerts; L.H. Schwartz; D. Sargent; R. Ford; J. Dancey; S. Arbuck; S. Gwyther; M. Mooney; L. Rubinstein; L. Shankar; L. Dodd; R. Kaplan; D. Lacombe; J. Verweij, "New response evaluation criteria in solid tumours: Revised RECIST guideline (version 1.1). European Journal of Cancer 2009; 45: 228–247.

5) Comparison of CA-125 and standard definitions of progression of ovarian cancer in the intergroup trial of cisplatin and paclitaxel versus cisplatin and cyclophosphamide. Rustin GJ, Timmers P, Nelstrop A, Shreeves G, Bentzen SM, Baron B, Piccart MJ, Bertelsen K, Stuart G, Cassidy J, Eisenhauer E. J Clin Oncol. 2006 Jan 1;24(1):45-51.

6) Rustin GJ, Vergote I, Eisenhauer E, et al. Definitions for response and progression in ovarian cancer clinical trials incorporating RECIST 1.1 and CA 125 agreed by the Gynecological Cancer Intergroup (GCIG). International journal of gynecological cancer: official journal of the International Gynecological Cancer Society 2011; 21: 419-23.
