# Supplementary material for: Survival After Hyperthermic Intraperitoneal Chemotherapy and Primary or Interval Cytoreductive Surgery in Ovarian Cancer: A Randomized Clinical Trial
Source: JAMA Surg. 2022 Mar 9;157(5):374–83. doi: 10.1001/jamasurg.2022.0143 (PMC8908225; doi:10.1001/jamasurg.2022.0143)
Supplement: Supplement 2. — eTable 1. FIGO Staging Classification of Ovarian Cancer eTable 2. Peritoneal Carcinomatosis Index (PCI) eTable 3. Additional Baseline Patients’ Characteristics and Surgical and Post-surgical Outcomes eTable 4. Comparison of Adverse Events Before and After Use of Amifostine eTable 5. Univariable and Multivariable Cox Proportional Hazard Regression Models for PFS and OS eFigure 1. Study Population of GOG 172, OVHIPEC-1, and This Trial eFigure 2. Subgroup Analysis of Progression-Free Survival eFigure 3. Subgroup Analysis of Progression-Free Survival in Patients With Primary Cytoreductive Surgery eFigure 4. Subgroup Analysis of Progression-Free Survival in Patients With Interval Cytoreductive Surgery After Neoadjuvant Chemotherapy [file jamasurg-e220143-s002.pdf]

Supplementary Online Content

Lim MC, Chang S-J, Park B, et al. Survival after hyperthermic intraperitoneal chemotherapy and primary or interval cytoreductive surgery in ovarian cancer: a randomized clinical trial. *JAMA Surg.* Published online March 9, 2022.
doi:10.1001/jamasurg.2022.0143

eTable 1. FIGO Staging Classification of Ovarian Cancer
eTable 2. Peritoneal Carcinomatosis Index (PCI)
eTable 3. Additional Baseline Patients' Characteristics and Surgical and Post-surgical Outcomes
eTable 4. Comparison of Adverse Events Before and After Use of Amifostine
eTable 5. Univariable and Multivariable Cox Proportional Hazard Regression Models for PFS and OS
eFigure 1. Study Population of GOG 172, OVHIPEC-1, and This Trial
eFigure 2. Subgroup Analysis of Progression-Free Survival
eFigure 3 Subgroup Analysis of Progression-Free Survival in Patients With Primary Cytoreductive Surgery
eFigure 4 Subgroup Analysis of Progression-Free Survival in Patients With Interval Cytoreductive Surgery After Neoadjuvant Chemotherapy
eReferences

This supplemental material has been provided by the authors to give readers additional information about their work.

© 2022 Lim MC et al. *JAMA Surgery*.

**eTable 1. FIGO Staging Classification of Ovarian Cancer (1988)[3]**	
I	Growth limited to the ovaries
IA	Tumor limited to one ovary; capsule intact, no tumor on ovarian surface; no malignant cells in ascites or peritoneal washings
IB	Tumor limited to both ovaries; capsule intact, no tumor on ovarian surface; no malignant cells in ascites or peritoneal washings
IC	Tumor limited to one or both ovaries; capsule ruptured, tumor on ovarian surface; malignant cells in ascites or peritoneal washings
II	Tumor involves one or both ovaries with pelvic extension
IIA	Extension and/or implants on uterus and/or tube(s)
IIB	Extension to other pelvic tissues
IIC	Pelvic extension with any of the following: capsule ruptured, tumor on ovarian surface, malignant cells in ascites or peritoneal washings
III	Tumor involves one or both ovaries with peritoneal metastasis outside the pelvis and/or retroperitoneal or inguinal lymph node metastasis
IIIA	Microscopic peritoneal metastasis beyond pelvis
IIIB	Microscopic peritoneal metastasis beyond pelvis 2cm or less in greatest dimension
IIIC	Peritoneal metastasis beyond pelvis more than 2cm in greatest dimension and/or positive retroperitoneal or inguinal lymph nodes
IV	Growth involving one or both ovaries with distant metastases. If pleural effusion is present, there must be positive cytology to allot a case to Stage IV. Parenchymal liver metastasis equals Stage IV
Abbreviation: FIGO, International Federation of Gynecology and Obstetrics.	

© 2022 Lim MC et al. *JAMA Surgery*.

eTable 2. Peritoneal Carcinomatosis Index (PCI)

No.	Score	Area of tumor involvement
1	1	Omentum
2	1	Left upper quadrant (Spleen, pancreas, and left diaphragm)
3	1	Epigastric
4	1	Right upper quadrant (liver, Morrison's pouch, and right diaphragm)
5	1	Colon
6	1	Small Bowel
7	1	Paracolic gutter
8	1	Pelvis (Ovary and pelvic peritoneum)
9	1	Lymph node (Intra-peritoneal and extra-peritoneal)
10	1	Others (Pleura, Lung, Others)

Modification from our previous publication on the preoperative intraperitoneal tumor burden.[4]

© 2022 Lim MC et al. *JAMA Surgery*.

eTable 3. Additional Baseline Patients' Characteristics and Surgical and Post-surgical Outcomes.

Variables		All patients		Primary cytoreductive Surgery		Interval cytoreductive Surgery	
		Control	HIPEC	Control	HIPEC	Control	HIPEC
		(N=92)	(N=92)	(N=49)	(N=58)	(N=43)	(N=34)
Institution							
	National Cancer Center	86 (93.5)	85 (92.4)	49 (100.0)	55 (94.8)	37 (86.0)	30 (88.2)
	Ajou University Hospital	6 (6.5)	7 (7.6)	0 (0.0)	3 (5.2)	6 (14.0)	4 (11.8)
Performance status							
	0	51 (55.4)	58 (63.0)	33 (67.4)	40 (69.0)	18 (41.9)	18 (52.9)
	1	41 (44.6)	34 (37.0)	16 (32.7)	18 (31.0)	25 (58.1)	16 (47.1)
Body mass index	median (IQR)	22.7 (21.1-24.1)	22.7 (21.1-24.5)	23.0 (20.9-24.9)	22.4 (20.9-23.7)	22.3 (21.2-23.9)	24.1 (21.4-25.8)
Baseline CA125	median (IQR)	227.5 (44.2-1549.5)	458.5 (84.9-1219)	530 (187-2470)	870 (412.4-1426)	52.3 (24.7-361)	64.1 (21.9-298)
Pre-treatment CA125	median (IQR)	941 (356-3651.2)	1195 (482.5-3026.5)	530 (187-2470)	870 (412.4-1426)	1259 (535-4650)	2105 (1255-4310)
EBL	median (IQR)	715 (500-1200)	800 (500-1300)	800 (500-1400)	850 (550-1400)	600 (500-1000)	600 (400-1200)
Standard procedures	Yes	91 (98.9)	92 (100.0)	48 (98.0)	58 (100.0)	43 (100.0)	34 (100.0)
Hysterectomy	Yes	86 (93.5)	85 (92.4)	47 (95.9)	55 (94.8)	39 (90.7)	30 (88.2)
Salpingo-Oophorectomy	Yes	85 (92.4)	90 (97.8)	46 (93.9)	57 (98.3)	39 (90.7)	33 (97.1)
Pelvic LN dissection	Yes	89 (96.7)	89 (96.7)	48 (98.0)	57 (98.3)	41 (95.4)	32 (94.1)
Paraaortic LN dissection	Yes	90 (97.8)	90 (97.8)	48 (98.0)	58 (100.0)	42 (97.7)	32 (94.1)
Omentectomy	Yes	85 (92.4)	85 (92.4)	46 (93.9)	54 (93.1)	39 (90.7)	31 (91.2)
Appendectomy	Yes	81 (88.0)	79 (85.9)	42 (85.7)	51 (87.9)	39 (90.7)	28 (82.4)
Extensive procedures	yes	89 (96.7)	90 (97.8)	48 (98.0)	58 (100.0)	41 (95.4)	32 (94.1)
Splenectomy	yes	34 (37.0)	40 (43.5)	20 (40.8)	29 (50.0)	14 (32.6)	11 (32.4)
Distal pancreatectomy	yes	1 (1.1)	6 (6.5)	1 (2.0)	5 (8.6)	0 (0.0)	1 (2.9)
Liver tumorectomy	yes	11 (12.0)	8 (8.7)	8 (16.3)	6 (10.3)	3 (7.0)	2 (5.9)
Diaphragmatic stripping	yes	81 (88.0)	74 (80.4)	44 (89.8)	46 (79.3)	37 (86.1)	28 (82.4)
Small bowel resection	yes	12 (13.0)	17 (18.5)	10 (20.4)	10 (17.2)	2 (4.7)	7 (20.6)
Hemicolectomy	yes	3 (3.3)	8 (8.7)	3 (6.1)	4 (6.9)	0 (0.0)	4 (11.8)
Total colectomy	yes	4 (4.4)	6 (6.5)	2 (4.1)	5 (8.6)	2 (4.7)	1 (2.9)

© 2022 Lim MC et al. *JAMA Surgery*.

Low anterior resection	yes	62 (67.4)	64 (69.6)	34 (69.4)	43 (74.1)	28 (65.1)	21 (61.8)
Ileostomy	yes	6 (6.5)	7 (7.6)	4 (8.2)	4 (6.9)	2 (4.7)	3 (8.8)
ICU admission - no. (%)	yes	92 (100.0)	92 (100.0)	49 (100.0)	58 (100.0)	43 (100.0)	34 (100.0)
Use of Amifostine in HIPEC group (n=92)							
	no	.	71 (77.2)	.	47 (81.0)	.	24 (70.6)
	yes	.	21 (22.8)	.	11 (19.0)	.	10 (29.4)
Thoracic drain inserted							
	No	78 (84.8)	81 (88.0)	40 (81.6)	52 (89.7)	38 (88.4)	29 (85.3)
	Unilateral	13 (14.1)	11 (12.0)	8 (16.3)	6 (10.3)	5 (11.6)	5 (14.7)
	Bilateral	1 (1.1)	0 (0.0)	1 (2.0)	0 (0.0)		
	No	86 (93.5)	87 (94.6)	45 (91.8)	55 (94.8)	41 (95.4)	32 (94.1)
	DJ catheter	6 (6.5)	5 (5.4)	4 (8.2)	3 (5.2)	2 (4.7)	2 (5.9)
	Abdomen	78 (84.8)	81 (88.0)	40 (81.6)	52 (89.7)	38 (88.4)	29 (85.3)
	Abdomen and Chest	14 (15.2)	11 (12.0)	9 (18.4)	6 (10.3)	5 (11.6)	5 (14.7)
Admission ICU days	median (IQR)	1 (1-1)	1 (1-1)	1 (1-1)	1 (1-1)	1 (1-1)	1 (1-1)
Admission to ward days	median (IQR)	13 (11-22.5)	16 (12-22)	14 (11-27)	15.5 (13-21)	13 (11-19)	16 (12-26)
Gastric retention days	median (IQR)	2 (1-2)	2 (1-2)	2 (1-2)	2 (1-2)	1 (1-2)	2 (2-2)
Gas out days	median (IQR)	3.5 (3-4)	4 (3-5)	4 (3-5)	4 (3-5)	3 (3-4)	3 (2-4)
Liquid diet days	median (IQR)	5 (4-6)	6 (4-6)	6 (4-6)	6 (4-6)	5 (4-6)	5 (4-6)
Regular diet days	median (IQR)	6 (5-7)	7 (5-7)	6 (5-7)	7 (6-7)	6 (5-7)	6 (4-7)
Parenteral nutrition days	median (IQR)	3 (2-4)	3 (1-4)	3 (2-4)	3 (2-5)	3 (2-4)	3 (1-4)

Abbreviation: DJ, double J; EBL, estimated blood loss; HIPEC, hyperthermic intraperitoneal chemotherapy; ICU, intensive care unit; IQR, interquartile range; JP, Jackson-Pratt; LN, lymph node.

According to the Eastern Cooperative Oncology Group (ECOG) performance-status evaluation, a score of 0 indicates that the patient is fully active and able to carry on all pre-disease performance without restriction, and a score of 1 indicates that the patient is restricted in physically strenuous activity but ambulatory and able to carry out work of a light or sedentary nature.

© 2022 Lim MC et al. *JAMA Surgery*.

eTable 4. Comparison of Adverse Events Before and After Use of Amifostine

Variables	No (N=71)		Yes (N=21)	
	Any Grade	Grade 3 or 4	Any Grade	Grade 3 or 4
Electrolyte disturbance	71 (100)	59 (83.1)	21 (100)	15 (71.4)
Anemia	71 (100)	37 (52.1)	21 (100)	15 (71.4)
Abdominal pain	71 (100)	0 (0)	21 (100)	0 (0)
Peripheral sensory neuropathy	62 (87.3)	0 (0)	16 (76.2)	0 (0)
Lymphocele	59 (83.1)	12 (16.9)	17 (81)	4 (19)
Hyper aminotransferase	59 (83.1)	7 (9.9)	19 (90.5)	1 (4.8)
Pulmonary	59 (83.1)	7 (9.9)	10 (47.6)	1 (4.8)
Increased prothrombin time	59 (83.1)	1 (1.4)	16 (76.2)	0 (0)
Creatinine increased	58 (81.7)	2 (2.8)	5 (23.8)	0 (0)
Nausea	53 (74.6)	0 (0)	15 (71.4)	0 (0)
White blood cell decreased	52 (73.2)	22 (31)	15 (71.4)	2 (9.5)
Neutrophil count decreased	50 (70.4)	29 (40.8)	13 (61.9)	2 (9.5)
Anorexia	45 (63.4)	4 (5.6)	9 (42.9)	1 (4.8)
Infection	40 (56.3)	25 (35.2)	7 (33.3)	2 (9.5)
Cardiac	40 (56.3)	2 (2.8)	8 (38.1)	0 (0)
Insomnia	38 (53.5)	2 (2.8)	9 (42.9)	0 (0)
Diarrhea	36 (50.7)	16 (22.5)	7 (33.3)	4 (19)
Vomiting	33 (46.5)	1 (1.4)	5 (23.8)	0 (0)
Constipation	30 (42.3)	0 (0)	5 (23.8)	0 (0)
Platelet count decreased	24 (33.8)	7 (9.9)	9 (42.9)	1 (4.8)
Blood bilirubin increased	24 (33.8)	2 (2.8)	2 (9.5)	0 (0)
Hypertension	21 (29.6)	8 (11.3)	8 (38.1)	3 (14.3)
Ileus	21 (29.6)	7 (9.9)	5 (23.8)	2 (9.5)
Wound dehiscence	19 (26.8)	12 (16.9)	3 (14.3)	2 (9.5)
Acute kidney injury	19 (26.8)	2 (2.8)	0 (0)	0 (0)
Lymphedema	17 (23.9)	0 (0)	6 (28.6)	0 (0)
Urticaria	11 (15.5)	2 (2.8)	8 (38.1)	2 (9.5)
Febrile neutropenia	9 (12.7)	9 (12.7)	0 (0)	0 (0)
Depression	8 (11.3)	0 (0)	0 (0)	0 (0)
Anal hemorrhage	5 (7)	1 (1.4)	1 (4.8)	0 (0)
Thromboembolic event	5 (7)	0 (0)	3 (14.3)	1 (4.8)
Sepsis	3 (4.2)	3 (4.2)	0 (0)	0 (0)
Colonic fistula	2 (2.8)	2 (2.8)	0 (0)	0 (0)
Delirium	2 (2.8)	1 (1.4)	0 (0)	0 (0)
Intra-abdominal hemorrhage	2 (2.8)	0 (0)	1 (4.8)	1 (4.8)
Fall	2 (2.8)	0 (0)	1 (4.8)	1 (4.8)
Urinary fistula	1 (1.4)	1 (1.4)	0 (0)	0 (0)
Colonic perforation	1 (1.4)	1 (1.4)	1 (4.8)	1 (4.8)
Large intestinal anastomotic leak	1 (1.4)	1 (1.4)	1 (4.8)	0 (0)
Syncope	1 (1.4)	1 (1.4)	1 (4.8)	1 (4.8)
Seizure	1 (1.4)	1 (1.4)	0 (0)	0 (0)
Duodenal perforation	1 (1.4)	0 (0)	0 (0)	0 (0)
Postoperative hemorrhage	0 (0)	0 (0)	0 (0)	0 (0)
Jejunal hemorrhage	0 (0)	0 (0)	1 (4.8)	1 (4.8)

© 2022 Lim MC et al. *JAMA Surgery*.

eTable 5. Univariable and multivariable Cox proportional hazard regression models for PFS and OS

Variables		Progression-Free Survival (PFS)				Overall Survival (OS)			
		Univariable model		Multivariable model		Univariable model		Multivariable model	
		HR (95% CI)	P-value	HR (95% CI)	P-value	HR (95% CI)	P-value	HR (95% CI)	P-value
Group									
	Control	1 (ref)		1 (ref)		1 (ref)		1 (ref)	
	HIPEC	0.88 (0.63-1.21)	0.4268	0.87 (0.62-1.20)	0.3845	0.88 (0.58-1.32)	0.521	0.89 (0.59-1.34)	0.5677
Age, years		1.02 (1.00-1.03)	0.0822			1.03 (1.01-1.06)	0.003	1.02 (1.00-1.05)	0.0483
Serum albumin (g/dL)		0.96 (0.67-1.38)	0.8218			0.97 (0.62-1.53)	0.8995		
FIGO Stage									
	III	1 (ref)				1 (ref)			
	IV	1.34 (0.96-1.86)	0.0862			1.36 (0.90-2.06)	0.1467		
Histology									
	Serous	1 (ref)				1 (ref)			
	Endometrioid	0.41 (0.15-1.11)	0.0777			0.59 (0.19-1.87)	0.3708		
	Clear cell	1.04 (0.26-4.19)	0.962			0.68 (0.09-4.85)	0.6964		
	Others	1.50 (0.70-3.22)	0.2945			1.71 (0.74-3.94)	0.2078		
Neoadjuvant chemotherapy									
	No	1 (ref)		1 (ref)		1 (ref)		1 (ref)	
	Yes	1.96 (1.40-2.74)	<.0001	2.07 (1.47-2.90)	<.0001	1.97 (1.30-2.98)	0.0015	1.77 (1.16-2.70)	0.0084
Peritoneal carcinomatosis index score									
	0-5	1 (ref)		1 (ref)		1 (ref)			
	6-10	1.71 (1.15-2.53)	0.0081	1.70 (1.13-2.54)	0.0105	1.34 (0.83-2.19)	0.2339		
Residual disease after cytoreductive surgery									
	Microscopic	1 (ref)		1 (ref)		1 (ref)		1 (ref)	
	Macroscopic	2.01 (1.31-3.08)	0.0015	1.80 (1.16-2.79)	0.0083	2.34 (1.43-3.84)	0.0007	2.06 (1.25-3.40)	0.0048
Bowel surgery									
	No	1 (ref)				1 (ref)			
	Yes	1.21 (0.81-1.81)	0.3517			1.03 (0.62-1.70)	0.9233		
Rectosigmoid resection									
	No	1 (ref)				1 (ref)			
	Yes	1.22 (0.85-1.74)	0.2856			1.04 (0.66-1.64)	0.8642		

HR; hazard ratio, CI; confidence interval

© 2022 Lim MC et al. *JAMA Surgery*.

eFigure 1. Study Population of GOG 172, OVHIPEC-1, and This Trial[1,2]

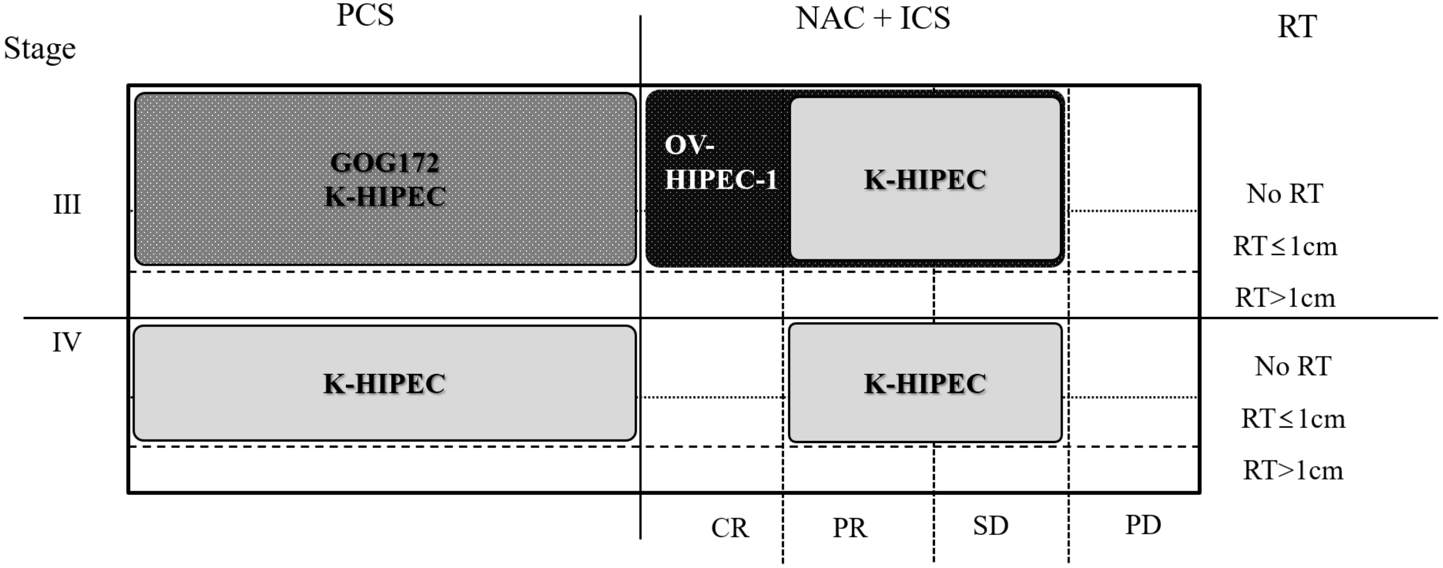

Figure S1. Show that the study population of GOG 172 includes patients with Stage III epithelial ovarian cancer and residual tumor size of 1cm or less after primary cytoreductive surgery. Whereas OV-HIPEC-1 trial includes patients with Stage III epithelial ovarian cancer who underwent interval cytoreductive surgery post-neoadjuvant chemotherapy except for progressive disease with neoadjuvant chemotherapy. This trial could compare the treatment outcomes of HIPEC after primary or interval cytoreductive surgery and involve Stage III and IV epithelial ovarian cancer as well.

Abbreviation: CR, complete remission; ICS, interval cytoreductive surgery; K-HIPEC, Korean-Hyperthermic Intraperitoneal Chemotherapy; NAC, neoadjuvant chemotherapy; PCS, primary cytoreductive surgery; PD, progressive disease; PR, partial remission; RT, residual tumor; SD, stable disease.

© 2022 Lim MC et al. *JAMA Surgery*.

eFigure 2 Subgroup Analysis of Progression-free Survival

Subgroup	no. of events/total no. (%) Control	HIPEC	Hazard Ratio (95% CI)
Age			
<55	38/50 (76.0)	38/51 (74.5)	0.87 (0.55-1.36)
≥55	36/42 (85.7)	33/41 (80.5)	0.91 (0.56-1.46)
Stage			
III	37/51 (72.6)	46/60 (76.7)	1.07 (0.69-1.65)
IV	37/41 (90.2)	25/32 (78.1)	0.68 (0.41-1.13)
Histology			
High-grade serous	54/64 (84.4)	64/79 (81.0)	0.92 (0.64-1.32)
Others	20/28 (71.4)	7/13 (53.9)	0.57 (0.24-1.36)
Neoadjuvant chemotherapy - no. (%)			
No	34/49 (69.4)	43/58 (74.1)	1.16 (0.74-1.83)
Yes	40/43 (93.0)	28/34 (82.4)	0.60 (0.37-0.99)
PCI score			
0-5	20/29 (69.0)	12/22 (54.6)	0.59 (0.29-1.22)
6-10	54/63 (85.7)	59/70 (84.3)	0.99 (0.68-1.43)
Residual disease after cytoreductive surgery			
Microscopic	64/80 (80.0)	55/75 (73.3)	0.78 (0.54-1.12)
Macroscopic	10/12 (83.3)	16/17 (94.1)	1.32 (0.59-2.94)
Bowel surgery - no. (%)			
No	17/24 (70.8)	13/19 (68.4)	0.73 (0.35-1.50)
Yes	57/68 (83.8)	58/73 (79.5)	0.92 (0.64-1.33)
Rectosigmoid resection			
No	21/30 (70.0)	21/28 (75.0)	0.89 (0.49-1.64)
Yes	53/62 (85.5)	50/64 (78.1)	0.87 (0.59-1.29)
Time between surgery and initiation of the adjuvant chemotherapy			
≤3 weeks	41/47 (87.2)	31/43 (72.1)	0.59 (0.37-0.94)
>3 weeks	32/44 (72.7)	40/49 (81.6)	1.28 (0.81-2.04)

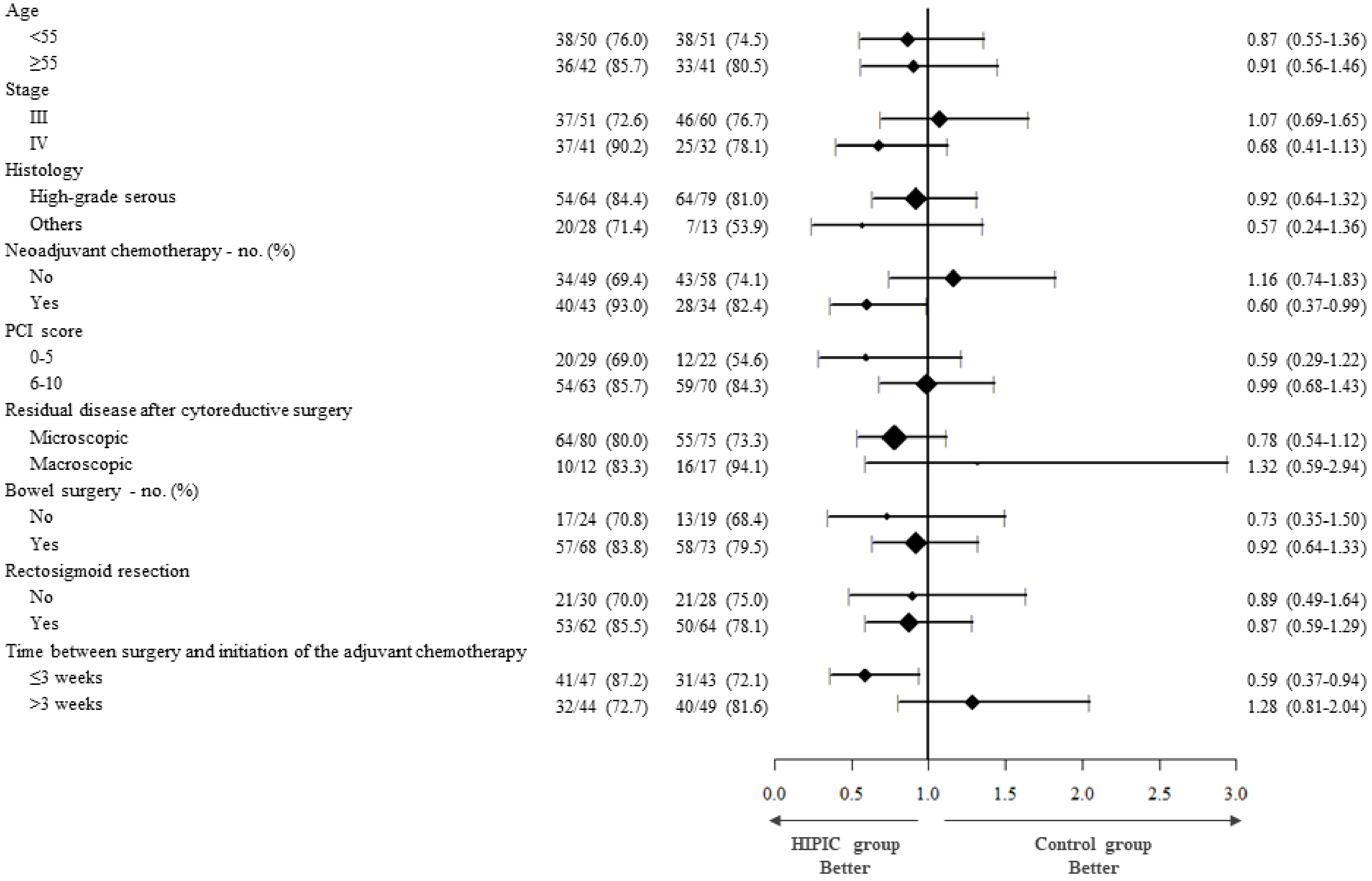

© 2022 Lim MC et al. *JAMA Surgery*.

eFigure 3. Subgroup Analysis of Progression-free Survival in Patients with Primary Cytoreductive Surgery

Subgroup	no. of events/total no. (%) Control	HIPEC	Hazard Ratio (95% CI)
Age			
<55	18/28 (64.3)	26/35 (74.3)	1.37 (0.75-2.50)
≥55	16/21 (76.2)	17/23 (73.9)	0.95 (0.48-1.89)
Stage			
III	22/34 (64.7)	34/45 (75.6)	1.39 (0.81-2.38)
IV	12/15 (80.0)	9/13 (69.2)	0.72 (0.30-1.74)
Histology			
High-grade serous	27/37 (73.0)	40/50 (80.0)	1.29 (0.79-2.10)
Others	7/12 (58.3)	3/8 (37.5)	0.52 (0.13-2.01)
PCI score			
0-5	9/16 (56.3)	9/14 (64.3)	1.14 (0.45-2.88)
6-10	25/33 (75.8)	34/44 (77.3)	1.16 (0.69-1.95)
Residual disease after cytoreductive surgery			
Microscopic	30/43 (69.8)	34/48 (70.8)	1.05 (0.64-1.71)
Macroscopic	4/6 (66.7)	9/10 (90.0)	1.82 (0.54-6.12)
Bowel surgery			
No	6/11 (54.6)	5/7 (71.4)	1.27 (0.38-4.18)
Yes	28/38 (73.7)	38/51 (74.5)	1.09 (0.67-1.78)
Rectosigmoid resection			
No	8/15 (53.3)	12/15 (80.0)	1.82 (0.74-4.48)
Yes	26/34 (76.5)	31/43 (72.1)	0.99 (0.58-1.66)
Time between surgery and initiation of the adjuvant chemotherapy			
≤3 weeks	19/22 (86.4)	22/30 (73.3)	0.69 (0.37-1.29)
>3 weeks	15/27 (55.6)	21/28 (75.0)	1.77 (0.91-3.44)

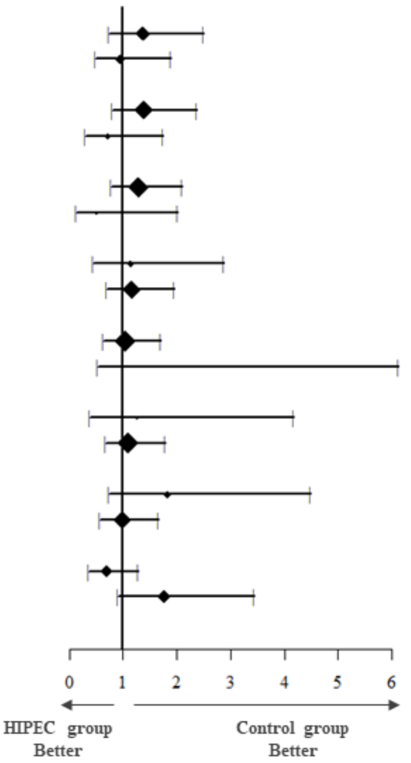

All subgroups presented here were predefined. For the hazard ratios, the size of the circle is proportional to the number of events.

Abbreviation: HIPEC, hyperthermic intraperitoneal chemotherapy; PCI, peritoneal carcinomatosis index.

© 2022 Lim MC et al. *JAMA Surgery*.

eFigure 4. Subgroup Analysis of Progression-free Survival in Patients with Interval Cytoreductive Surgery after Neoadjuvant Chemotherapy

Subgroup	no. of events/total no. (%) Control	HIPEC	Hazard Ratio (95% CI)
Age			
<55	20/22 (90.9)	12/16 (75.0)	0.39 (0.19-0.83)
≥55	20/21 (95.2)	16/18 (88.9)	0.87 (0.45-1.70)
Stage			
III	15/17 (88.2)	12/15 (80.0)	0.59 (0.27-1.30)
IV	25/26 (96.2)	16/19 (84.2)	0.63 (0.33-1.18)
Histology			
High-grade serous	27/27 (100.0)	24/29 (82.8)	0.49 (0.28-0.88)
Others	13/16 (81.3)	4/5 (80.0)	0.73 (0.24-2.24)
PCI score			
0-5	11/13 (84.6)	3/8 (37.5)	0.22 (0.06-0.79)
6-10	29/30 (96.7)	25/26 (96.2)	0.82 (0.47-1.41)
Residual disease after cytoreductive surgery			
Microscopic	34/37 (91.9)	21/27 (77.8)	0.52 (0.30-0.91)
Macroscopic	6/6 (100.0)	7/7 (100.0)	0.81 (0.26-2.55)
Bowel surgery			
No	11/13 (84.6)	8/12 (66.7)	0.35 (0.14-0.88)
Yes	29/30 (96.7)	20/22 (90.9)	0.81 (0.45-1.46)
Rectosigmoid resection			
No	13/15 (86.7)	9/13 (69.2)	0.38 (0.16-0.91)
Yes	27/28 (96.4)	19/21 (90.5)	0.78 (0.43-1.44)
Time between surgery and initiation of the adjuvant chemotherapy			
≤3 weeks	22/25 (88.0)	9/13 (69.2)	0.57 (0.26-1.25)
>3 weeks	17/17 (100.0)	19/21 (90.5)	0.58 (0.29-1.15)

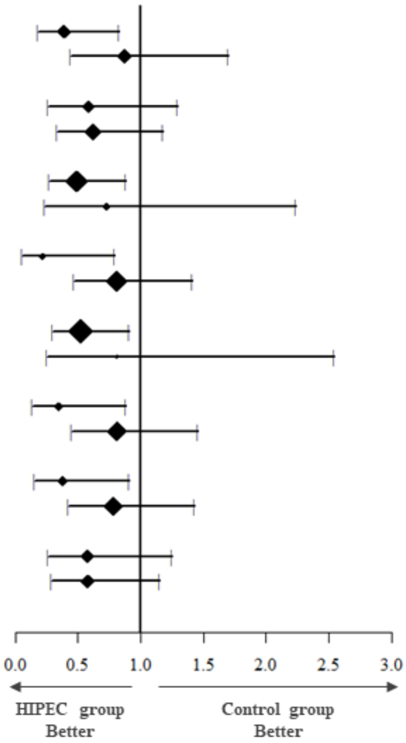

Assessment in the patients with post-neoadjuvant chemotherapy HIPEC, the results of subgroup analyses for progression-free survival showed a benefit with younger age (hazard ratio, 0.39; 95% CI, 0.19–0.83; P=0.01), high-grade serous histology (hazard ratio, 0.49; 95% CI, 0.28–0.88; P=0.01), lower peritoneal carcinomatosis index (hazard ratio, 0.22; 95% CI, 0.06–0.79; P=0.02), lower residual tumor (hazard ratio, 0.52; 95% CI, 0.30–0.91; P=0.02), no bowel surgery (hazard ratio, 0.35; 95% CI, 0.14–0.88; P=0.03), and no rectosigmoid resection (hazard ratio, 0.38; 95% CI, 0.16–0.91; P=0.03).

© 2022 Lim MC et al. *JAMA Surgery*.

eReferences

1. van Driel WJ, Koole SN, Sikorska K, et al. Hyperthermic Intraperitoneal Chemotherapy in Ovarian Cancer. *N Engl J Med.* 2018;378(3):230-240.
2. Armstrong DK, Bundy B, Wenzel L, et al. Intraperitoneal cisplatin and paclitaxel in ovarian cancer. *N Engl J Med.* 2006;354(1):34-43.
3. Pecorelli S, Benedet JL, Creasman WT, Shepherd JH. FIGO staging of gynecologic cancer. 1994-1997 FIGO Committee on Gynecologic Oncology. International Federation of Gynecology and Obstetrics. *Int J Gynaecol Obstet.* 1999;65(3):243-249.
4. Choi HJ, Lim MC, Bae J, et al. Region-based diagnostic performance of multidetector CT for detecting peritoneal seeding in ovarian cancer patients. *Arch Gynecol Obstet.* 2011;283(2):353-360.

© 2022 Lim MC et al. *JAMA Surgery*.
