# Supplementary material for: Survival After Hyperthermic Intraperitoneal Chemotherapy and Primary or Interval Cytoreductive Surgery in Ovarian Cancer: A Randomized Clinical Trial
Source: JAMA Surg. 2022 Mar 9;157(5):374–83. doi: 10.1001/jamasurg.2022.0143 (PMC8908225; doi:10.1001/jamasurg.2022.0143)
Supplement: Supplement 3. — Nonauthor Collaborators [file jamasurg-e220143-s003.pdf]

Supplemental Online Content: Nonauthor Collaborators

*Indicates required information. Only first name, last name, and suffix will appear in PubMed.

*Group Name(s): The HIPEC for Ovarian Cancer Collaborators

***First Name and Middle Initial(s)**	***Last Name**	***Suffix (eg, Jr, III)**	Academic Degrees	Institution	Location (city, state/province, country)	Role or Contribution, eg, chair, principal investigator	Group (if more than 1 Group listed in the byline) and/or Subgroup (eg, Steering Committee)
Sang-Soo	Seo		MD, PhD	Center for Gynecologic Cancer, National Cancer Center	Goyang, Gyeonggi, South Korea	Cytoreductive Surgica l Team	
Sokbom	Kang		MD, PhD	Center for Gynecologic Cancer, National Cancer Center	Goyang, Gyeonggi, South Korea	Cytoreductive Surgica l Team	
Jung Yeon	Yun		MD, PhD	Department of Anesthesiology, National Cancer Center	Goyang, Gyeonggi, South Korea	Anesthesiology team	
Dae-Soon	Cho		MD, PhD	Department of Anesthesiology, National Cancer Center	Goyang, Gyeonggi, South Korea	Anesthesiology team	
Sun Ho	Kim		MD, PhD	Department of Radiology, National Cancer Center	Goyang, Gyeonggi, South Korea	Radiologic evaluation	
Tae-Sung	Kim		MD, PhD	Department of Nuclear Medicine, National Cancer Center	Goyang, Gyeonggi, South Korea	Nuclear Medicine	
Sung Sil	Park		MD	Center for Colorectal Cancer, National Cancer Center	Goyang, Gyeonggi, South Korea	Cytoreductive Surgica l Team	
Dong Woon	Lee		MD	Center for Colorectal Cancer, National Cancer Center	Goyang, Gyeonggi, South Korea	Cytoreductive Surgica l Team	
Sung Chan	Park		MD, PhDc	Center for Colorectal Cancer, National Cancer Center	Goyang, Gyeonggi, South Korea	Cytoreductive Surgica l Team	
Hyeong Min	Park		MD	Center for Liver and Pancreatobiliary Cancer, National Cancer Center	Goyang, Gyeonggi, South Korea	Cytoreductive Surgica l Team	
Sung-Sik	Han		MD, PhD	Center for Liver and Pancreatobiliary Cancer, National Cancer Center	Goyang, Gyeonggi, South Korea	Cytoreductive Surgica l Team	
Seoung Hoon	Kim		MD, PhD	Center for Liver and Pancreatobiliary Cancer, National Cancer Center	Goyang, Gyeonggi, South Korea	Cytoreductive Surgica l Team	

Supplemental Online Content: Nonauthor Collaborators

*Indicates required information. Only first name, last name, and suffix will appear in PubMed.

***First Name and Middle Initial(s)**	***Last Name**	***Suffix (eg, Jr, III)**	Academic Degrees	Institution	Location (city, state/province, country)	Role or Contribution, eg, chair, principal investigator	Group (if more than 1 Group listed in the byline) and/or Subgroup (eg, Steering Committee)
Hee Chul	Yang		MD, PhD	Center for Lung Cancer, National Cancer Center	Goyang, Gyeonggi, South Korea	Cytoreductive Surgica l Team	
Moon Soo	Kim		MD, PhD	Center for Lung Cancer, National Cancer Center	Goyang, Gyeonggi, South Korea	Cytoreductive Surgica l Team	
Jong Mog	Lee		MD, PhD	Center for Lung Cancer, National Cancer Center	Goyang, Gyeonggi, South Korea	Cytoreductive Surgica l Team	
Bang Wool	Eom		MD, PhD	Center for Gastric Cancer, National Cancer Center	Goyang, Gyeonggi, South Korea	Cytoreductive Surgica l Team	
Young Il	Kim		MD, PhD	Center for Gastric Cancer, National Cancer Center	Goyang, Gyeonggi, South Korea	Preoperative evaluation team	
Hong Man	Yoon		MD	Center for Gastric Cancer, National Cancer Center	Goyang, Gyeonggi, South Korea	Cytoreductive Surgica l Team	
Il Ju	Choi		MD, PhD	Center for Gastric Cancer, National Cancer Center	Goyang, Gyeonggi, South Korea	Preoperative evaluation team	
Sung Han	Kim		MD, PhD	Center for Urologic Cancer, National Cancer Center	Goyang, Gyeonggi, South Korea	Cytoreductive Surgica l Team	
Jae Young	Joung		MD, PhD	Center for Urologic Cancer, National Cancer Center	Goyang, Gyeonggi, South Korea	Cytoreductive Surgica l Team	
Ho Kyung	Seo		MD, PhD	Center for Urologic Cancer, National Cancer Center	Goyang, Gyeonggi, South Korea	Cytoreductive Surgica l Team	
Jung Nam	Joo		PhD	Biometric Research Branch, Research Institute, National Cancer Center	Goyang, Gyeonggi, South Korea	Statistical service	
Yong Jung	Song		MD, PhD	Department of Obstetrics and Gynecology, Pusan National University Yangsan Hospital	Yangsan, South Korea	Cytoreductive Surgica l Team	
Sae Hyun	Park		MD	Department of Obstetrics and Gynecology, Incheon-Sarang Hospital	Incheon	Cytoreductive Surgica l Team	

Supplemental Online Content: Nonauthor Collaborators

*Indicates required information. Only first name, last name, and suffix will appear in PubMed.

***First Name and Middle Initial(s)**	***Last Name**	***Suffix (eg, Jr, III)**	Academic Degrees	Institution	Location (city, state/province, country)	Role or Contribution, eg, chair, principal investigator	Group (if more than 1 Group listed in the byline) and/or Subgroup (eg, Steering Committee)
Dae Chul	Jung		MD, PhD	Department of Radiology, Yonsei University College of Medicine	Seoul, South Korea	Preoperative evaluation team	
Min Jung	Kim		MD, PhD	Department of Surgery, Seoul National University College of Medicine	Seoul, South Korea	Cytoreductive Surgica l Team	
Ji Won	Park		MD, PhD	Department of Surgery, Seoul National University College of Medicine	Seoul, South Korea	Cytoreductive Surgica l Team	
Seung-Yong	Jeong		MD, PhD	Department of Surgery, Seoul National University College of Medicine	Seoul, South Korea	Cytoreductive Surgica l Team	
Young Ho	Yun		MD, PhD	Department of Family Medicine, National University College of Medicine	Seoul, South Korea	QOL evaluation team	
Sohee	Park		PhD	Epidemiology and Biostatistics, Graduate School of Public Health Yonsei University	Goyang, Gyeonggi, South Korea	Statistical service	
Robert E	Bristow		MD, MBA	Division of Gynecologic Oncology, Obstetrics and Gynecology, Irvine Medical Center, University of California	California, USA	Advisor	
