# Supplementary material for: Survival After Hyperthermic Intraperitoneal Chemotherapy and Primary or Interval Cytoreductive Surgery in Ovarian Cancer: A Randomized Clinical Trial
Source: JAMA Surg. 2022 Mar 9;157(5):374–83. doi: 10.1001/jamasurg.2022.0143 (PMC8908225; doi:10.1001/jamasurg.2022.0143)
Supplement: Supplement 4. — Data Sharing Statement [file jamasurg-e220143-s004.pdf]

Data Sharing Statement

Lim. Hyperthermic Intraperitoneal Chemotherapy for Ovarian Cancer. *JAMA Surg*. Published March 09, 2022. doi:10.1001/jamasurg.2022.0143

Data
Data available: Yes
Data types: Deidentified participant data
How to access data: mclim@ncc.re.kr.
When available: With publication

Supporting Documents
Document types: None

Additional Information
Who can access the data: External researchers can make written requests to the first author (MCL) or corresponding author (SYP) for sharing of data before publication or presentation. Requests with submitting a brief analysis plan and synopsis will be assessed and approved on a case-by-case basis in the research team. The personal identification deleted data will be sent in password-protected files. All data sharing will abide by rules and policies defined by the sponsor; relevant institutional review boards; and local, state, and federal laws and regulations. Data sharing mechanisms will ensure that the rights and privacy of individuals participating in research will be guaranteed.
Types of analyses: For scientific research, access to anonymized, individual and trial-level data (analysis data sets as long as the trials are not part of an ongoing or planned regulatory submission is possible. This includes requests for clinical trial data for unlicensed products and indications.
Mechanisms of data availability: Can be requested and will be provided following review and approval of a research proposal and Statistical Analysis Plan (SAP) and execution of a Data Sharing Agreement (DSA)
Any additional restrictions: Access is possible as long as the trials are not part of an ongoing or planned regulatory submission. Data will be accessible for 12 months, with possible extensions considered.
